# An enhanced genetic model of relapsed *IGH*-translocated multiple myeloma evolutionary dynamics

**DOI:** 10.1038/s41408-020-00367-2

**Published:** 2020-10-14

**Authors:** Phuc H. Hoang, Alex J. Cornish, Amy L. Sherborne, Daniel Chubb, Scott Kimber, Graham Jackson, Gareth J. Morgan, Gordon Cook, Ben Kinnersley, Martin Kaiser, Richard S. Houlston

**Affiliations:** 1 Division of Genetics and Epidemiology, The Institute of Cancer Research, London, SM2 5NG UK; 2 Division of Molecular Pathology, The Institute of Cancer Research, London, SM2 5NG UK; 3 Department of Haematology, University of Newcastle, Newcastle Upon Tyne, UK; 4 Perlmutter Cancer Center, NYU Langone Health, New York, USA; 5 Leeds Institute of Cancer and Pathology, University of Leeds, Leeds, UK

**Keywords:** Myeloma, Cancer genomics

## Abstract

Most patients with multiple myeloma (MM) die from progressive disease after relapse. To advance our understanding of MM evolution mechanisms, we performed whole-genome sequencing of 80 *IGH*-translocated tumour-normal newly diagnosed pairs and 24 matched relapsed tumours from the Myeloma XI trial. We identify multiple events as potentially important for survival and therapy-resistance at relapse including driver point mutations (e.g., *TET2*), translocations (*MAP3K14*), lengthened telomeres, and increased genomic instability (e.g., 17p deletions). Despite heterogeneous mutational processes contributing to relapsed mutations across MM subtypes, increased AID/APOBEC activity is particularly associated with shorter progression time to relapse, and contributes to higher mutational burden at relapse. In addition, we identify three enhanced major clonal evolution patterns of MM relapse, independent of treatment strategies and molecular karyotypes, questioning the viability of “evolutionary herding” approach in treating drug-resistant MM. Our data show that MM relapse is associated with acquisition of new mutations and clonal selection, and suggest APOBEC enzymes among potential targets for therapy-resistant MM.

## Introduction

Multiple myeloma (MM) is caused by the expansion of clonal plasma cells in the bone marrow^1^. Over half of MM tumours have chromosomal translocations involving the immunoglobulin heavy chain locus, which leads to overexpression of oncogenes (*CCND1*, *CCND3*, *MAF*, *MAFB*, *WHSC1*/*MMSET*, and *FGFR3*) as an initiating event^1^. Despite recent advances, MM is essentially an incurable malignancy, and most patients die from progressive disease after multiple relapses irrespective of treatment. Our limited knowledge of the molecular changes associated with relapse is a barrier to developing new therapeutic strategies to overcome drug resistance.

To advance our understanding of the evolution of MM tumours and the mutational mechanisms that shape their history, we performed whole-genome sequencing (WGS) of 80 newly diagnosed MM tumour-normal pairs, 24 also had matched relapsed tumours^2^. WGS allowed us to examine the impact of noncoding mutations, complex structural rearrangements, and telomere structure on MM tumourigenesis analyses not possible in previous studies, which have been based on whole-exome sequencing (WES)^3,4^. Integrating information from multiple types of genomic alterations has allowed us to infer the order of mutational events, and show that relapse is associated with acquisition of new mutations and clonal selection.

## Materials and methods

### Samples

Bone marrow aspirates and blood samples were obtained from 80 patients with newly diagnosed MM being treated according to the UK National Cancer Research Institute Myeloma XI trial protocol^2^. Matched relapsed tumour DNAs were available for 24/80 primary patients. Tumour DNAs were extracted from plasma cells selected and sorted using CD138 microbeads as described, previously^5^. In all cases tumour purity was in excess of 30%. Germline DNA was derived from matched blood samples. Tumour *IGH*-translocation status was determined using multiplexed real-time PCR^6^. Hyperdiploid MM was defined as gain of at least two chromosomes as defined previously^5^. An entire chromosome was considered amplified if at least 90% of the chromosome overlapped with an amplification^7^. Clinical data and informed consent was obtained from all patients. Ethical approval for the study was obtained by the Oxfordshire Research Ethics Committee (MREC 17/09/09, ISRCTN49407852).

### Whole genome sequencing

Sequencing libraries were prepared using Illumina SeqLab specific TruSeq Nano High Throughput library preparation kit (Illumina Inc, San Diego, CA 92122 USA), and paired end sequencing was conducted using Illumina HiSeqX technology. Raw WGS sequencing data were quality checked using FastQC (v.0.11.4) and aligned using the Burrows-Wheeler Alignment tool^8^ (BWA v0.7.13) to the human genome hg38 assembly using default parameters. Matching of tumour, normal, and relapsed samples was confirmed using NGSCheckMate^9^. Single nucleotide variants (SNVs) and indels were called using MuTect2 (v4.0.3.0)^10^ according to best practices, using The Genome Aggregation Database (gnomAD)^11^ file in GRCh38 provided as part of the GATK resource. Variants were filtered for cross-sample contamination, oxidation artefacts^10^, quality score^7^, and using a panel of normals generated from 80 germline samples. Variants with a germline population allele frequency >0.1% in gnomAD or in repetitive regions defined by University California Santa Cruz (UCSC) were excluded. Somatic indels were excluded if they were supported by <20% of tumour sample reads overlapping the position^12^ or were located within ten base pairs of a germline indel catalogued by gnomAD.

Reconstruction of clonal and subclonal copy number alterations (CNAs) for primary and relapsed tumours was conducted using Battenberg^13^. Since copy-neutral loss of heterozygosity (nLOH) is intrinsically more problematic to identify accurately^14^, these segments called by Battenberg were inspected manually against CNA calls overlapping within 10 Mb of two other CNA callers Sequenza^15^ and FACETS^16^. The copy number status of an nLOH segment was corrected and only reported if it was supported by at least two of the three CNA callers, and was excluded from downstream analysis if all methods were discordant. Tumour purity estimated by Battenberg was compared against and corrected using Ccube^17^. Somatic structural variants (SVs) were identified taking a consensus approach, as implemented by The Pancancer Analysis of Whole Genomes^18^, considering only variants identified by at least two of MANTA (v1.2.0)^19^, LUMPY (v0.2.13)^20^, or DELLY (v0.7.9)^21^. Chromothripsis regions were identified using ShatterSeek, adopting the criteria of at least four adjacent segments oscillating copy number states and at least six interleaved SVs^22^. All candidate chromothripsis regions were manually curated as previously advocated^22^. Chromoplexy was detected using ChainFinder (v1.0.1) with default parameters^23^ and hg38 UCSC cytoband definitions (http://hgdownload.cse.ucsc.edu/goldenpath/hg38/database/). As previously advocated^22^, chromoplexy was only called when at least three chromosomes were involved in a chain of SVs. Telomere length was estimated using Telomerecat^24^ with default parameters. Kataegis foci were identified using the KataegisPortal with default parameters (https://github.com/MeichunCai/KataegisPortal), and defined as having six or more consecutive mutations with an average mutational distance ≤1 Kb, excluding immune hypermutated regions^25^.

### Identifying driver mutations

Coding drivers were identified using dNdScv with default parameters^26^. Nonsilent mutations in a curated list of 82 established coding drivers^7,27^ and all coding genes were compared in matched primary and relapsed tumours. To identify noncoding drivers we analysed promoter and *cis*-regulatory regions (CREs) as described previously^7^. Briefly, promoters were defined as intervals spanning 400 bp upstream and 250 bp downstream of transcription start site from GENCODE (release 25)^28^. CREs were defined using promoter capture Hi-C data generated on naïve B-cells^29^. Raw sequencing reads from European Genome-Phenome Archive (EGA; accession code EGAS00001001911) were aligned to hg38 using HiCUP (v0.6.1)^30^ and promoter–CRE interactions were called with CHiCAGO (v1.8)^31^. Only interactions with linear distance ≤1 Mb and CHiCAGO score ≥5 were considered^7^.

Recurrently mutated promoters and CREs were identified using a Poisson binomial model as previously described^7,32^, taking into account tumour ID, trinucleotide context, and replication timing. For CRE regions, mutations were excluded if they overlap with open reading frames, 5′-UTR, and 3′-UTR as defined by Ensembl^7^. For promoters, mutations overlapping with open reading frames were excluded. Replication timing was estimated as the average of two B-lymphocyte replicates^33,34^. For promoters and CREs mutated in ≥3 samples, the clustering of mutations was examined using a permutation approach considering the number of mutations occurring at the same nucleotide position as previously described^7^. For each promoter and CRE, a combined *P*-value from the mutational recurrence and clustering analyses were obtained using Fisher’s method^7,35^. The Benjamini–Hochberg false discovery rate (FDR) procedure was used to adjust for multiple testing with significant threshold at *Q* < 0.05. Promoters and CREs overlap with immune hypermutated regions were excluded to avoid false positives. We only report CREs and promoters mutated in at least three tumours.

### Impact of cereblon and IMiD response pathway genes mutation on relapse

All patients we studied were treated with immunomodulatory drugs (IMiDs), either thalidomide or lenalidomide. Mutations in *CRBN* and associated genes have been proposed as being a mechanism of acquired drug resistance to IMiDs^36,37^. To examine this proposition, we specifically considered nonsynonymous mutations, CNAs, and SVs disrupting a curated list of 42 CRBN/IMiD genes—genes involved in the *CRBN* pathway regulation and IMiD response (Supplementary Table 1).

### Chronology of mutational events

The chronological timing of SNVs and CNAs was estimated independently for the 80 primary tumours as previously described^38^. Briefly, for SNVs we considered only driver genes mutated in ≥4 samples to allow reliable estimation of relative timing. For CNAs we considered only large-scale autosomal events (≥3 Mb) present in ≥8 samples^38^. Cytobands were assigned based on UCSC hg38 definitions. One sample (8573) displayed hyperdiploid characteristics and was excluded from the analysis. Cancer cell fractions (CCFs) of each CNV event and SNV were estimated using Battenberg^13^. Each cytoband or driver gene was ordered by mean of CCF from highest to lowest. The Tukey’s range test and a stepwise approach were used to test for difference between the CCF means of consecutive cytobands or driver genes to define discrete clonality levels, as described previously^38^. As previously advocated^38^, 95% confidence intervals were calculated with basic bootstrap method with 1000 iterations using boot R package.

### Analysis of copy number changes

Permutation was used to test the null hypothesis that the frequency of particular chromosome arm copy number events does not differ between primary and relapse MM. We first counted change in frequency of affected tumours at primary and relapse. We then randomly swapped condition labels for all matched primary and relapsed tumours 10,000 times, and recounted change in chromosome arm event frequency. Empirical *P*-values for each chromosome arm event were calculated as fraction of permutations with absolute net frequency change at least as great as the absolute net frequency change observed in the true primary/relapse labelling. We only considered chromosome arm events with net change in frequency in at least two tumours.

We employed a permutation-based approach to test the null hypothesis that additional relapse-associated CNA events occur by chance at pre-existing unstable genomic regions. For each autosomal chromosome arm, we counted the number of tumours with additional large-scale CNA on the considered chromosome arm at relapse. The tested chromosome arm in considered tumours with further CNA change were permutated 10,000 times among 44 possible chromosome arms loci (22 autosomal chromosomes with either p or q arm). The empirical *P*-values were calculated as the fraction of permutations with the number of additional CNA change were at least as great as the original tested chromosome arm.

### Mapping evolutionary trajectories

Analysis of clonality was conducted using only SNVs in diploid regions, as miscalled copy number states can confound the analysis. Potential neutral tail mutations were identified using MOBSTER^39^ and excluded prior to clustering procedure to minimise calling false positive clones. For each primary and relapse tumour pair, we performed two-dimensional variant clustering using a Bayesian Dirichlet process implemented in DPclust^3,13^. Only those clusters with ≥1% of total mutations and ≥100 SNVs were considered. Muller plots were generated with Timescape R package version 1.10.0. For each cluster in primary tumour and matched relapse, the proportion of SNVs shared was calculated.

### Mutational signatures

De novo extraction of signatures was performed on 80 primary and 24 relapsed genomes separately using non-negative matrix factorization^40^. We compared de novo mutational signatures with Catalogue of Somatic Mutations in Cancer (COSMIC) single base substitution (SBS) signatures version 3 by computing their cosine similarities^41^. A de novo mutational signature was assigned to a COSMIC signature if the cosine similarity was >0.75 as advocated^12^. We next performed signature fitting using deconstructSigs^42^ considering only those COSMIC signatures extracted de novo, as previously recommended^43^. In view of potential ambiguous assignment, we combined the contributions of the flat profile signatures 5, 8, and 40^25,42,43^, excluding signature 3 as this signature is unlikely to be active in MM^43^. As previously advocated, we compared mutational signature proportions in paired primary and relapsed samples using the chi-squared test^13^. Association between changes in mutational burden and AID/APOBEC mutational contribution for paired primary and relapsed tumours was calculated using Fisher’s exact test. Spearman correlation was performed to test the association between AID/APOBEC contribution of relapse-specific mutations and time to relapse.

## Results

We carried out WGS on 80 newly diagnosed MM tumour-normal pairs from the Myeloma XI trial, and matched relapsed tumour from 24 patients. The 80 patients had either *t*(4;14) (*n* = 38), *t*(11;14) (*n* = 38), or *t*(14;16) (*n* = 4) MM, with one patient carrying both *t*(4;14) translocation and trisomy of chromosomes 9 and 15 (Table 1). Hyperdiploid (HD) and non-HD subtypes of MM have distinctive genomic landscapes and are a priori likely to have different evolutionary trajectories^1^. In this study, we restricted our analysis to *IGH*-translocated tumours to focus on examining evolutionary dynamics of non-HD myeloma. WGS resulted in a median of 38× coverage for normal samples (30–44×), 111× for primary tumours (82–155×), and 114× for the 24 relapsed tumours (102–154×) (Supplementary Table 2). 6 of the 80 patients have been the subject of a previous WES project^4^.

**Table 1:** Summary of demographic and treatment data.

Sample ID	Karyotype	Gender	Age	Elapsed time (months)	Induction	Maintenance	Pathway
1305	11;14	Male	51	38.34	CTD	No	Intensive
1334	11;14	Female	43	24.00	CTD	Missing	Intensive
5834	11;14	Female	69	29.93	CTDa	No	Nonintensive
6030	4;14	Female	36	19.75	CTD	No	Intensive
6178	11;14	Female	67	18.40	RCD	Missing	Intensive
6229	11;14	Male	74	9.23	CTDa	Missing	Nonintensive
6706	11;14	Male	59	25.43	RCD	No	Intensive
6988	11;14	Male	69	12.26	RCDa	No	Nonintensive
7020	4;14	Female	58	14.69	CTD	Missing	Intensive
7240	4;14	Male	55	11.30	RCD	Lenalidomide	Intensive
7801	14;16	Female	48	14.49	CTD	Missing	Intensive
7842	4;14	Male	66	17.64	CTD	No	Intensive
8237	4;14	Female	49	14.00	CTD	No	Intensive
9126	11;14	Male	64	16.23	CTDa	Missing	Nonintensive
9166	14;16	Female	68	27.24	CCRD	No	Intensive
9515	11;14	Male	68	26.15	RCDa	Lenalidomide	Nonintensive
9721	14;16	Male	64	29.44	CTD	Lenalidomide	Intensive
10,068	4;14	Male	71	13.77	RCDa	Lenalidomide and Vorinostat	Nonintensive
10,365	11;14	Male	76	9.33	CTD	Missing	Intensive
11,506	14;16	Male	77	11.83	CTDa	Lenalidomide	Nonintensive
11,668	4;14	Male	49	19.29	RCDa	Missing	Nonintensive
11,949	11;14	Male	76	14.65	CTD	Missing	Intensive
12,546	4;14	Male	77	30.59	RCD	Missing	Intensive
13,029	4;14	Male	62	6.90	CTD	Missing	Intensive
5695	11;14	Male	64	NA	CTD	No	Intensive
5699	11;14	Female	68	NA	CTD	Missing	Intensive
5836	11;14	Male	77	NA	CTDa	No	Nonintensive
5939	4;14	Male	65	NA	CTD	Missing	Intensive
6016	11;14	Female	55	NA	RCD	Missing	Intensive
6076	4;14	Male	72	NA	RCDa	Lenalidomide	Nonintensive
6163	4;14	Male	75	NA	RCDa	Missing	Nonintensive
6277	11;14	Male	56	NA	RCD	Lenalidomide	Intensive
6279	4;14	Male	62	NA	RCD	Lenalidomide	Intensive
6345	4;14	Female	72	NA	CTDa	Missing	Nonintensive
6415	11;14	Female	68	NA	RCDa	Missing	Nonintensive
6425	4;14	Male	67	NA	RCD	Lenalidomide and Vorinostat	Intensive
6501	11;14	Female	51	NA	RCD	Missing	Intensive
6702	4;14	Female	78	NA	CTDa	Missing	Nonintensive
7000	11;14	Female	78	NA	CTDa	Missing	Nonintensive
7005	4;14	Male	74	NA	CTDa	Missing	Nonintensive
7164	11;14	Female	80	NA	RCDa	Missing	Nonintensive
7348	4;14	Male	67	NA	RCDa	No	Nonintensive
7729	4;14	Male	65	NA	RCD	Lenalidomide and Vorinostat	Intensive
7794	4;14	Female	52	NA	CTD	No	Intensive
7880	4;14	Female	82	NA	RCDa	Missing	Non-intensive
7915	4;14	Male	59	NA	CTD	Lenalidomide and Vorinostat	Intensive
7925	4;14	Male	59	NA	CTD	Missing	Intensive
7950	4;14	Male	49	NA	CTD	Lenalidomide and Vorinostat	Intensive
7956	4;14	Female	56	NA	CTD	Missing	Intensive
8043	4;14	Female	81	NA	CTDa	Missing	Non-intensive
8245	11;14	Female	63	NA	RCD	Lenalidomide	Intensive
8567	11;14	Female	66	NA	RCDa	Lenalidomide and Vorinostat	Nonintensive
8573	4;14/HD	Female	82	NA	CTDa	Missing	Nonintensive
8928	4;14	Male	52	NA	CTD	Missing	Intensive
8979	4;14	Male	76	NA	CTDa	Missing	Nonintensive
9069	11;14	Male	73	NA	RCDa	Missing	Non-intensive
9176	11;14	Male	78	NA	RCDa	Missing	Nonintensive
9210	11;14	Male	69	NA	CTD	Missing	Intensive
9249	11;14	Male	58	NA	RCD	Lenalidomide	Intensive
9289	11;14	Male	56	NA	CTD	No	Intensive
9292	4;14	Female	74	NA	CTDa	Missing	Nonintensive
9337	11;14	Female	71	NA	CTDa	Missing	Nonintensive
9376	4;14	Female	64	NA	RCD	Missing	Intensive
9409	11;14	Male	73	NA	CTDa	Missing	Nonintensive
9524	4;14	Male	51	NA	RCDa	Lenalidomide	Nonintensive
9544	11;14	Male	67	NA	RCDa	No	Nonintensive
9623	11;14	Male	58	NA	RCD	Lenalidomide	Intensive
9718	4;14	Male	66	NA	RCDa	No	Nonintensive
9917	11;14	Male	76	NA	CTDa	Missing	Nonintensive
9931	11;14	Female	55	NA	RCD	Missing	Intensive
10,085	11;14	Female	59	NA	CCRD	Lenalidomide	Intensive
10,212	11;14	Female	79	NA	RCDa	Lenalidomide	Nonintensive
10,597	4;14	Male	59	NA	CCRD	No	Intensive
10,772	4;14	Female	63	NA	CCRD	Missing	Intensive
10,801	11;14	Male	77	NA	RCDa	Missing	Nonintensive
11,029	4;14	Female	73	NA	RCDa	Missing	Nonintensive
11,897	4;14	Male	58	NA	CCRD	Lenalidomide	Intensive
12,101	4;14	Male	62	NA	CCRD	Missing	Intensive
12,227	11;14	Male	57	NA	CCRD	No	Intensive
12,541	11;14	Male	56	NA	CTD	Missing	Intensive

*CTD* cyclophosphamide, thalidomie, and dexamethasone, *CTDa* CTD with a reduced dose of dexamethasone and lower starting dose of thalidomide, *RCD* Lenalidomide (Revlimid), cyclophosphamide, and dexamethasone, *RCDa* RCD with a reduced dose of dexamethasone, *CCRD* carfilzomib, cyclophosphamide, lenalidomide, and dexamethasone. Intensive pathway: treatment with high dose melphalan after induction. NA: Matched relapsed data are not available.

### Mutational events in primary tumours

**Fig. 1:**
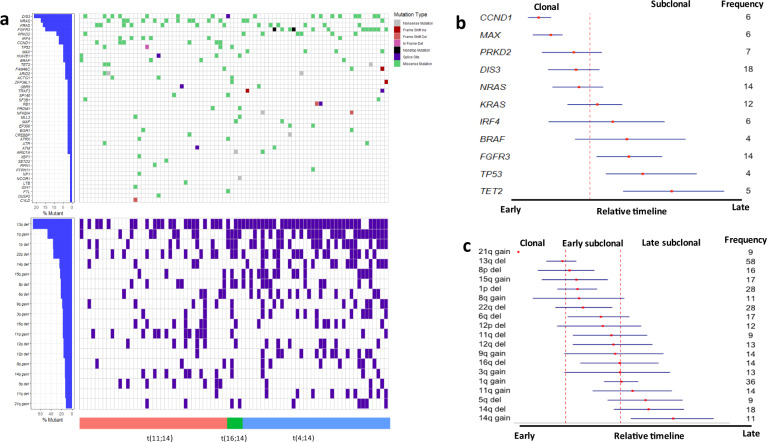
Frequency and chronology of coding drivers and major copy number events. **a** Frequency of coding drivers and major copy number events (present in at least eight tumours) detected in 80 primary tumours; **b**, **c** Chronology of coding drivers and major copy number events, respectively. Red dots denote mean of cancer cell fractions (CCFs) for each event with blue lines indicating 95% confidence intervals of the relative timing. Bootstrap confidence intervals were estimated based on the cancer cell fractions of mutational events. *X*-axis is plotted as relative timing based on CCF contribution. Dotted red lines denote discrete clonality events. Frequency: number of tumours with each mutational event; Ins insertion, Del deletion, LOH loss of heterozygosity.

We began by surveying for important genetic alterations in the 80 primary MM tumours by considering the contribution of both protein-coding and noncoding SNVs and indels, as well as CNAs. As expected, significantly mutated genes (*Q* < 0.05) at presentation were *DIS3*, *KRAS*, *NRAS*, *FGFR3*, *MAX*, *CCND1*, *TP53*, *IRF4*, and *PRKD2* (Fig. 1a and Supplementary Table 3). The promoters of 17 genes including *BCL6*, *CXCR4*, *BIRC3*, *MYO1E*, *CRIP1*, *FLT3LG*, and *DPP9* were also significantly mutated as well as nine *cis*-regulatory elements (CREs) interacting with genes including *PAX5*, *BCL6*, *ZCCHC7*, and *IFNGR1* (Supplementary Fig. 1 and Supplementary Tables 4, 5). The most frequent large-scale CNAs were deletion of 13q (73%), 22q (35%), and 1p (35%); and gain of 1q (45%). (Fig. 1a and Supplementary Fig. 2 and Supplementary Table 6). Aberrations of 13q was enriched in high-risk *t*(4;14) and *t*(14;16) MM (*P* = 3.5 × 10^−5^, odd ratio = 16.2, Fisher’s exact test).

Chromothripsis was observed in 18/80 primary tumours (23%) with the most frequently affected chromosomes are 1 (4 tumours), 8, 11, and 22 (3 tumours) (Supplementary Fig. 3); whereas 3% (2/80) of primary tumours featured chromoplexy (Supplementary Fig. 4). The frequency of chromothripsis and chromoplexy identified is comparable to a previous report^44^. Chromoplexy resulted in the simultaneous disruption of multiple driver genes^7,27^ (*KRAS*, *PRKD2*, *PTPN11*, *PTH2*, *BAX*, *CELA1*, *FTL*, *ARID2*, and *CDKN1B*) in primary tumours. Overall across the 80 primary tumours, high-risk subtypes MM t(4;14) and t(14;16) were associated with a shorter telomeres (*P* = 9.2 × 10^−5^, Wilcoxon rank-sum test) (Supplementary Fig. 5).

By integrating somatic mutations and copy number profiles we inferred the relative timing of key driver alterations in MM (i.e., which events occur earlier relative to others). Mutations of *CCND1*, *MAX*, *PRKD2*, *DIS3*, and *NRAS* were identified as early events whereas mutations of *KRAS*, *IRF4*, *FGFR3*, *TP53*, and *TET2* occurred as later events (Fig. 1b). Chronological timing of major CNAs (present in ≥10% of total samples)^38^ identified 21q gain and 13q deletion as being early events (Fig. 1c), consistent with a previous report that 13q deletions tend to be clonal^45^. 1p deletion and 1q gain, which has been linked to patient prognosis were identified as later events (Fig. 1c).

### Mutational landscape of relapse

We next investigated the molecular features of MM relapse by analysis of the 24 primary-relapse pairs. Patients received cyclophosphamide and dexamethasone in combination with either thalidomide (CTD), lenalidomide (RCD), or both carfilzomib and lenalidomide (CCRD) as induction therapy. Fit and young patients received high-dose melphalan (intensive pathway). 9 of the 25 patients subsequently received lenalidomide maintenance therapy. Treatment histories of each patient are summarized in Table 1. None of the patients we studied had detectable *CRBN* mutations at relapse. We did, however observe increased *IKZF3* mutation CCF and de novo mutations disrupting CRBN/IMiD genes in two patients at relapse—*RBX1* mutation and copy number loss affecting *UBE2A* (Supplementary Table 7). Relapse was associated with a higher mutational burden than primary tumours (Supplementary Fig. 6a–b, *P* < 0.01, paired Wilcoxon rank-sum test). Varied proportions (9–63%) of SNVs and indels identified in primary tumours were not detectable at relapse (Supplementary Fig. 6c), suggesting eradication and heterogenous clonal dynamics of the respective clone. Despite the increased mutational burden, relapsed tumours did not exhibit significantly more kataegis (Supplementary Fig. 7 and Supplementary Table 8). Chromothripis and chromoplexy were each observed in only one additional relapsed tumour (7842 and 8237 respectively; Supplementary Figs. 8 and 9). Although both primary and relapsed tumours had shorter telomeres compared to plasma cells (*P* < 0.01, paired Wilcoxon rank-sum test), relapse was associated with longer telomeres (*P* = 5.3 × 10^−3^) (Supplementary Fig. 10).

A translocation bringing the *IGH* loci in proximity to *MAP3K14* was gained at relapse in one tumour (Supplementary Fig. 11). Driver genes additionally mutated at relapse included *FAM46C*, *TRAF2*, *LTB*, *FAM154B*, *NF1*, *XBP1*, and *IDH2* (Supplementary Fig. 12). Driver mutations most frequently acquired at relapse were those in *KRAS* and *NRAS*, detected in three and two tumours respectively. The increase in CCF of *TET2* mutations implied selection of subclones (Supplementary Fig. 13). The promoters and CREs of an additional 16 genes were significantly mutated at relapse, including genes with established roles in the biology of MM or other B-cell malignancies such as *XBP1*, *BCL7A*, and *BCL9* (Supplementary Tables 9 and 10).

**Fig. 2:**
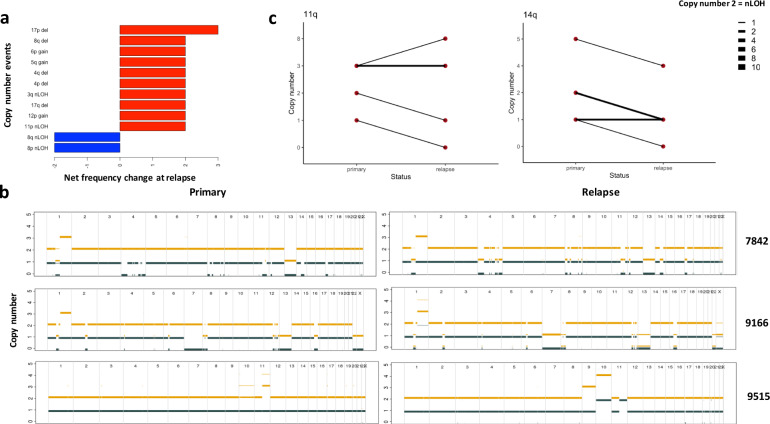
Copy number alterations associated with relapse. **a** Net change of CNA frequency in primary and matched relapse tumours; red and blue bars represent positive and negative changes respectively. Only significant events with changes in at least two tumours are shown. **b** Copy number profiles of patients 7842, 9166, and 9515. In 7842 copy number neutral loss of heterozygosity (nLOH) at chromosome 4 becomes LOH at relapse. In 9166 LOH at 13q progresses to complete loss of 13q. In 9515 copy number gain at chromosome 10 and 11 progresses to additional chromosome gain. Thick and thin lines represent clonal and subclonal copy number states, respectively. Yellow and blue lines denote total and minor copy number respectively (copy number states >5 not shown). **c** Patterns of copy number change across paired primary-relapse samples at 11q and 14q. Lines indicate relationship between primary and matched relapse tumours, with width being proportional to event frequency. Only chromosome arms with copy number alterations (CNAs) are plotted, with a copy number of 2 corresponding to nLOH.

Relapse was associated with additional CNAs, most frequently for 17p deletion (*P* < 2.2 × 10^−6^) (Fig. 2a, Supplementary Fig. 14, and Supplementary Table 11). We observed additional CNAs occurring at pre-existing unstable genomic regions, including the progression of copy-neutral loss of heterozygosity (nLOH) to LOH, LOH to complete deletion; as well as further copy number gains (Fig. 2b and Supplementary Fig. 15). Such trend was observed at a higher rate than expected by chance at 11q (*P* = 0.042) and 14q (*P* = 0.023) (Fig. 2c).

### Mutational processes active at relapse

At diagnosis, the major mutational signatures in tumours were those indicative of aging (SBS5), AID/APOBEC (SBS2, 9, and 13), and flat signatures (SBS5, 8, and 40) as previously observed^7,25^ (Supplementary Figs. 16 and 17). No additional mutational signatures potentially specific to treatment were extracted at relapse (Supplementary Fig. 18). Across all patients, we observed heterogeneous dynamic of mutational processes contributing to relapse (Supplementary Fig. 19). However, tumours with increased mutational burden at relapse were often associated with increased AID/APOBEC enzymes activity (*P* = 0.061, Fisher’s exact test). Despite the enrichment of APOBEC signatures in *t*(14;16) MM (*P* = 0.017, Wilcoxon rank-sum test) (Supplementary Fig. 17), we did not observe specific association of the signatures at relapse in this subtype (*P* = 0.20, Wilcoxon rank-sum test), consistent with previous finding^46^. Notably, patients with higher AID/APOBEC mutational contribution at relapse were associated with shorter refractory time (*r* = −0.43, *P* = 0.037, Spearman correlation) (Supplementary Figure 20). An increased C•G > G•C transversion rate in relapse-specific mutations was also observed (*Q* = 0.015, paired Wilcoxon rank-sum tests) (Supplementary Fig. 21), a feature previously reported in relapsed acute myeloid leukaemia^47^.

### Evolutionary trajectories of relapse

**Fig. 3:**
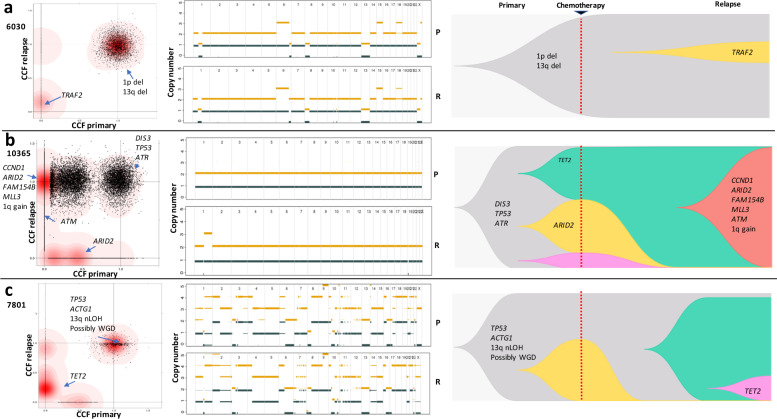
Evolutionary trajectories of relapse. **a** Pattern 1 (3/24), dominant clone in primary survives treatment and gains additional mutations at relapse; **b** Pattern 2 (4/24), subclone in primary survives treatment and expands to become dominant clone at relapse; **c** Pattern 3 (17/24), eradication or decrease in frequency of one or more clones in primary and emergence of new clones not previously detected in primary. Left panels, two-dimensional density plots showing clustering of mutations by cancer cell fraction (CCF) in primary and relapse tumours. Darker red areas indicate location of a high posterior probability of a cluster. Clusters are annotated with coding driver mutations and major copy number alterations. Pattern 1: no disappearance of primary clusters on the horizontal axis accompanied by appearance of new clusters on the vertical axis. Pattern 2: existence of cluster positioned on the vertical top and horizontal centre. Pattern 3: disappearance of clusters on the horizontal axis accompanied by appearance of clusters on the vertical axis. Central panels, chromosomal copy-number profiles of primary (upper) and relapse (lower) tumours. Thick and thin lines represent clonal and sub-clonal copy number states respectively. Yellow and dark blue lines denote total and minor copy number alleles. Right panels, Muller plots of evolutionary trajectories. P primary, R relapse. WGD Whole genome duplication.

Three patterns of clonal evolution were apparent at relapse (Fig. 3). In Pattern 1 (3/24 patients), the dominant clone in primary survives treatment and gains additional mutations at relapse (Fig. 3a, Supplementary Fig. 22a). Tumours with Pattern 1 are characterised with no change in clonal composition of the dominant clones, suggesting that they were potentially unaffected by treatment. Pattern 2 (4/24 patients) is featured by subclonal expansion whereby a subclone in the primary survives treatment, and expands to become the dominant clone at relapse (Fig. 3b and Supplementary Fig. 22b). Tumours with Pattern 2 are also accompanied with “branching evolution” feature, where new clones emerge while others are lost. We suspect these clones might have mutations (e.g., *TET2* and 6q deletion) giving them survival and selective advantage. Pattern 3 (17/24 patients) is characterised by the emergence of new clones at relapse, accompanied by the disappearance or decline of primary clones (Fig. 3c and Supplementary Fig. 22c). The three patterns of clonal evolution were not associated with therapy strategies (intensive versus nonintensive pathways) or molecular karyotypes (Fisher’s exact test). It was, however, of note that time to relapse was shorter with Pattern 2 (median 11.6 versus 19.3 months, *P* = 0.019, Wilcoxon rank-sum test).

## Discussion

Using high-depth WGS, we provide for an enhanced genetic model of the development and progression of MM. Our study expands upon previous findings, which have been based on WES/targeted sequencing^3,4,36,46,48,49^, low coverage WGS^50^, or fluorescence in situ hybridization and/or array technology^46,51^. While we have restricted our analysis to MM with an initiating translocation, our findings provide evidence for a common origin of tumour subpopulations with many tumours being composed of at least one subclone, reflecting the clonal heterogeneity present in both primary and relapse.

In addition to known coding drivers, we extend the number of potential non-coding drivers in MM, including those associated with *CXCR4* and *BIRC3*. Somatic mutations in *BCL6* promoters are common in MM^52^; however, since the gene is a common target of normal activation-induced deaminase (AID) in the germinal centre^53^, the relevance of these promoter mutations to MM biology is questionable. Noncoding regulatory regions additionally disrupted at relapse, included those targeting *XBP1*, *RBX1*, and *SCML1*. Common pathways affected by coding and noncoding mutations arising in MM relapse included those associated with WNT-signalling, MAPK-signalling, and NOTCH-signalling, base excision repair, cell cycle, telomere maintenance, and cellular senescence (Table 2). Notably, relapse was characterised by frequent additional CNAs, the most common being 17p deletion. Since the additional CNAs often occurred at unstable genomic regions such as 11q and 14q, it suggests increased chromosome instability are important means to escape therapy, analogous to that seen with chronic myeloid leukaemia in response to imatinib^54^. Our findings suggest that 21q gain, 13q deletion, and mutation of *CCND1*, *MAX*, *PRKD2*, *DIS3*, and *NRAS* are early events. The chronology of coding events identified from our study are broadly consistent with previous WES-based analyses^1,55,56^, any discrepancies are likely to be a consequence of sample size, representation of MM subtype, and number of coding drivers considered.

**Table 2:** Summary of relapse-specific coding driver mutations, promoter mutations, CRE mutations, driver translocations, and large-scale genomic changes identified in 24 primary tumour-relapse pairs grouped by subtype.

Subtype	Coding drivers	Promoters	CREs	Driver translocations	Frequent large-scale genomic changes
*t*(4;14)	*KRAS; TP53; FGFR3; FAM46C; TRAF2; NF1; XBP1*	*MTFRL1; FLT3LG; IL12A; POLG; XBP1; B3GALNT1; ALG10B*	*ABCA10; ABCA5*	*MAP3K14* t(17,14)(q21,q32)	17p deletion Further copy number changes at unstable genomic regions (11q and 14q) Increased telomere length
*t*(11;14)	*PRDM1; LTB; IDH2; KRAS; NRAS; CCND1; ATM; FAM154B; MLL3*	*RBX1; FAM81A; POLG; KCTD13; SCML1*	*SCAF8*		
*t*(14;16)	*NRAS; TET2*	*MYO1E; ALG10B; TMSB4X; KCTD13; SCML1*			

*CRE* *cis*-regulatory element.

Overall, the mutational load was higher in relapse MM and aberrations previously linked to MM resurfaced in both primary pretreatment and relapse tumours in our cohort, including mutations in *RAS* genes, *DIS3*, *TP53*, *FGFR3*, and *PAX5* CRE mutations. As well as highlighting mutation of genes with established roles in MM, we identified a number of frequently acquired de novo coding mutations (e.g., *FAM46C*, *TRAF2*, *NF1*, and *XBP1*), de novo translocation (*MAP3K14*) and pre-existing mutations (e.g., *TET2*). Longer telomeres at relapse could be associated with treatment as observed in chronic myeloid leukemia^57^. Therapy targeting telomerase/telomeres should be further explored in MM as lengthened telomeres may provide a mechanism for treatment resistance^58^.

By performing high-depth WGS, we have been able to better refine the patterns of genomic evolution at relapse in MM compared to previous studies^3,4^. Notably, the “branching evolution” and “differential clonal response” models described by Bolli et al.^3^ often co-occurred as one single model (Pattern 2) in our analysis. Additionally, we did not find evidence for an association between *t*(11;14) MM with a “no change/linear” model^3^. The study by Jones et al. which included a small number of overlapping cases failed to identify Pattern 2 whereby a subclone survives treatment and expands at relapse^4^. Insights into tumour evolution has the potential to inform clinical decisions^59^. “Evolutionary herding”, in which clonal composition of tumours is tunnelled by a treatment to increase their sensitivity to another treatment, has been proposed as a strategy to combat treatment-resistance in tumours^60^. Despite a limited number of samples, we found little evidence that the evolutionary trajectory of MM is solely dictated by molecular karyotype or significantly influenced by current therapeutic strategies, questioning the viability of “evolutionary herding” in controlling drug resistance in MM. It was however noteworthy that Pattern 2 was associated with significant shorter time to relapse. Going forward, further strategies should be explored to accurately predict tumour dynamics and tailor patient therapy^61^.

Higher proportion of C•G>G•C at relapse is associated with DNA damage by oxidative stresses^62^, possibly due to oncogene activation and/or enhanced metabolism in relapsed MM^63^. AID/APOBEC activity contributes to increased mutational burden and associated with shorter time to relapse. APOBEC mutagenesis has been shown to promote survival and therapy escape in cancer through driving subclonal diversity, immune evasion, and genomic instability^64^. Collectively, these data suggest APOBEC family enzymes as potential therapeutic targets for treatment-resistance MM.

Inevitably, due to technical limitations, our ability to detect mutations in rare cells (mostly related to currently achievable levels of coverage with WGS) and spatial sampling constraints, our models potentially underestimate clonal heterogeneity in MM. We did however observe the loss of primary tumour clones at relapse in 21 of 24 cases, suggesting that some subclones are eradicated by therapy (Supplementary Fig. 22). Nevertheless, treatment failed to eradicate the founding clone in all cases. Our data also imply the acquisition of new mutations, which subsequently undergo selection and clonal expansion, potentially contributing to disease progression. It is likely that some mutations gained at relapse may alter the growth properties of MM cells, or confer resistance to additional chemotherapy.

Presently strategies to improve the poor cure rates of relapsing MM are limited. The forces shaping the evolutionary trajectory of MM have relevance to informing patient management. Williams et al. proposed that following a “big bang”, neutral evolution is a major feature of many cancers^65^. Application of same model to MM exome sequencing data suggested that neutral evolution is also a significant feature of MM^66^. Serious criticism has however been levelled at the assumptions on which the Williams et al. model is predicated^67–70^. In the light of such critique, as well as findings from our current WGS analysis and MM sequencing studies performed by other researchers^71^, it is apposite to reappraise the role of neutral evolution in MM. It seems highly unlikely neutral evolution is a dominant evolutionary force in MM and its evolutionary trajectory is essentially Darwinian-shaped by selection and subsequent expansion of diverse clones in patients.

MM cells routinely acquire a small number of additional mutations at relapse, and some of these mutations may contribute to clonal selection and therapy resistance. While mutations in *CRBN* and associated genes have been implicated as a mechanism of acquired drug resistance to IMiDs, our analysis suggests mutation per se is unlikely to be a universal basis of acquired IMiD resistance. This does not preclude epigenetic alterations, which are a feature of relapse influencing drug transport, escape from apoptosis, and dysregulated intracellular signalling pathways, all of which can contribute to resistance^72^.

Here, we have demonstrated that relapsed MM harbour significantly more mutations than primary tumours and clonal selection of mutations occurs at relapse, which are accompanied by subclonal heterogeneity. Theoretically, these data provide a rationale for identifying disease-causing mutations for MM, which may be amenable to targeted therapies to avoid the use of cytotoxic drugs, many of which are mutagens. However, it remains to be determined whether the current arsenal of therapies directed against downstream effectors of mutated genes will be effective given that the MM genome in an individual patient is likely to be continuously evolving. It is conceivable that in the near future, chemotherapy-based regimens may be relegated to fifth or sixth line treatment after patients have failed proteasome inhibitors, IMiDs and/or immunotherapy. Although speculative, however successful immunotherapy will be in an individual patient, Darwinian evolution of MM would imply that such therapy is unlikely to affect cure. It is therefore likely that eradication of the founding clone, as well as all of its subclones, will be required to effect complete cure.

## Supplementary information

Supplementary Figures

Supplementary Tables

## Data Availability

Raw promoter capture Hi-C data for naïve B-cells were obtained from European Genome-Phenome Archive (EGA; accession code EGAS00001001911). Replication timing data for B-lymphocytes was downloaded from Replication Domain Database^34^. Raw WGS data generated as part of this study can be accessed through EGA accession code EGAD00001005491.
