# Supplementary material for: An enhanced genetic model of relapsed IGH-translocated multiple myeloma evolutionary dynamics
Source: Blood Cancer J. 2020 Oct 14;10(10):101. doi: 10.1038/s41408-020-00367-2 (PMC7560599; doi:10.1038/s41408-020-00367-2)
Supplement: Supplementary file 1 — Supplementary Figures [file 41408_2020_367_MOESM1_ESM.pdf]

SUPPLEMENTARY FIGURES

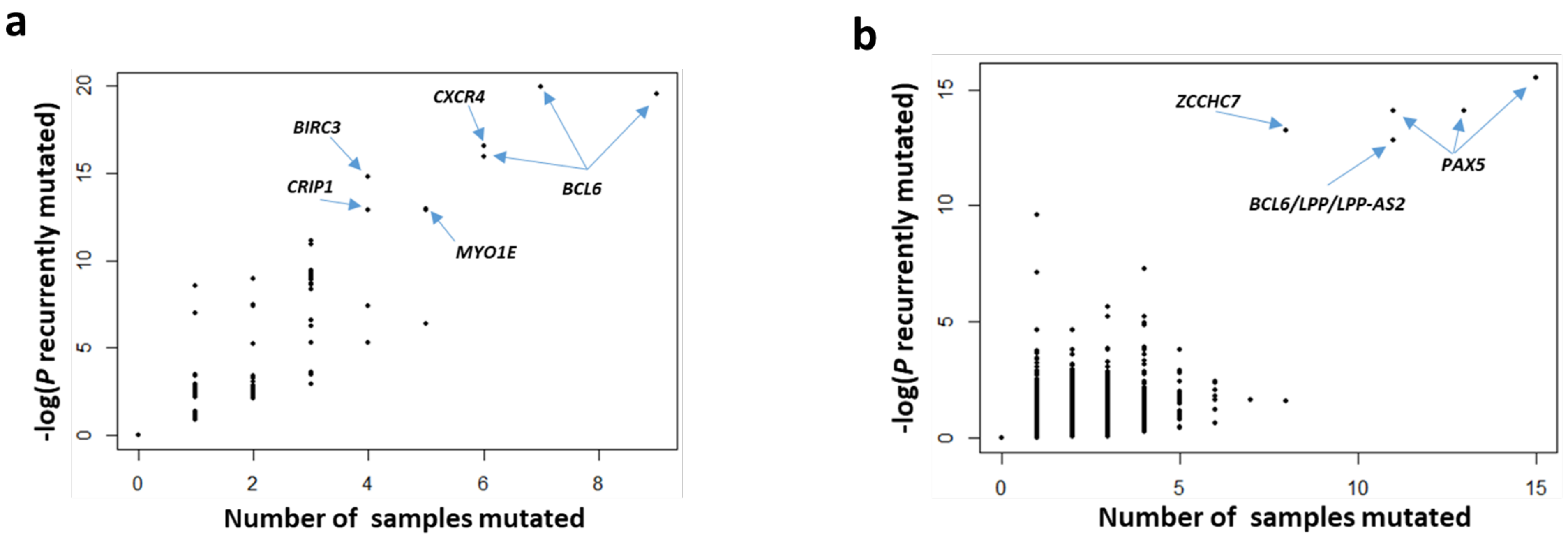

Supplementary Figure 1: Recurrently mutated regulatory elements identified in 80 primary tumours. (a) Recurrently mutated promoters and (b) *cis*-regulatory elements.

t(4;14)

5939

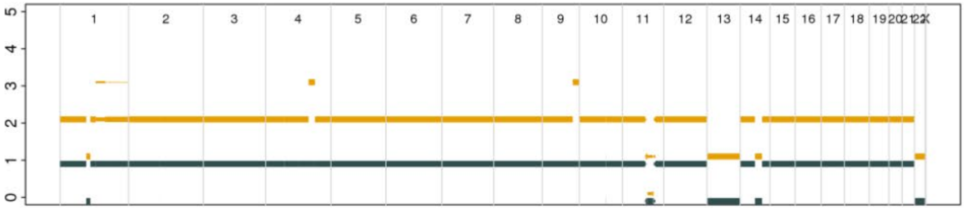

6030

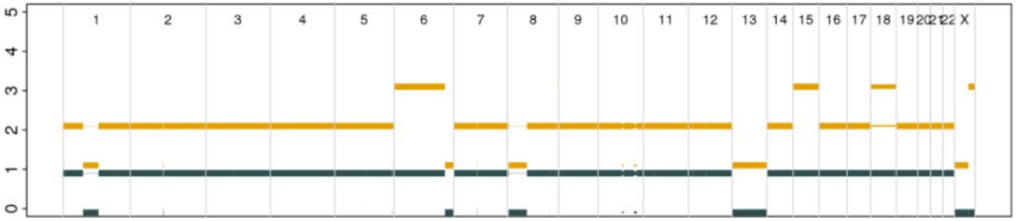

6076

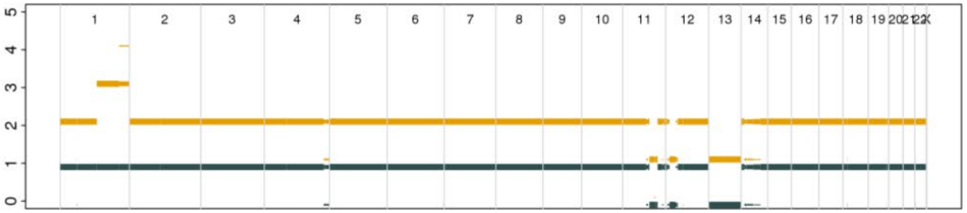

6163

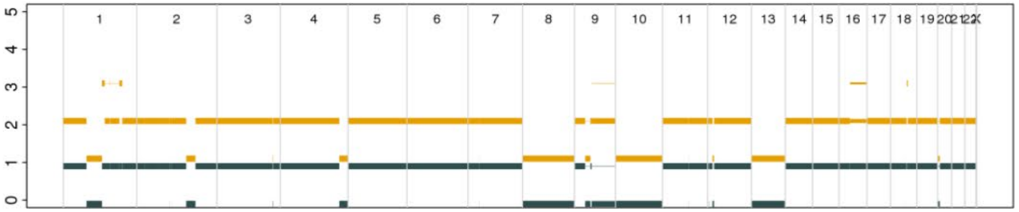

6279

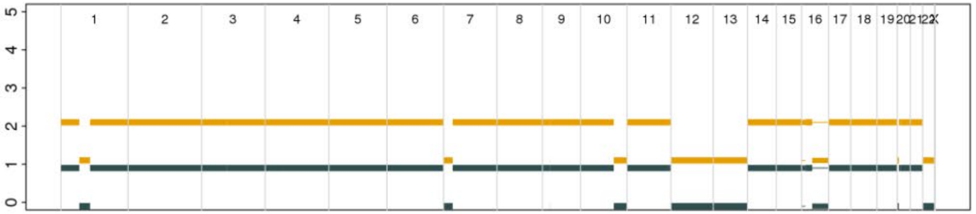

6345

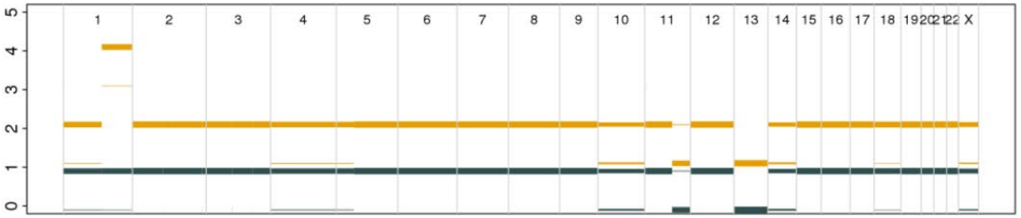

6425

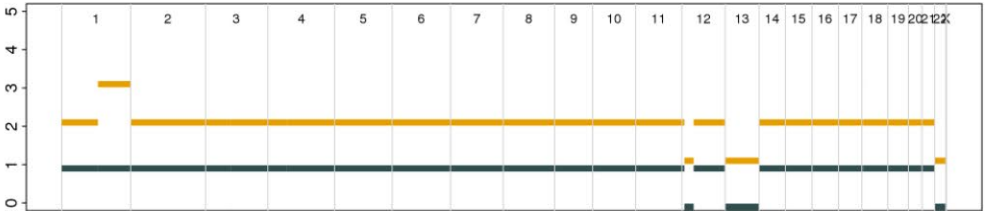

6702

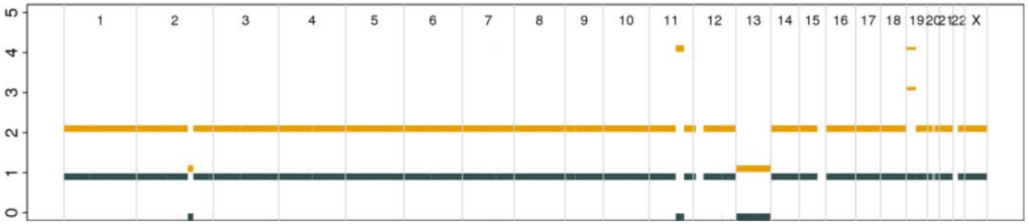

7005

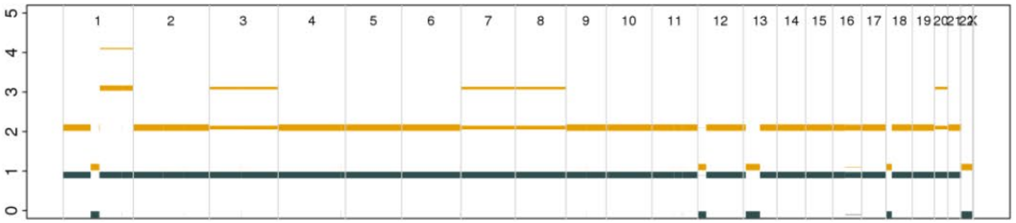

7020

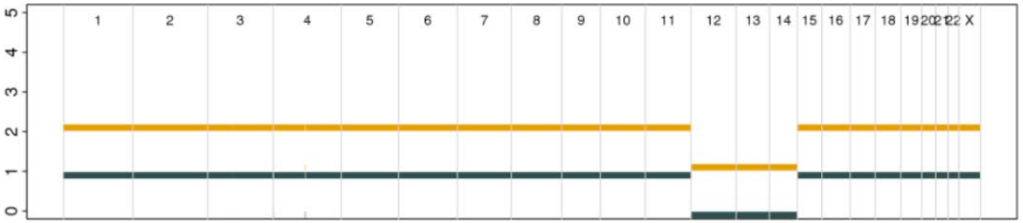

7240

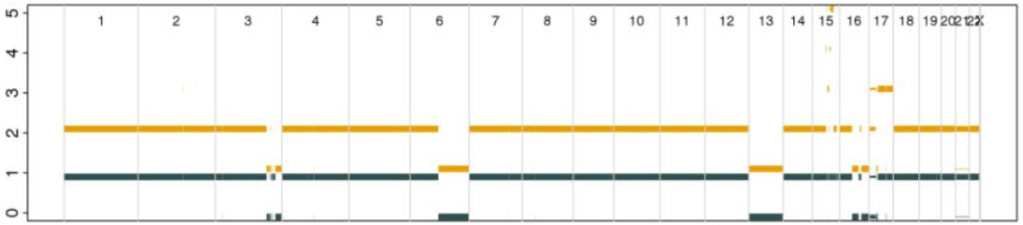

7348

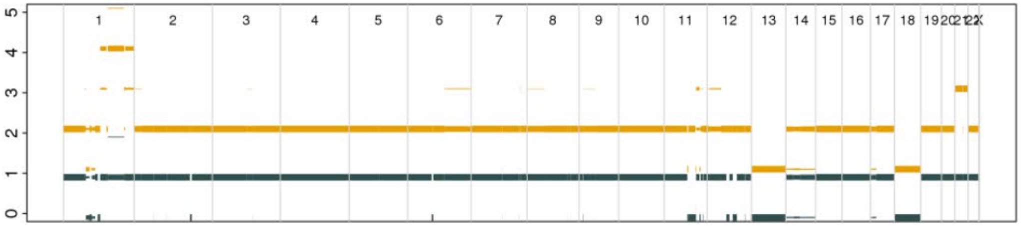

7729

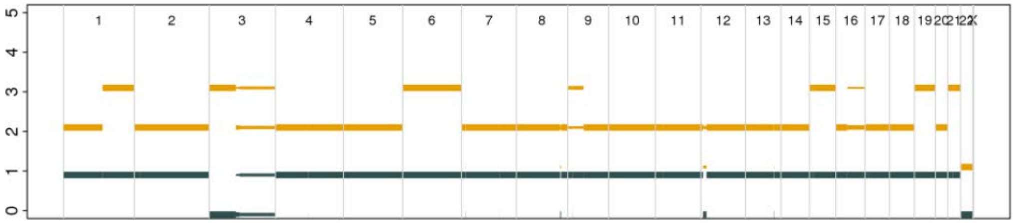

7794

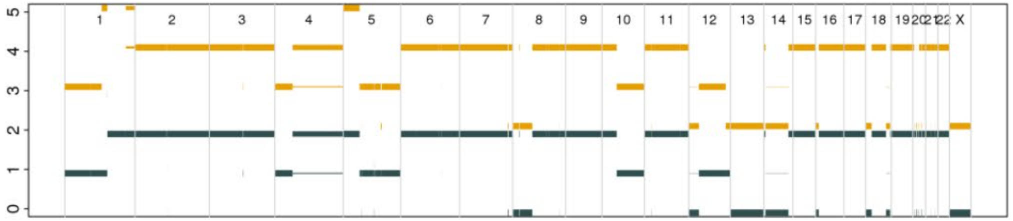

7842

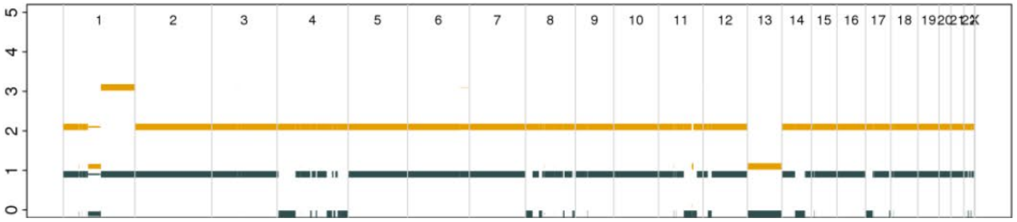

7880

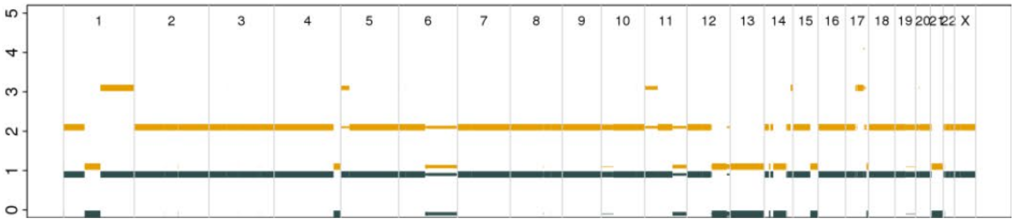

7915

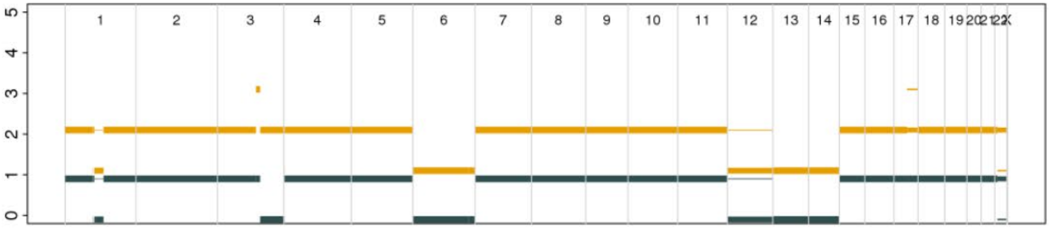

7925

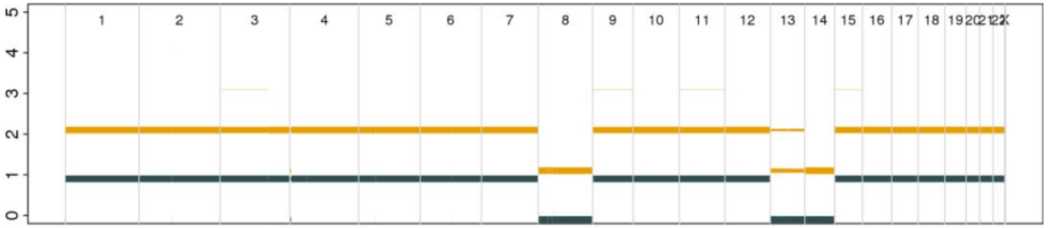

7950

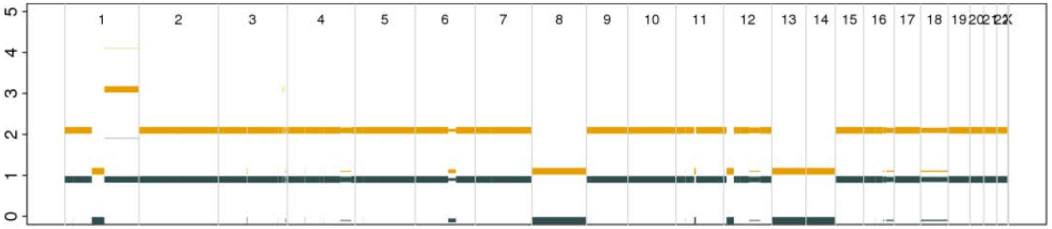

7956

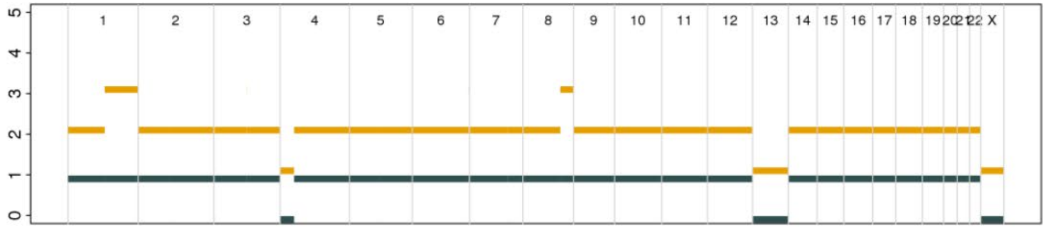

8043

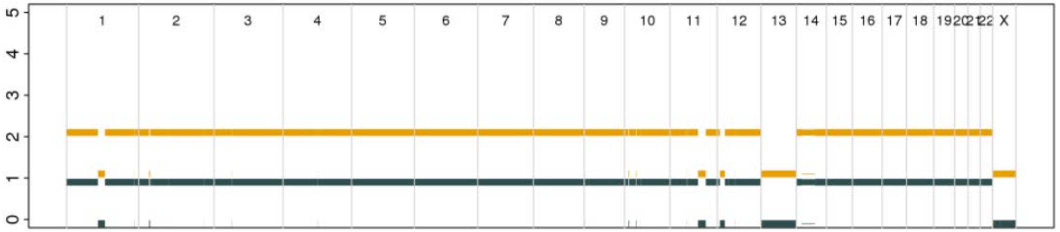

8237

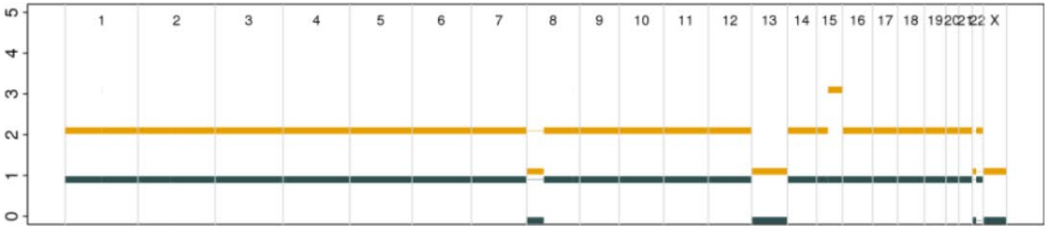

8573

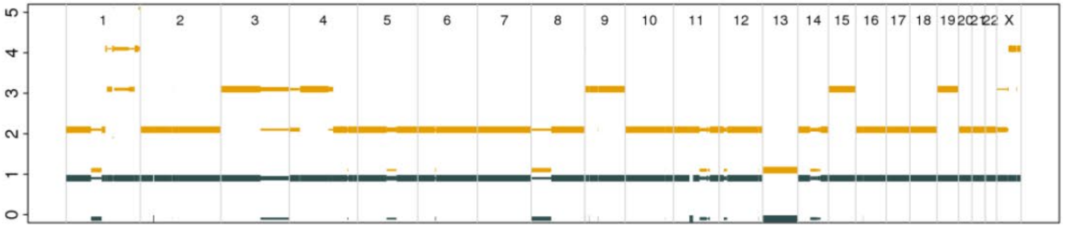

8928

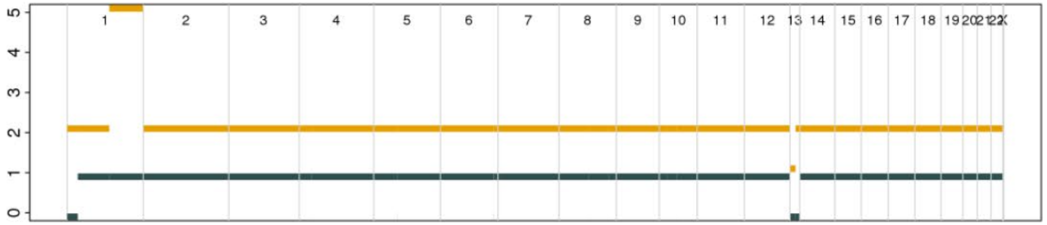

8979

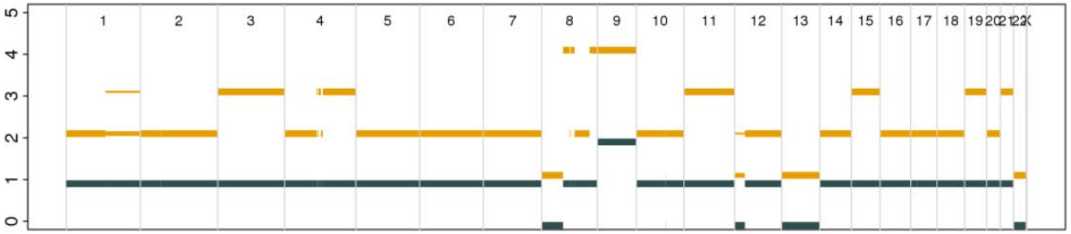

9292

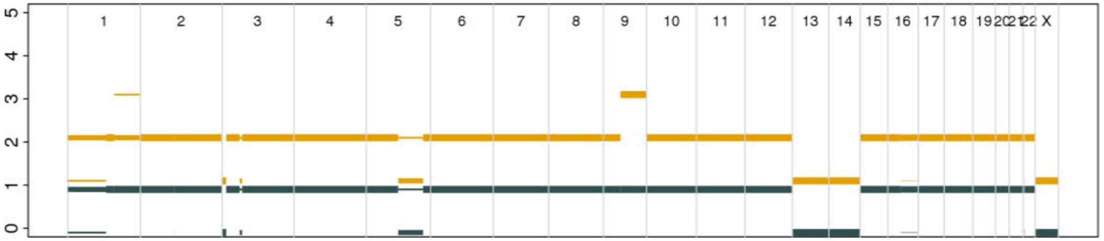

9376

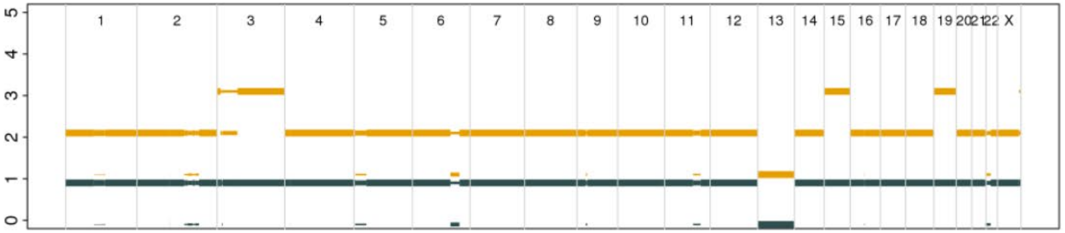

9524

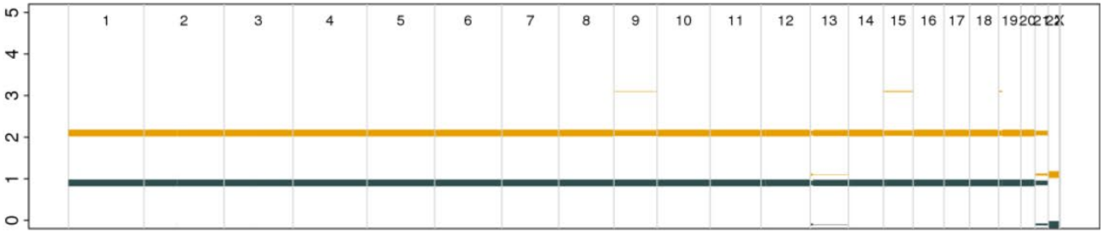

9718

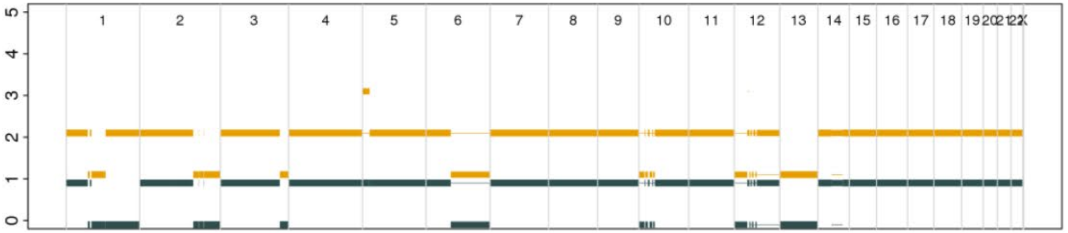

10068

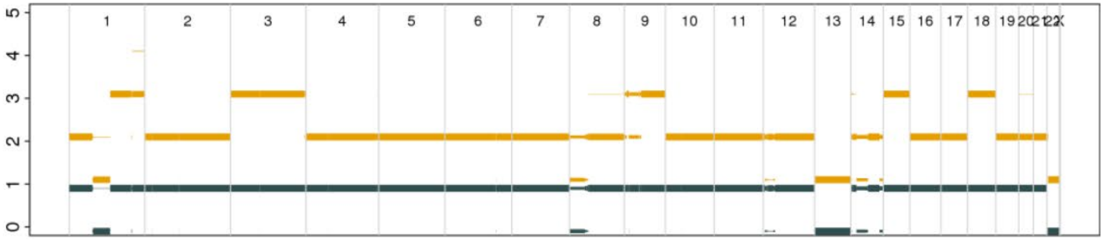

10597

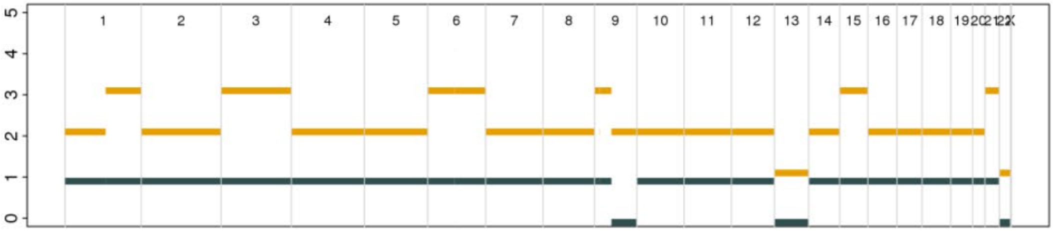

10772

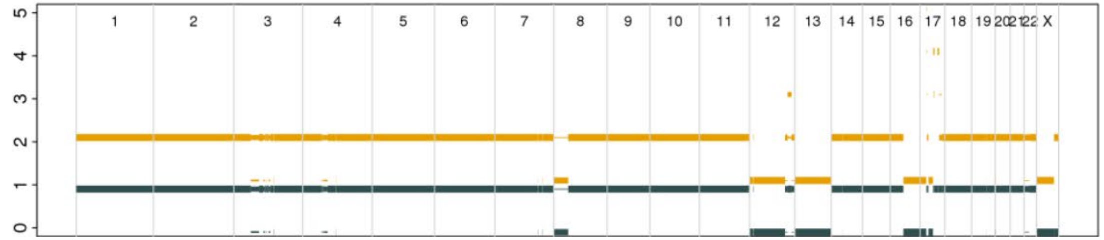

11029

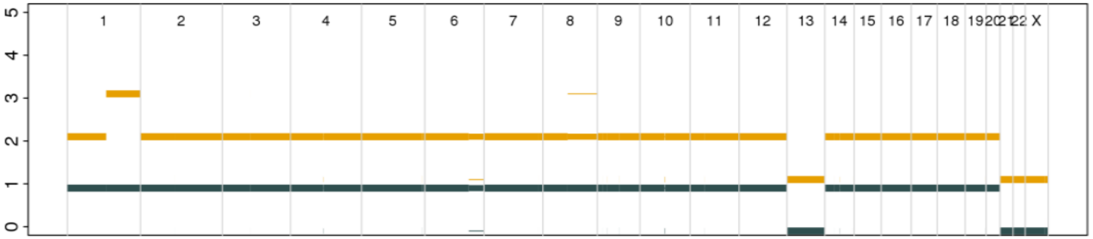

11668

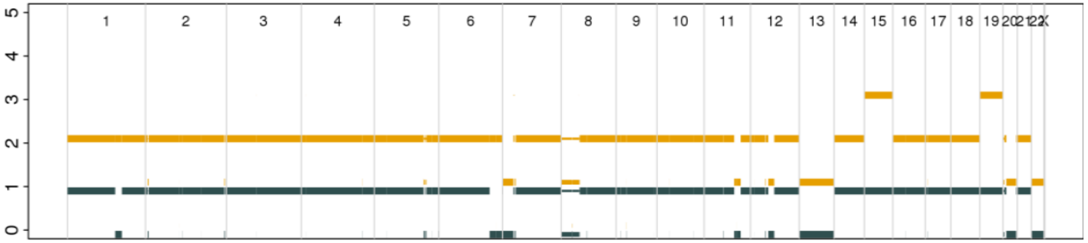

11897

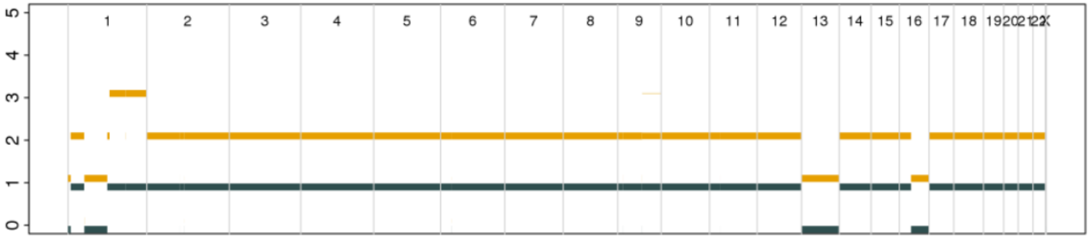

12101

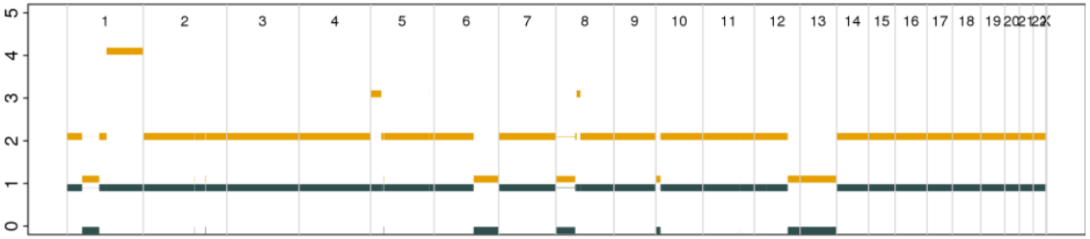

12546

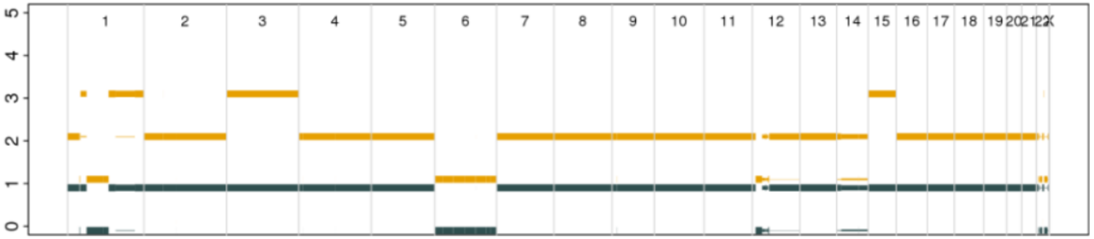

13029

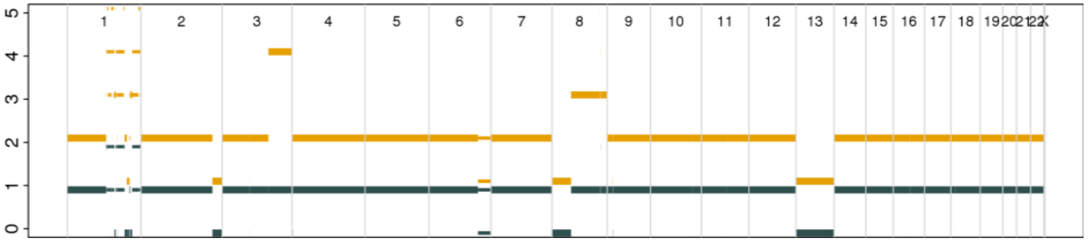

t(11;14)

1305

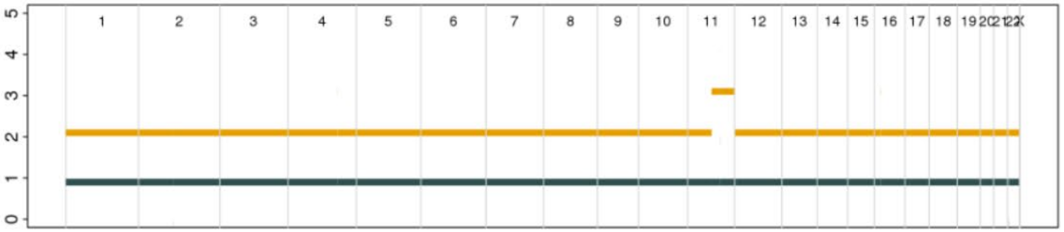

1334

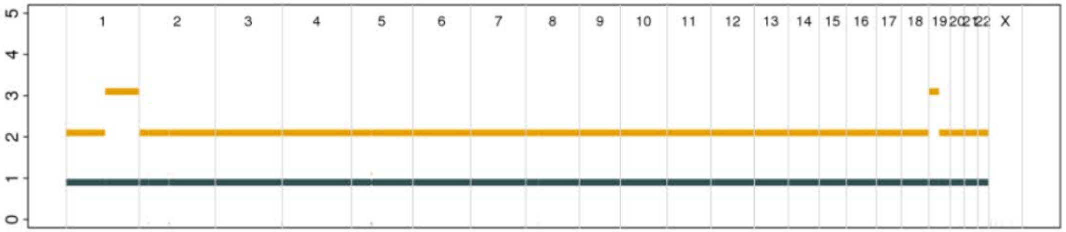

5695

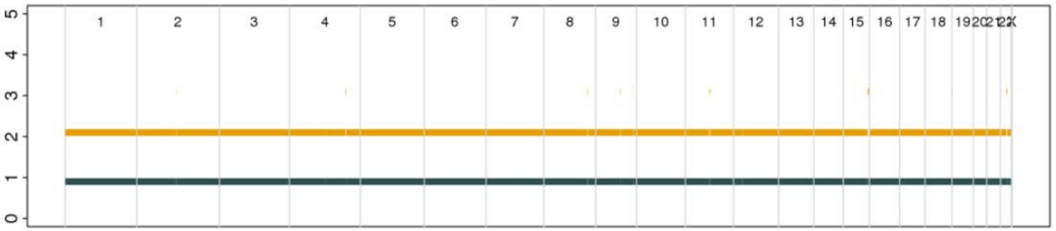

5699

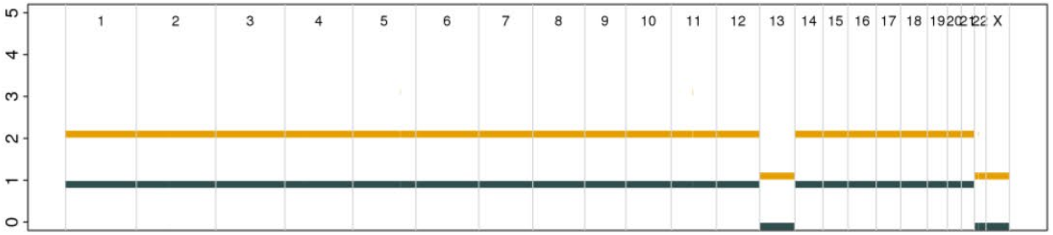

5834

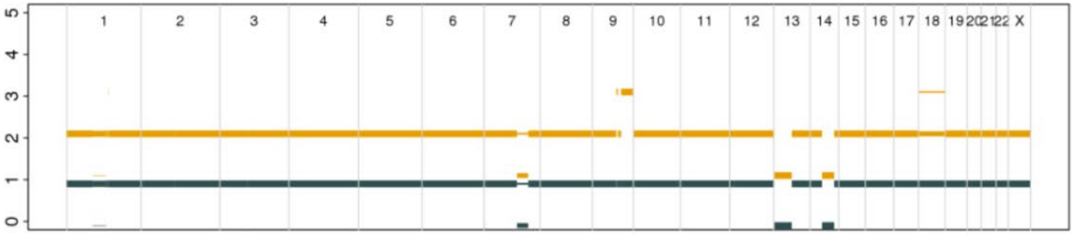

5836

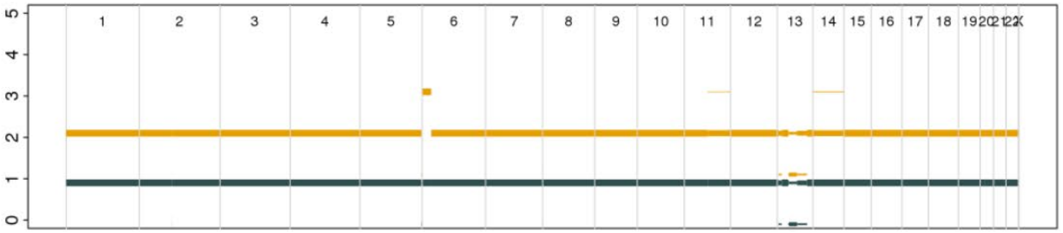

6016

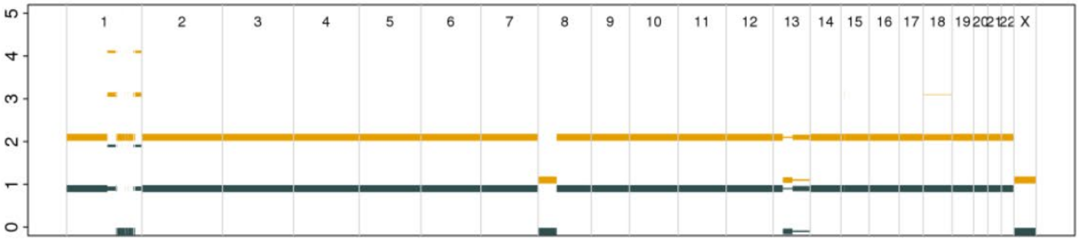

6178

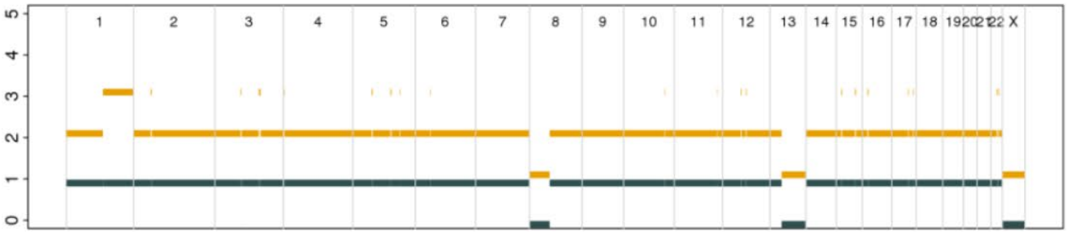

6229

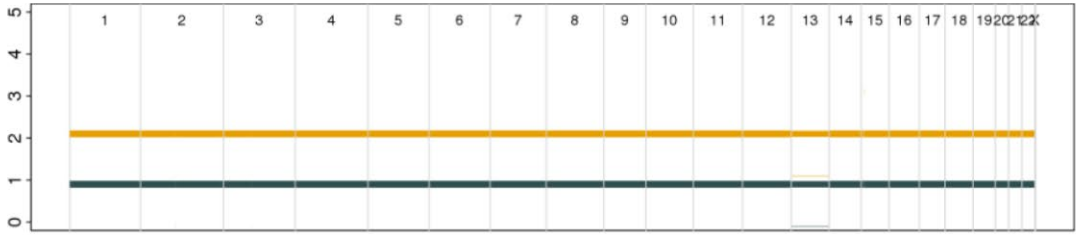

6277

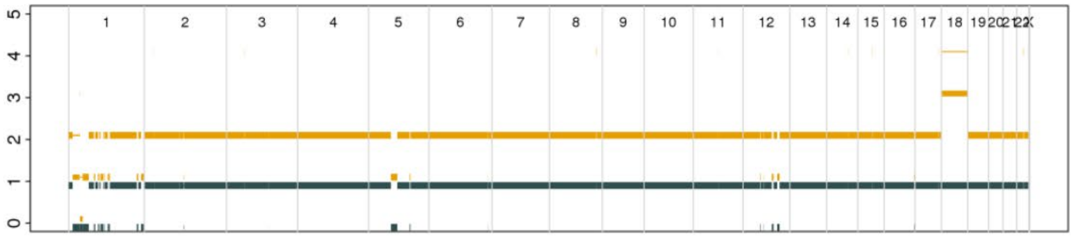

6415

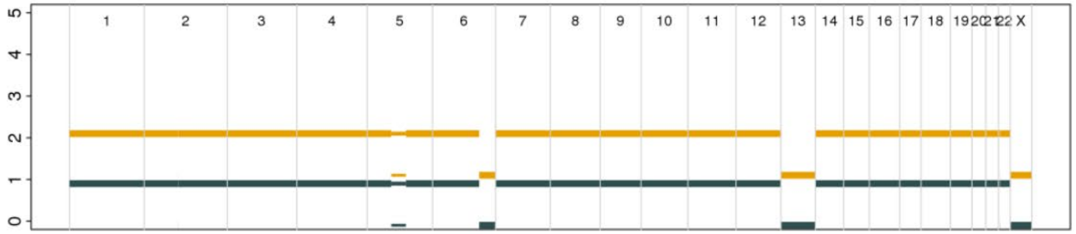

6501

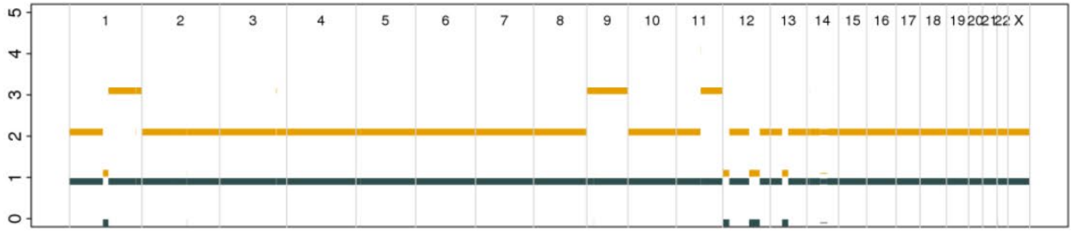

6706

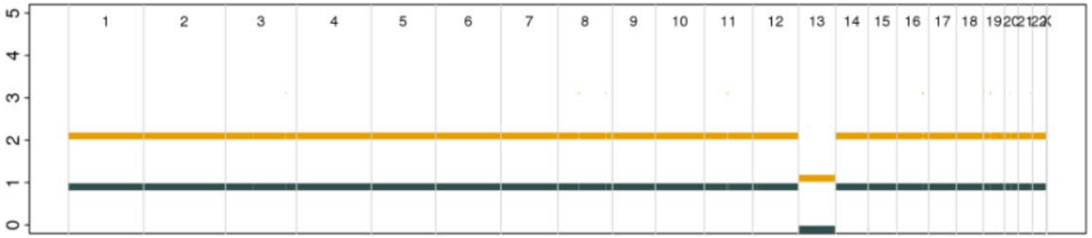

6988

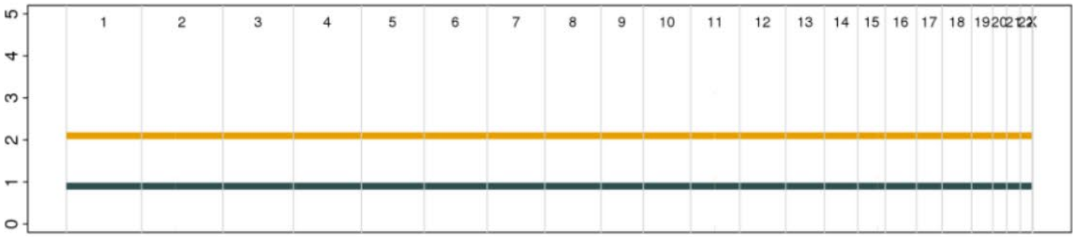

7000

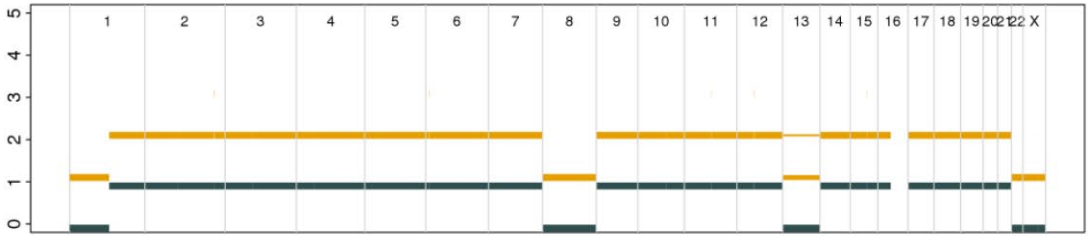

7164

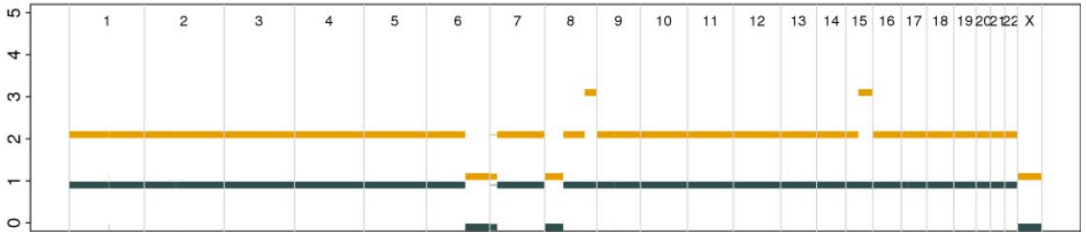

8245

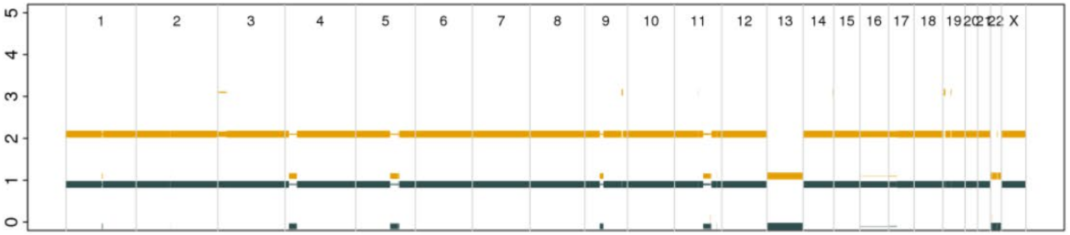

8567

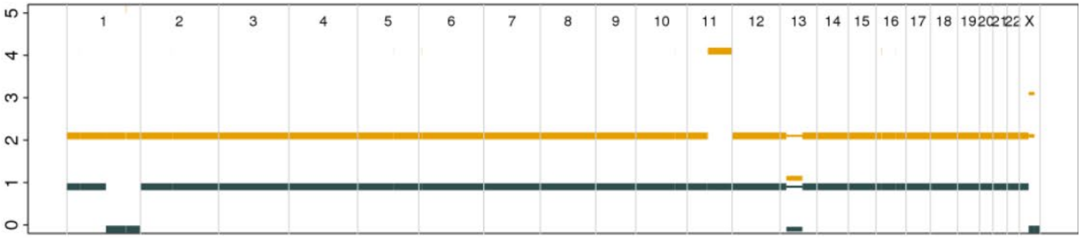

9069

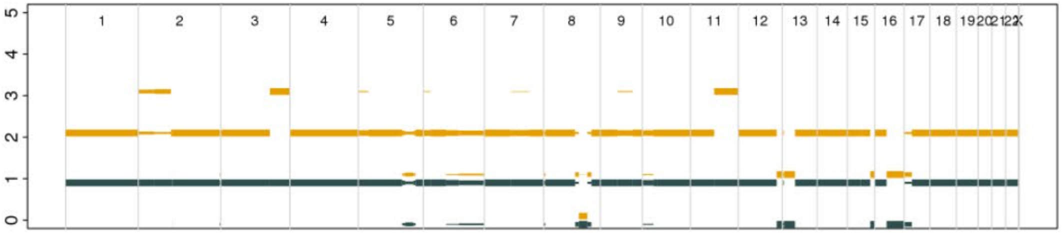

9126

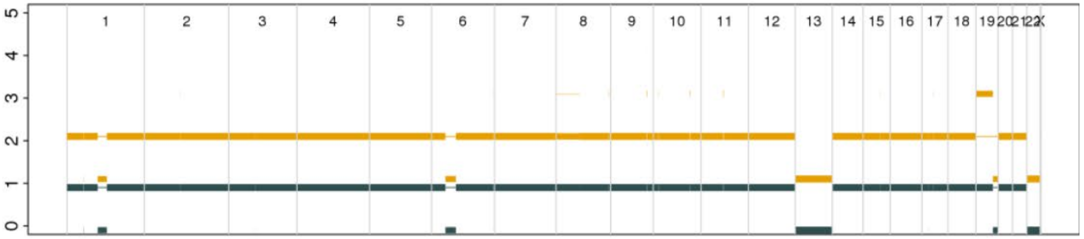

9176

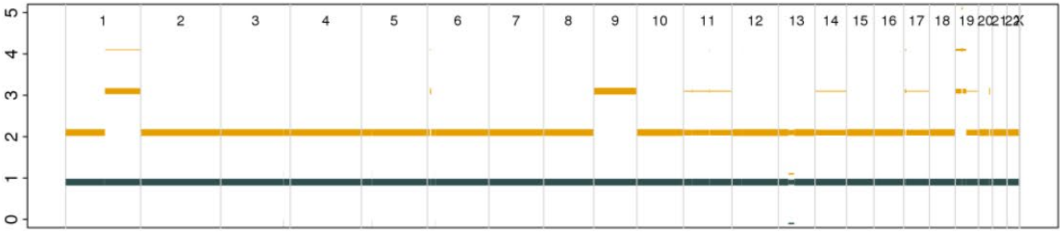

9210

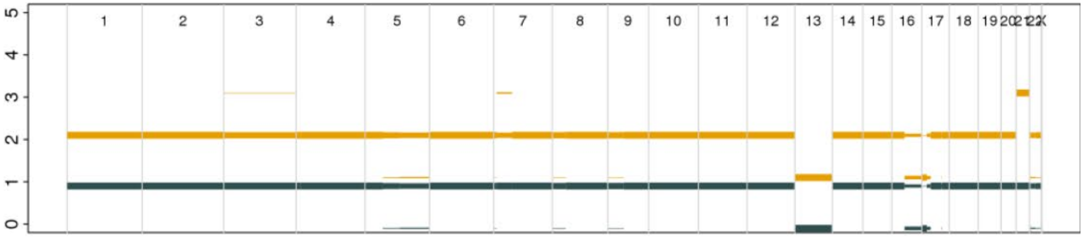

9249

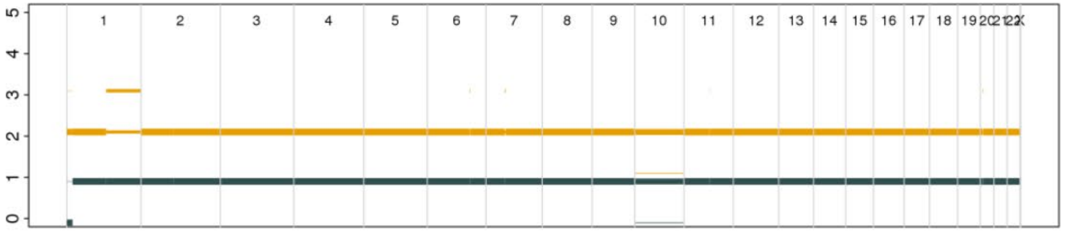

9289

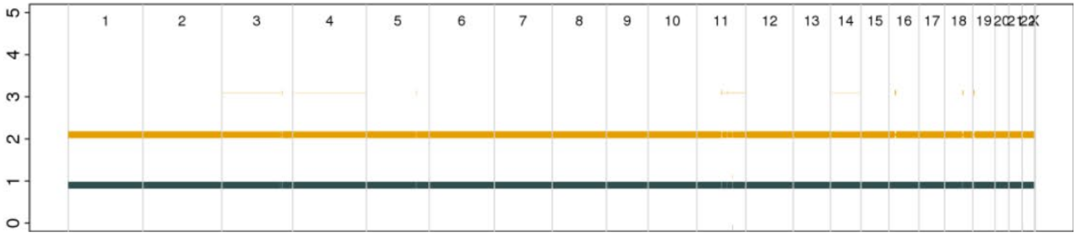

9337

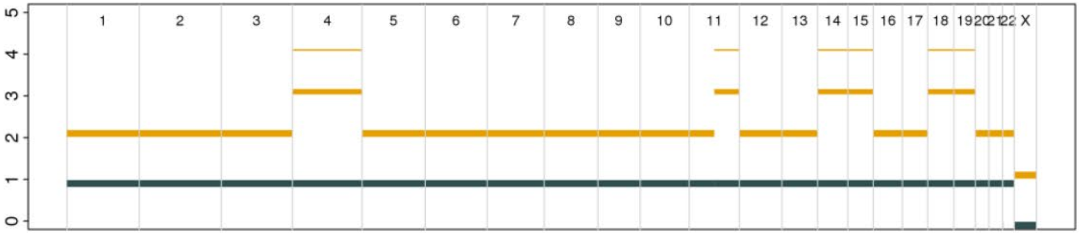

9409

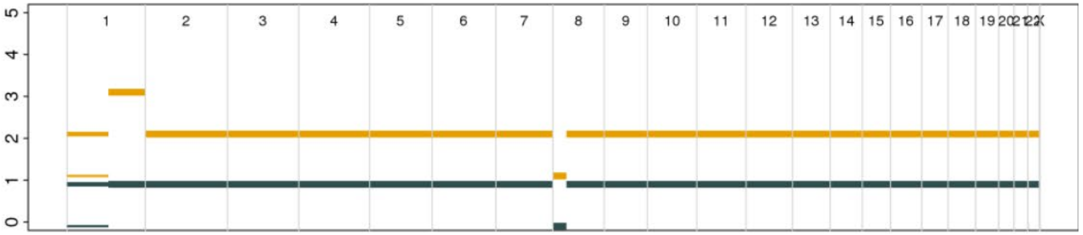

9515

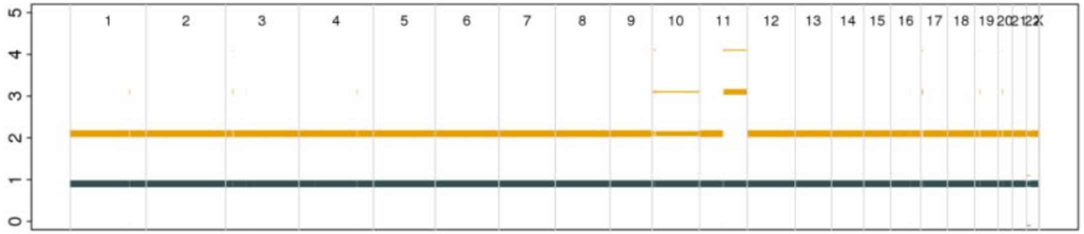

9544

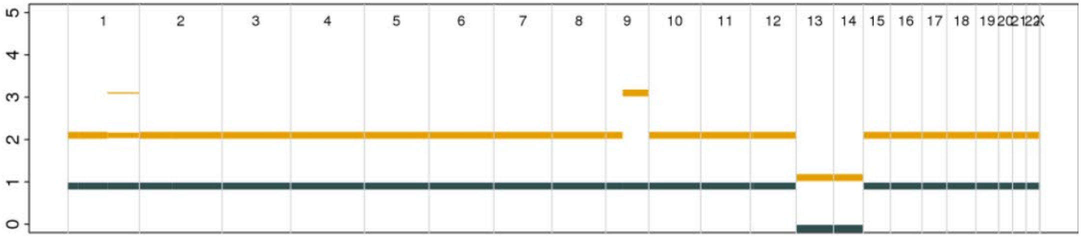

9623

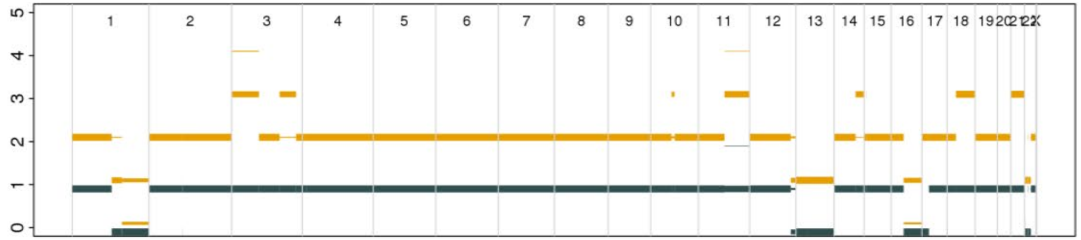

9917

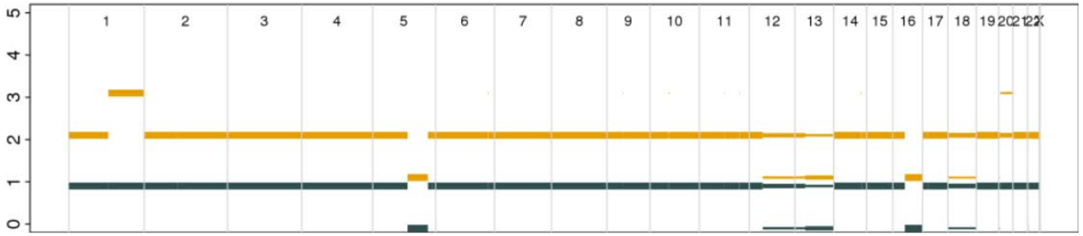

9931

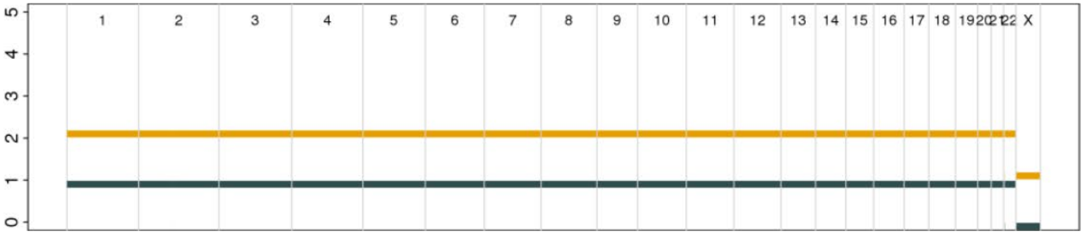

10085

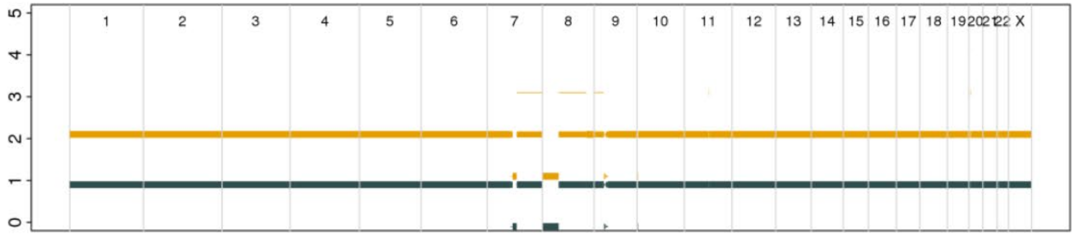

10212

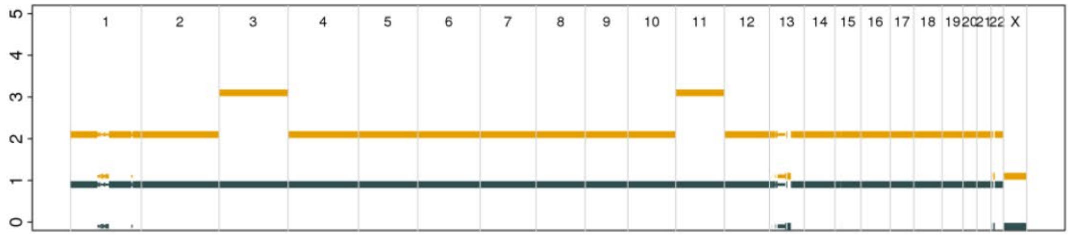

10365

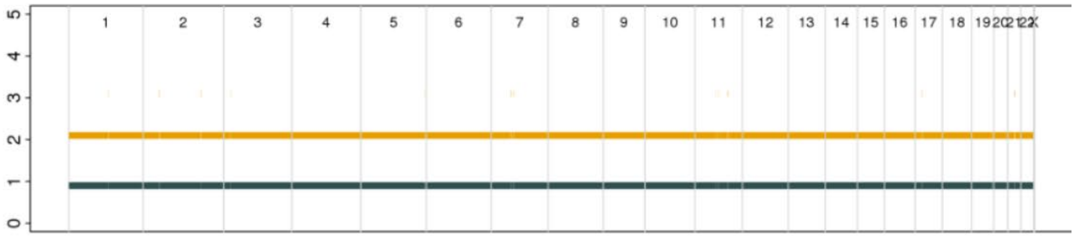

10801

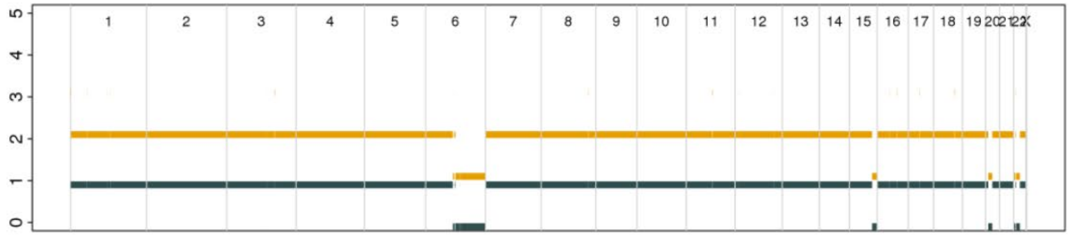

11949

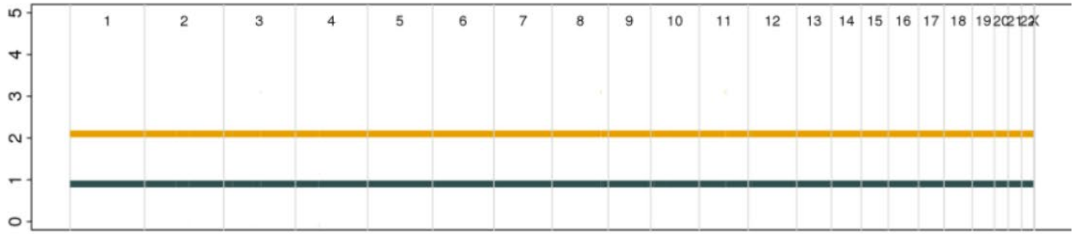

12227

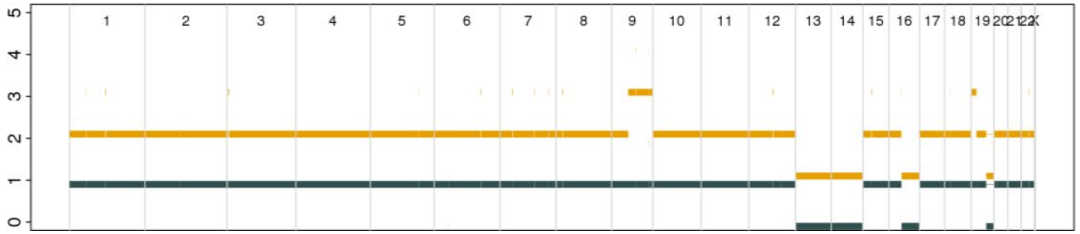

12541

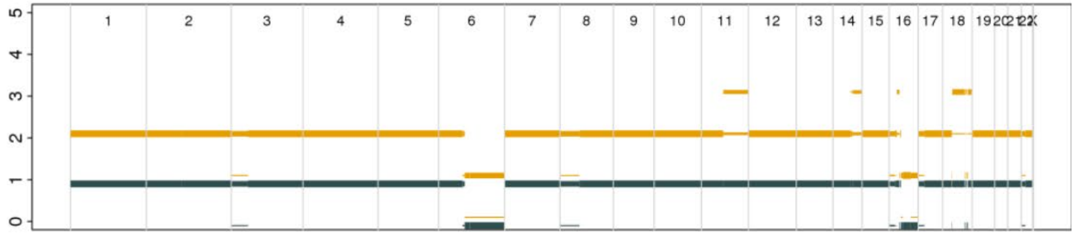

t(14;16)

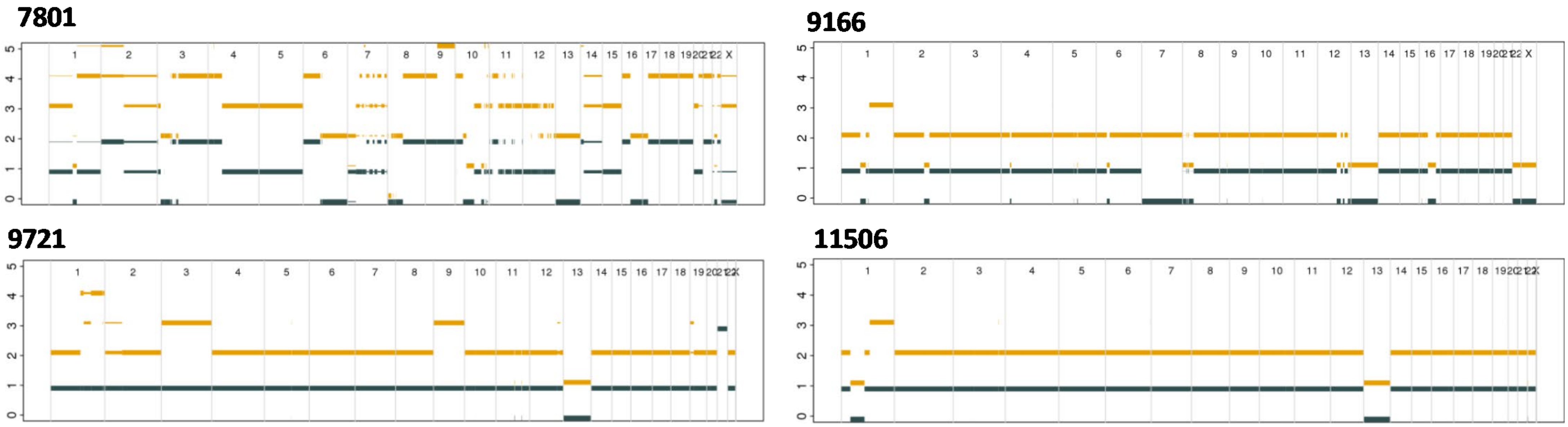

Supplementary Figure 2: Copy number plots for 80 primary tumours. Clonal copy numbers are represented as solid lines with higher intensity than subclonal copy number changes represented as thin line. Yellow: total copy number, dark blue: copy number of the minor allele. Copy number > 5 is not shown.

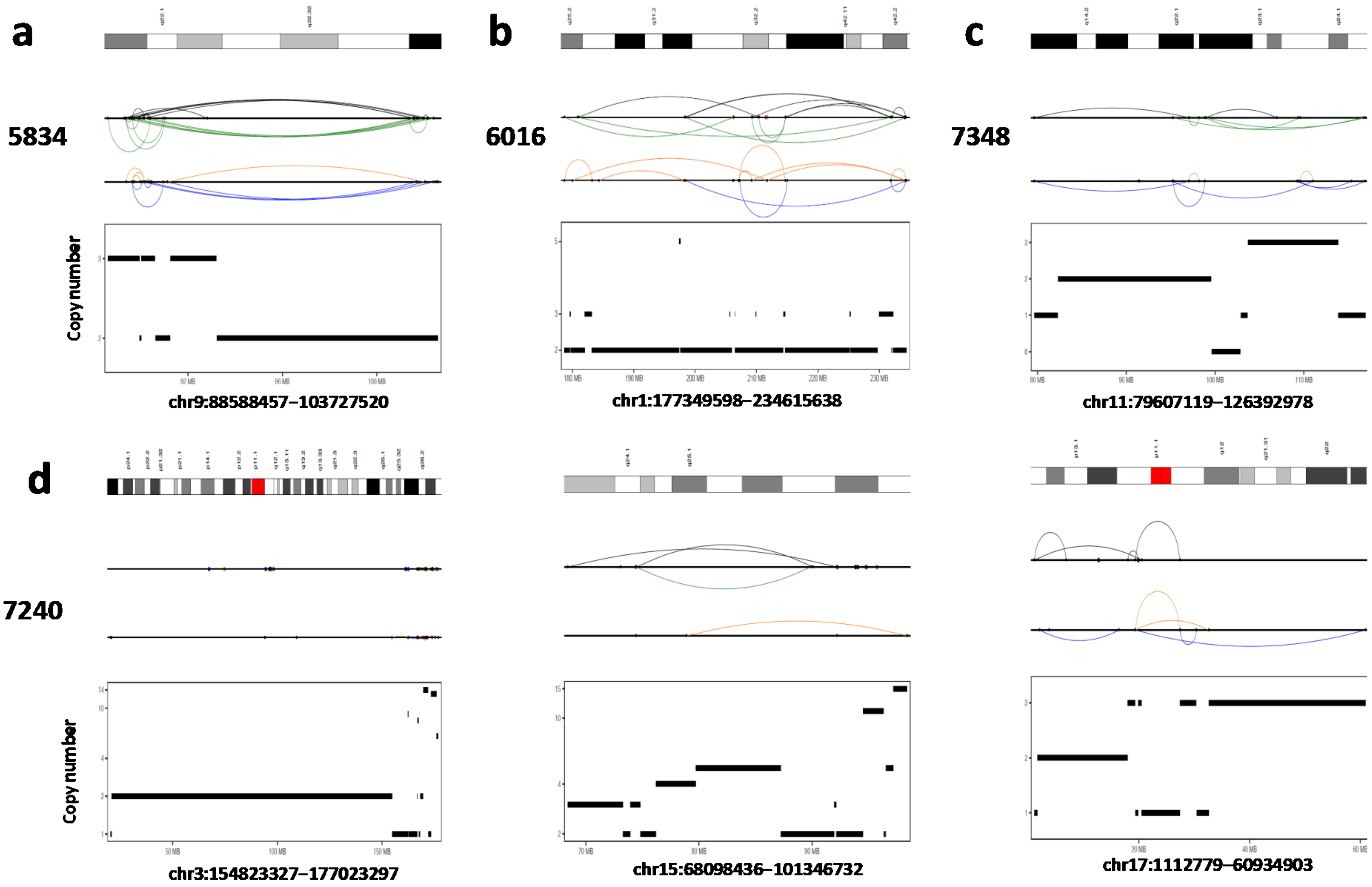
a
5834
Copy number
chr9:88588457–103727520
b
6016
chr1:177349598–234615638
c
7348
chr11:79607119–126392978
d
7240
Copy number
chr3:154823327–177023297
chr15:68098436–101346732
chr17:1112779–60934903

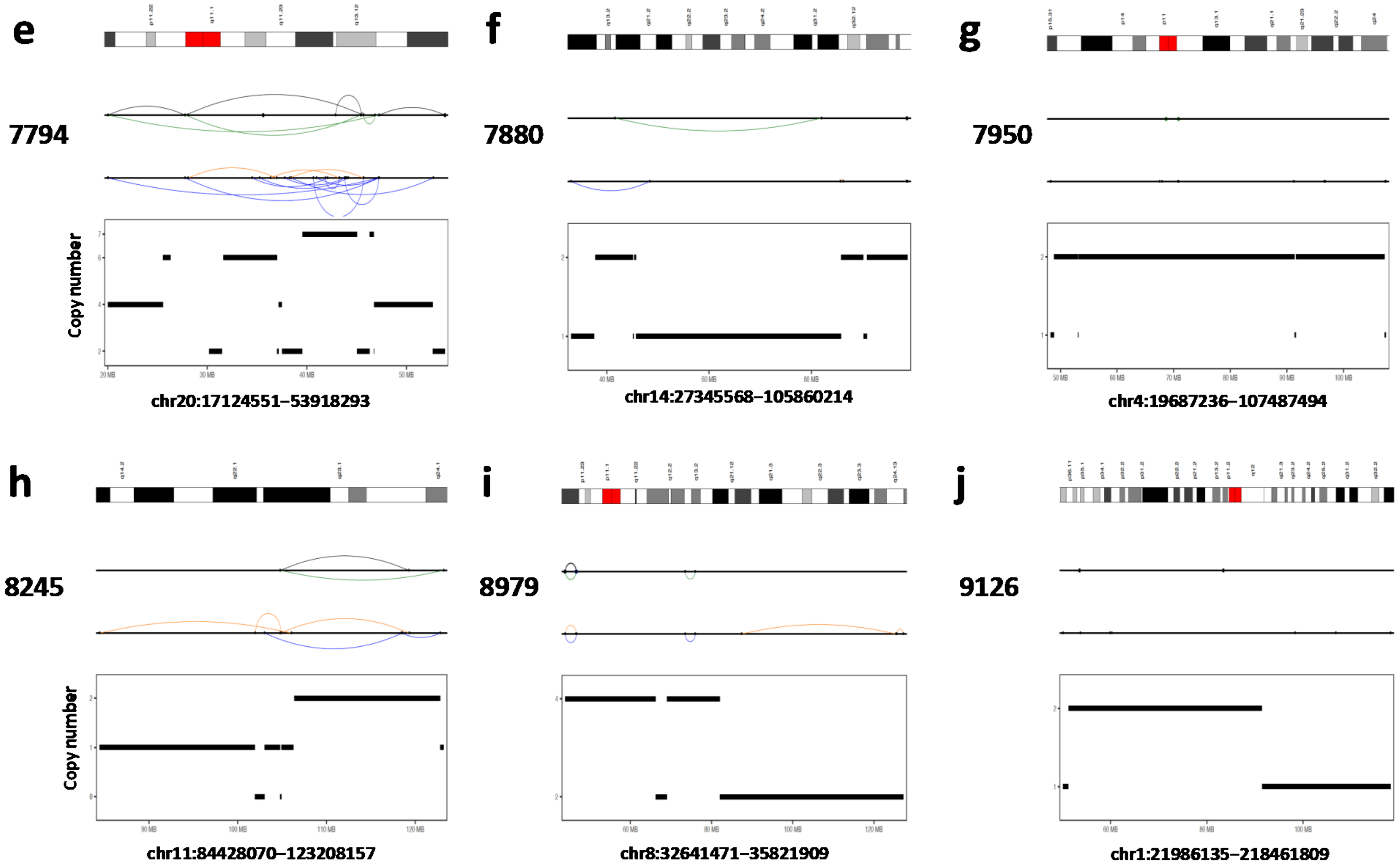
e
7794
Copy number
chr20:17124551–53918293
f
7880
chr14:27345568–105860214
g
7950
chr4:19687236–107487494
h
8245
Copy number
chr11:84428070–123208157
i
8979
chr8:32641471–35821909
j
9126
chr1:21986135–218461809

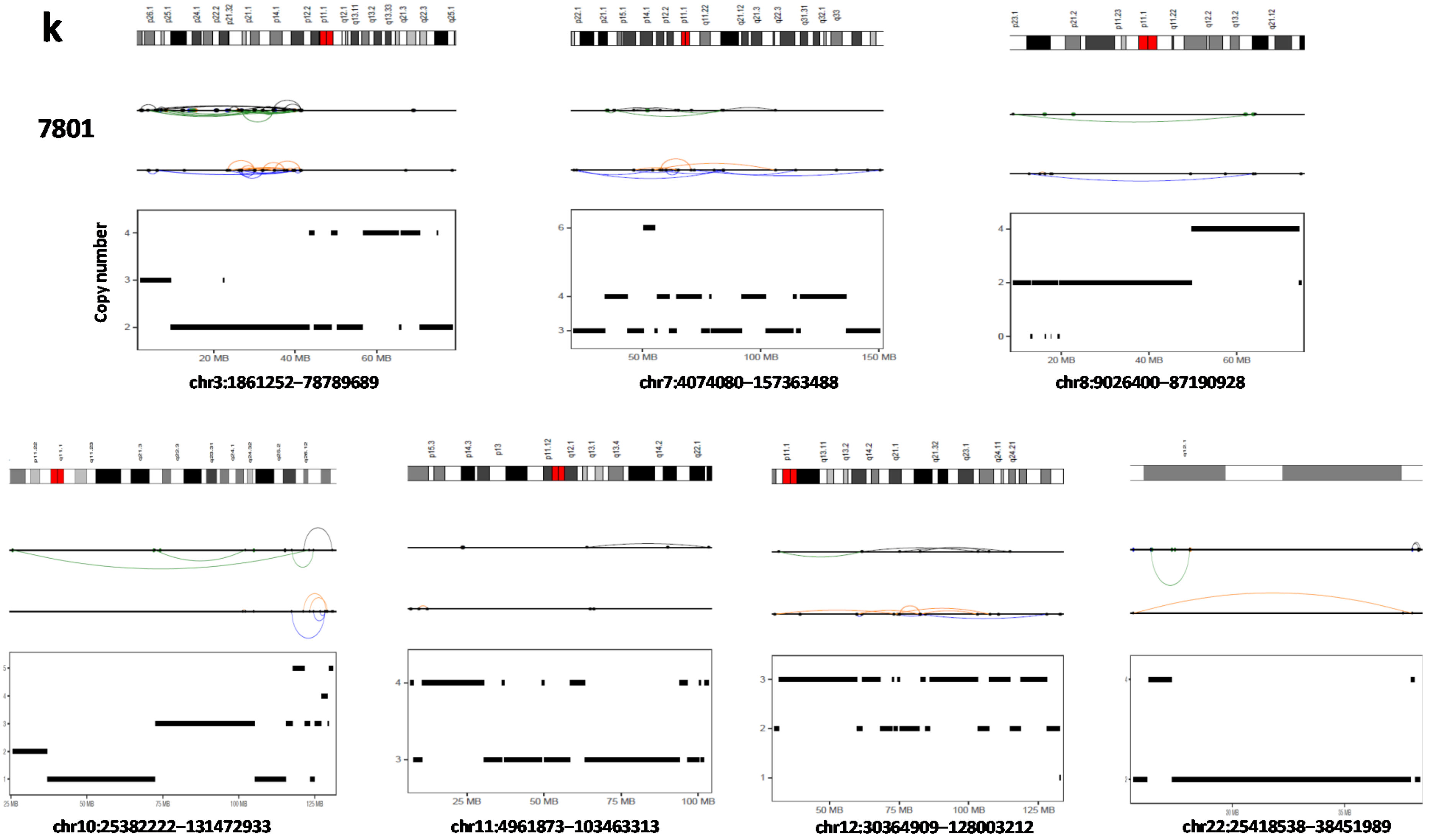
k
7801
Copy number
20 MB
40 MB
60 MB
chr3:1861252–78789689
50 MB
100 MB
150 MB
chr7:4074080–157363488
20 MB
40 MB
60 MB
chr8:9026400–87190928
chr10:25382222–131472933
25 MB
50 MB
75 MB
100 MB
chr11:4961873–103463313
50 MB
75 MB
100 MB
125 MB
chr12:30364909–128003212
chr22:25418538–38451989

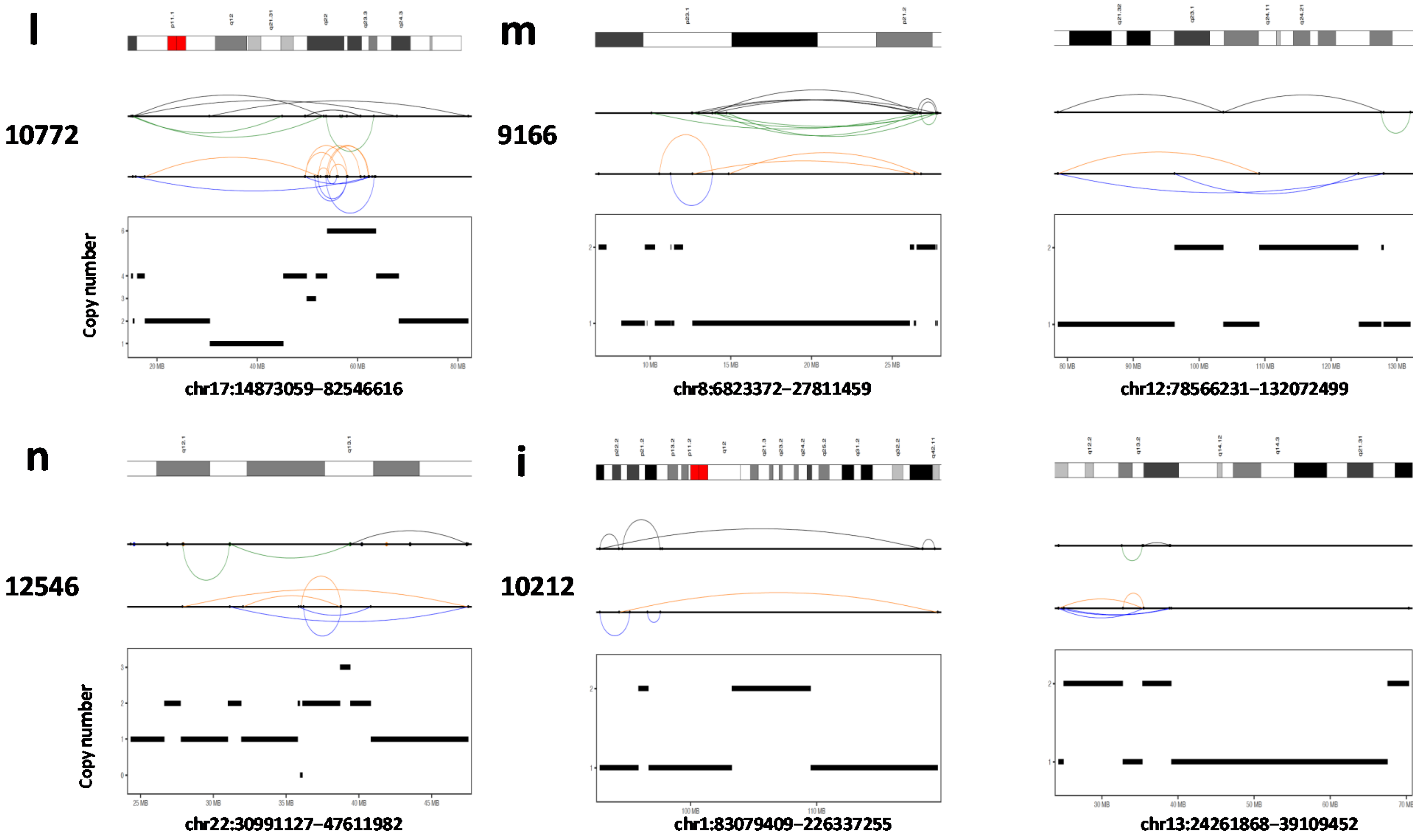
l
10772
Copy number
chr17:14873059–82546616
m
9166
chr8:6823372–27811459
chr12:78566231–132072499
n
12546
Copy number
chr22:30991127–47611982
i
10212
chr1:83079409–226337255
chr13:24261868–39109452

o

13029

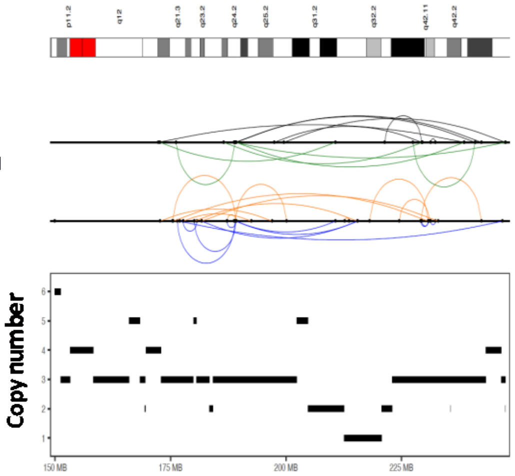

p

10801

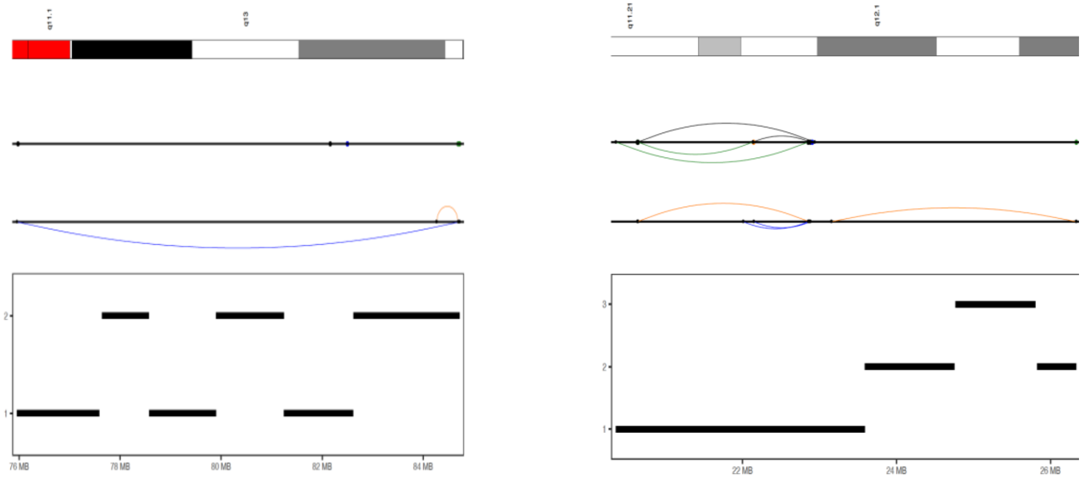

q

11668

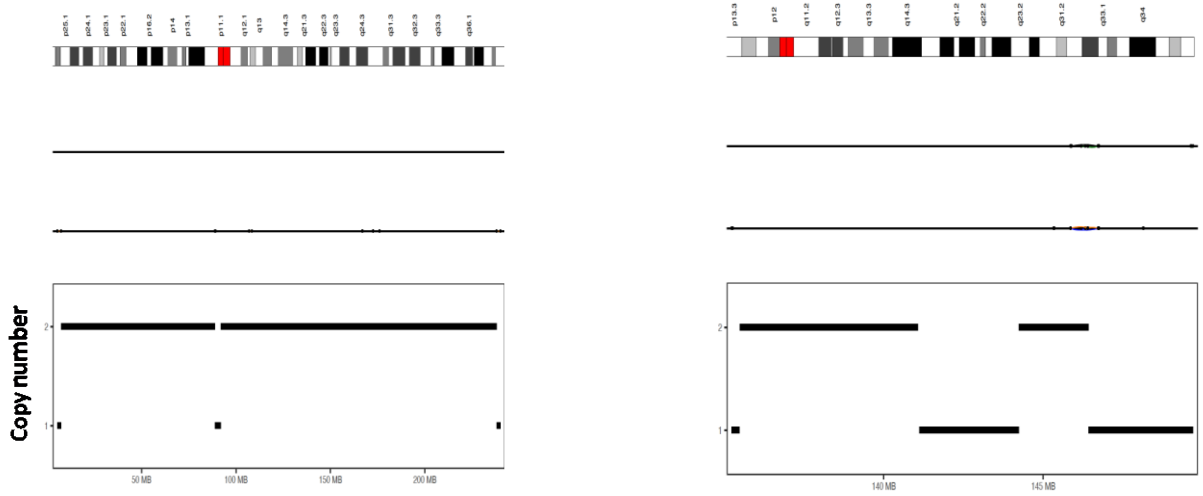

Supplementary Figure 3: Chromothripsis events in primary tumours. Each block of diagram represents chromothripsis event at individual chromosome. For each block, the top panel indicates genomic location of the chromothripsis event, the middle panel shows structural variants, and the bottom panel shows total copy number calls for the genomic region.

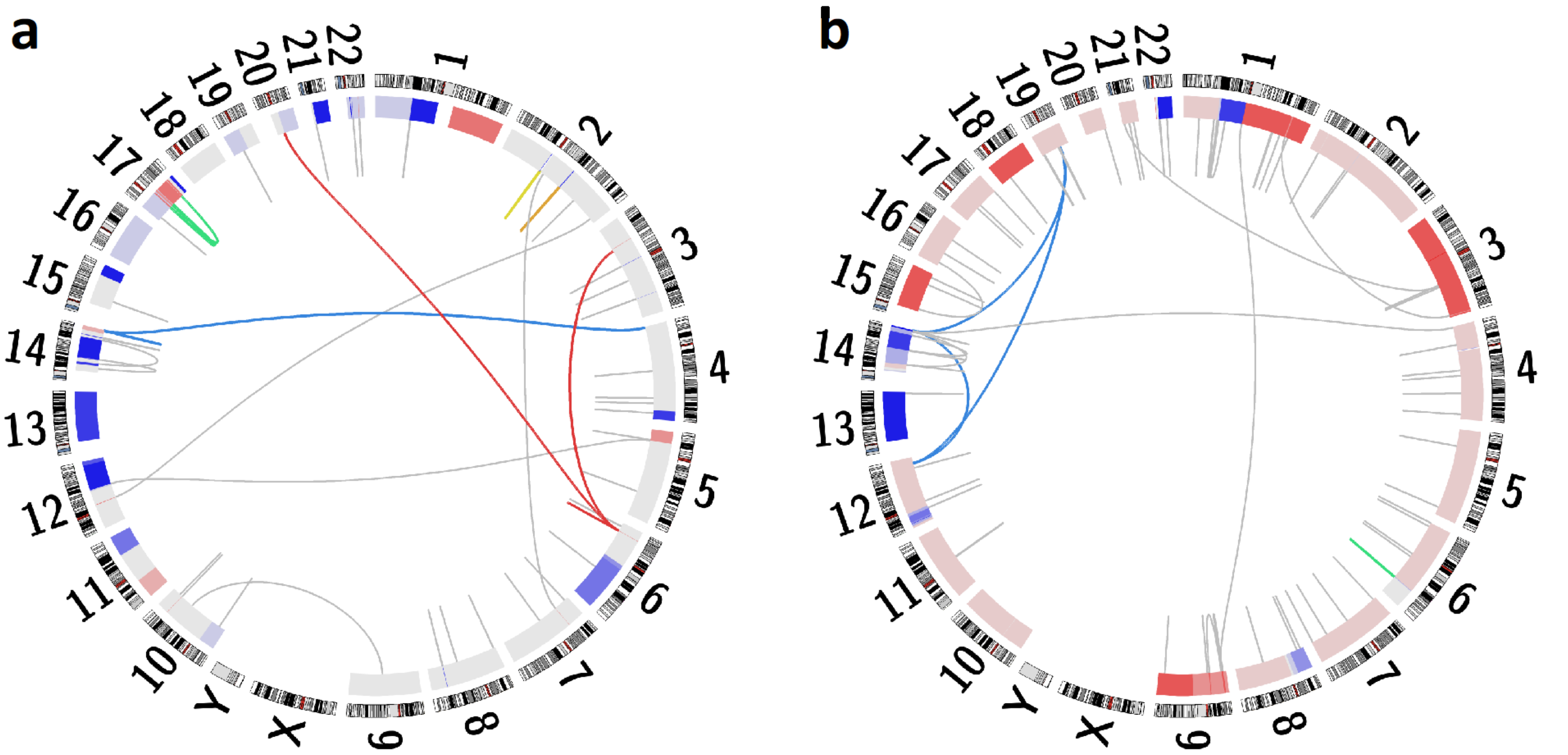

Supplementary Figure 4: Chromplexy events in primary tumours. Circos plots depicting rerrangement chains in tumours (a) 7880 and (b) 10068. Rearrangements in the same chains are in the same colour. Grey arrangements were not assigned to a chain. The inner rings show copy number gain and loss in red and blue, respectively. Chromoplexy events are those in red chain and blue chain for tumours (a) 7880 and (b) 10068 respectively.

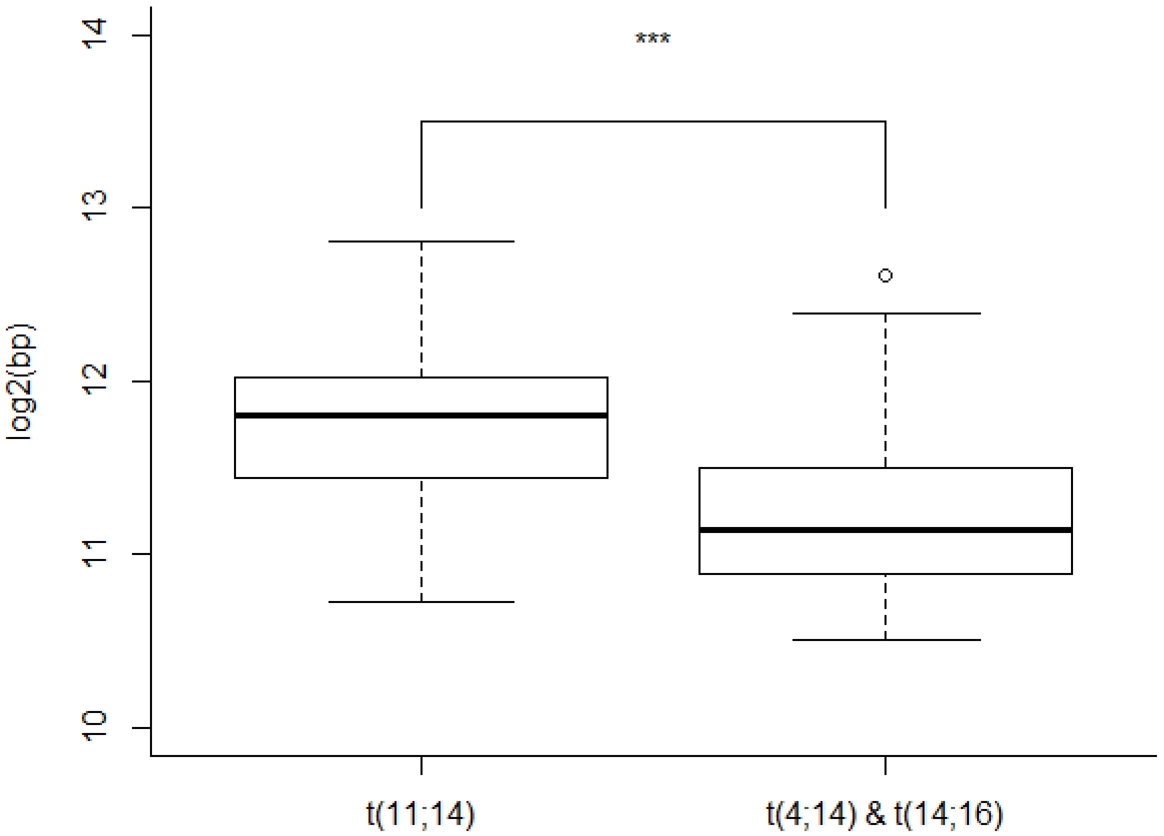

Supplementary Figure 5: Comparison of telomere lengths between subtypes. Boxplots show log_2 of telomere length base pairs (bp) of high-risk subtypes t(4;14) and t(14;16) versus lower-risk t(11;14). Whisker bar extend within $\pm$ 1.5 $\times$ interquartile range. ***: $P < 0.001$

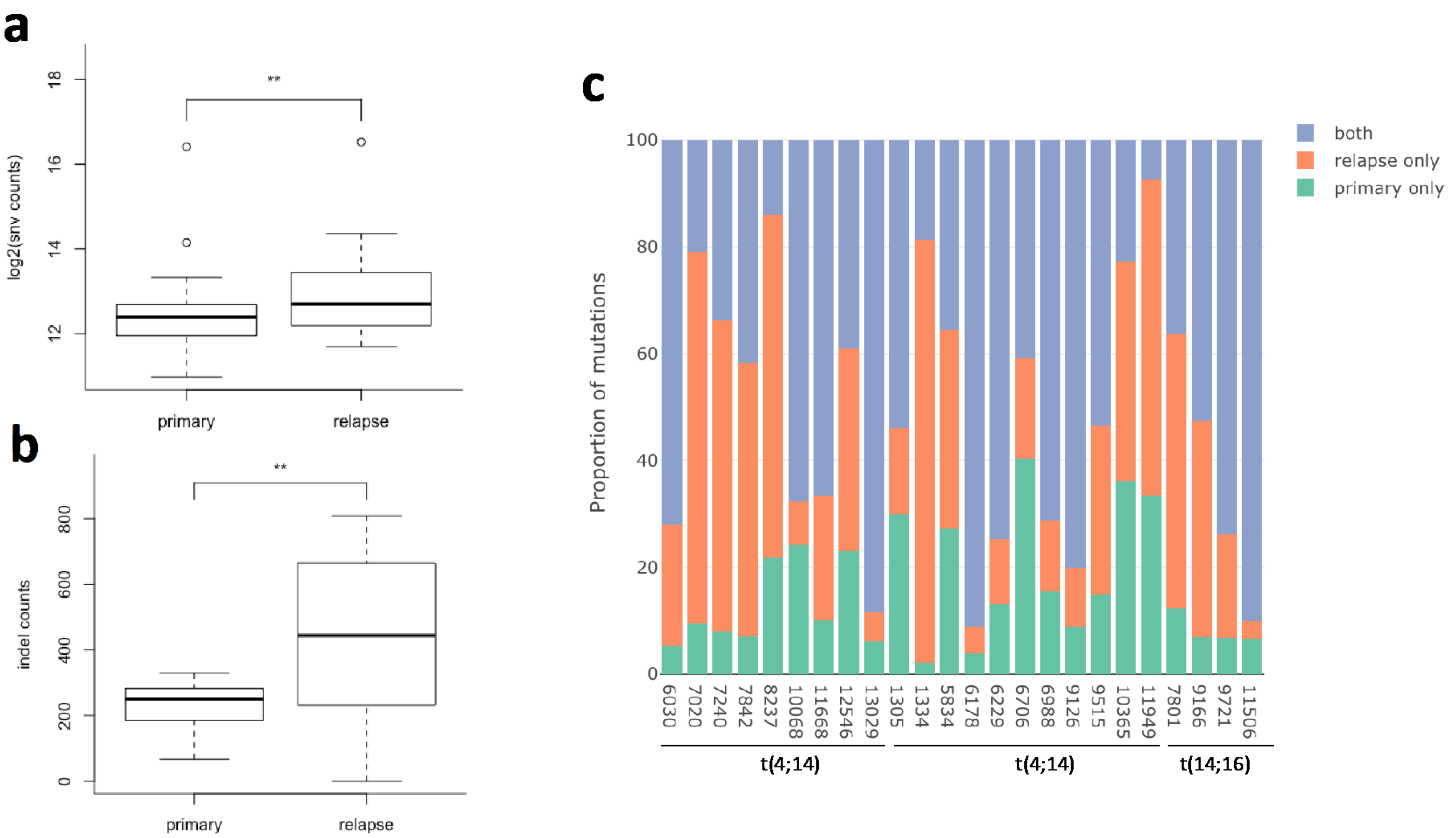

Supplementary Figure 6: Mutational burdens in primary versus relapsed tumours. Boxplots show (a) $\log_2$ of point mutation counts and (b) indel counts in primary and matched relapse tumours. Whisker bar extend within $\pm$ 1.5 $\times$ interquartile range. (c) Proportions of shared, relapse-specific and primary-specific mutations across samples. **: $P < 0.01$

a **b**

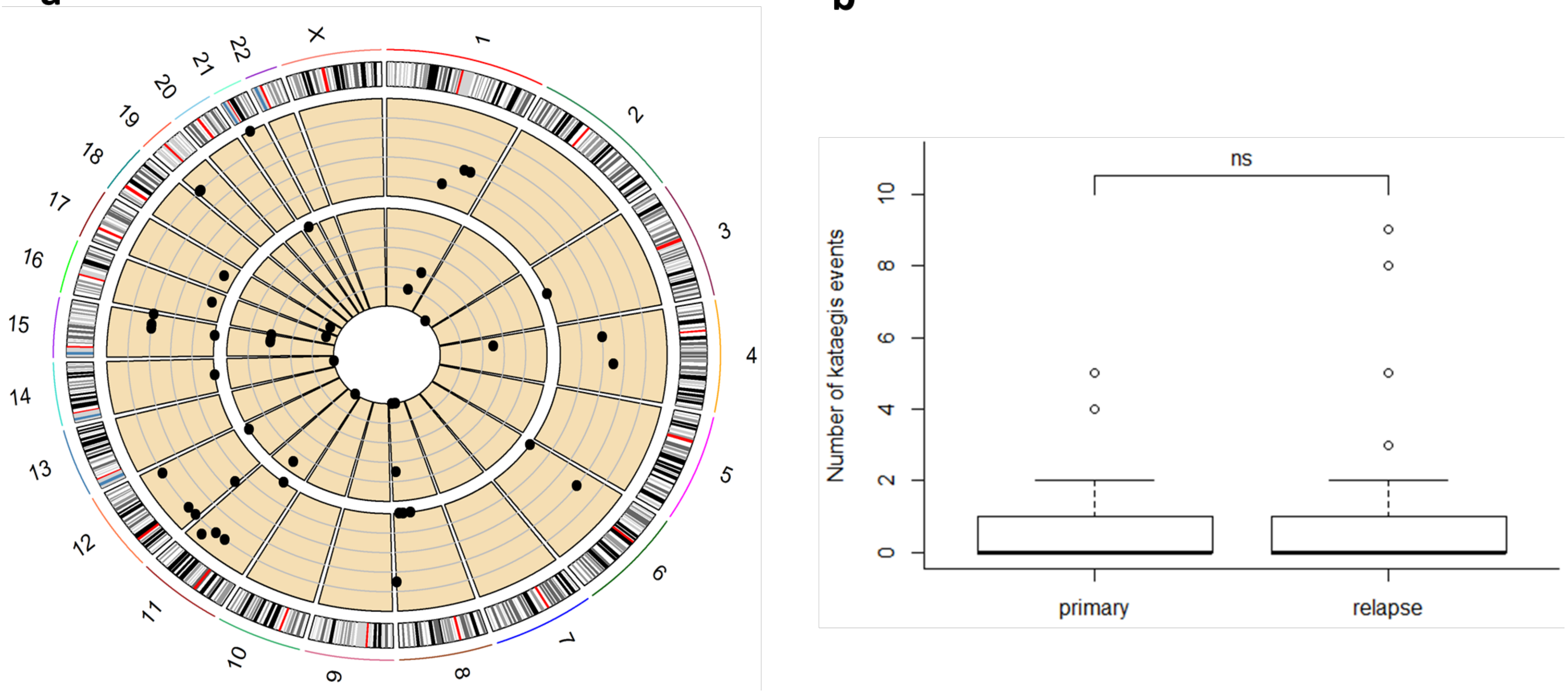

Supplementary Figure 7: Kataegis events in primary versus relapse. (a) Circos plot summarising kataegis foci detected in 24 primary (inner circle) and their matched relapse tumours (outer circle). Each dot represents a kataegis event, positioned by distinct samples based on height and genomic location based on width of the circle. (b) Boxplot show number of kataegis events detected in per primary versus relapse tumours. Whisker bar extend within ± 1.5 × interquartile range. ns: not significant.

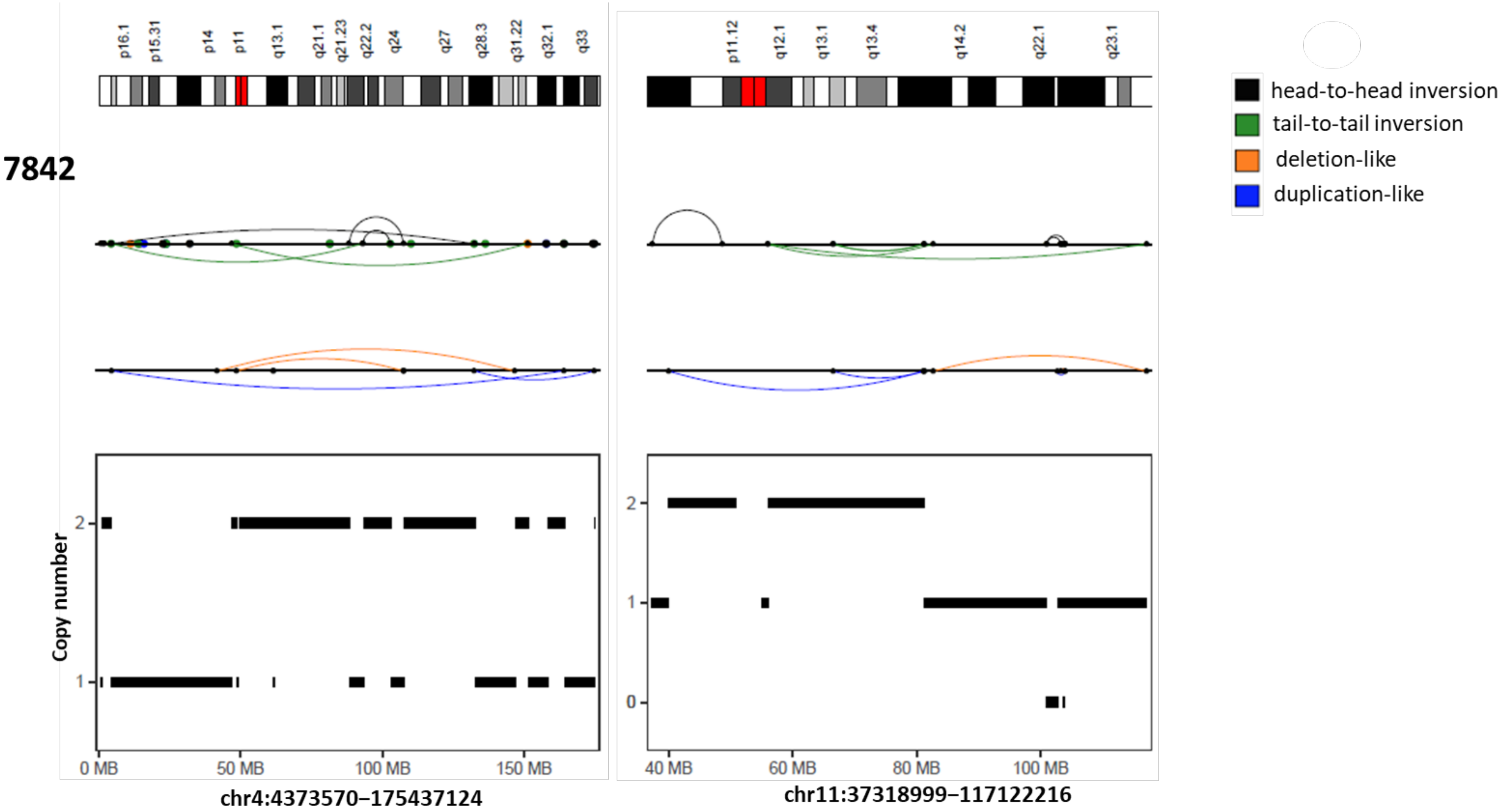

Supplementary Figure 8: Additional chromothripsis events detected in relapsed tumour. Chromothripsis previously unidentified in primary detected in relapse tumour sample 7842. Each block of diagram represents chromothripsis event at individual chromosome. For each block, the top panel indicates genomic location of the chromothripsis, the middle panel shows consensus structural variants, and the bottom panel shows total copy number calls for the genomic region.

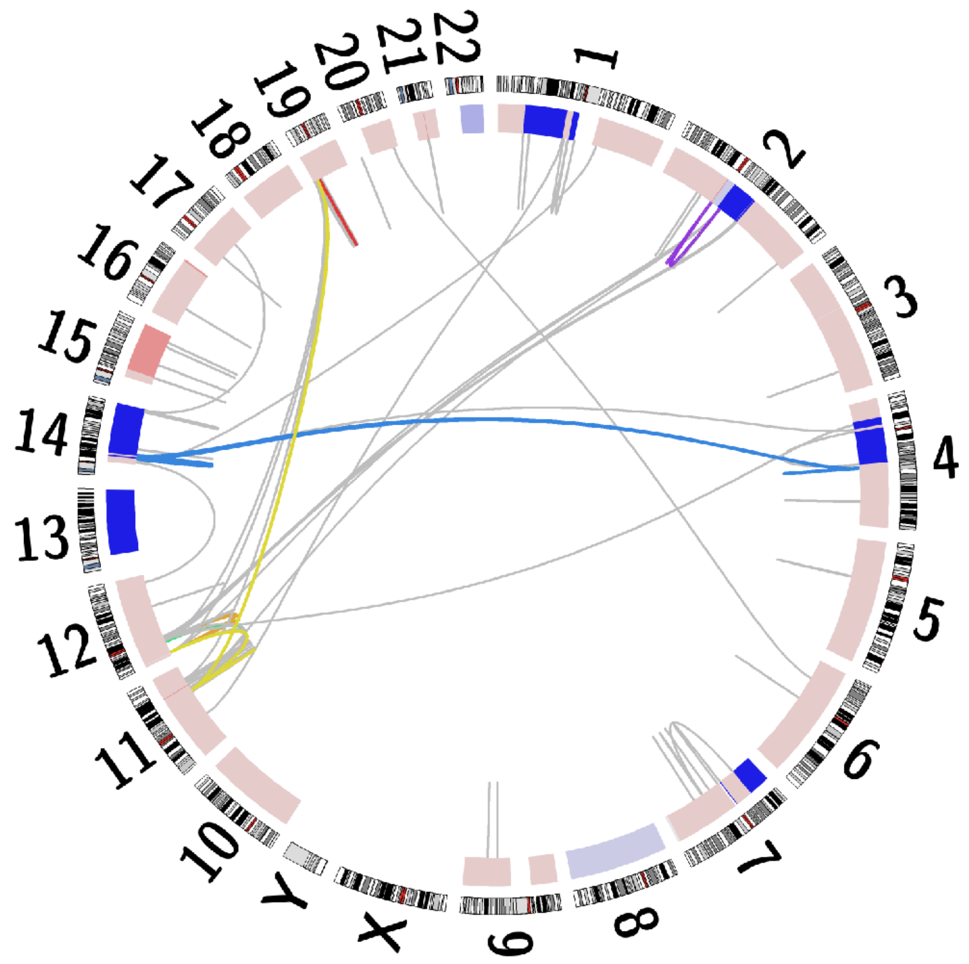

Supplementary Figure 9: Additional chromplexy events in relapsed tumour. Circos plots depicting rerrangement chains in tumour 8237. Rearrangements in the same chains are in the same colour. Grey arrangements were not assigned to a chain. The inner rings show copy number gain and loss in red and blue, respectively. Chromoplexy events are those in yellow chain.

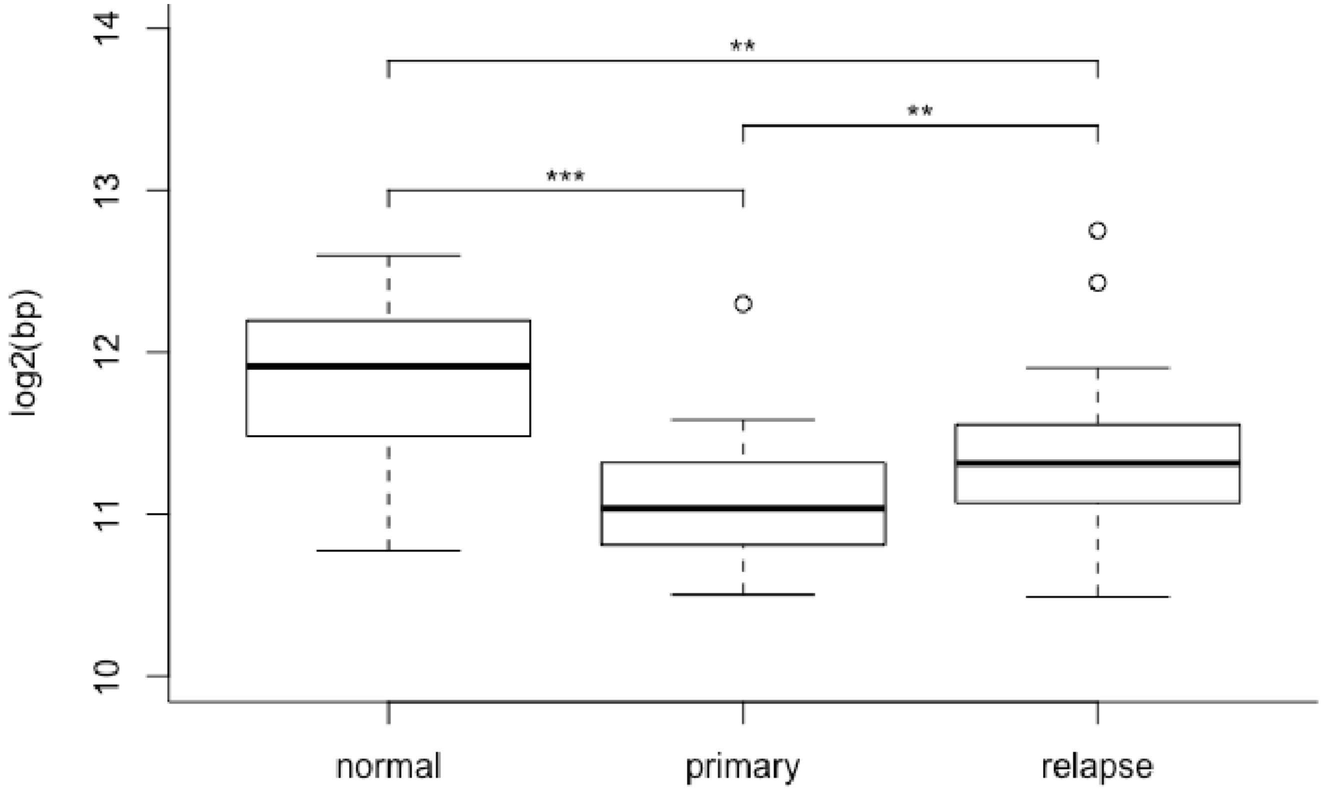

Supplementary Figure 10: Telomere length comparison. Boxplots show log_2 (base pair) of telomere lengths of 24 matched normal, primary, and relapse samples. Whisker bars extend to $\pm$ 1.5 × interquartile range. **: $P < 0.01$; ***: $P < 0.001$.

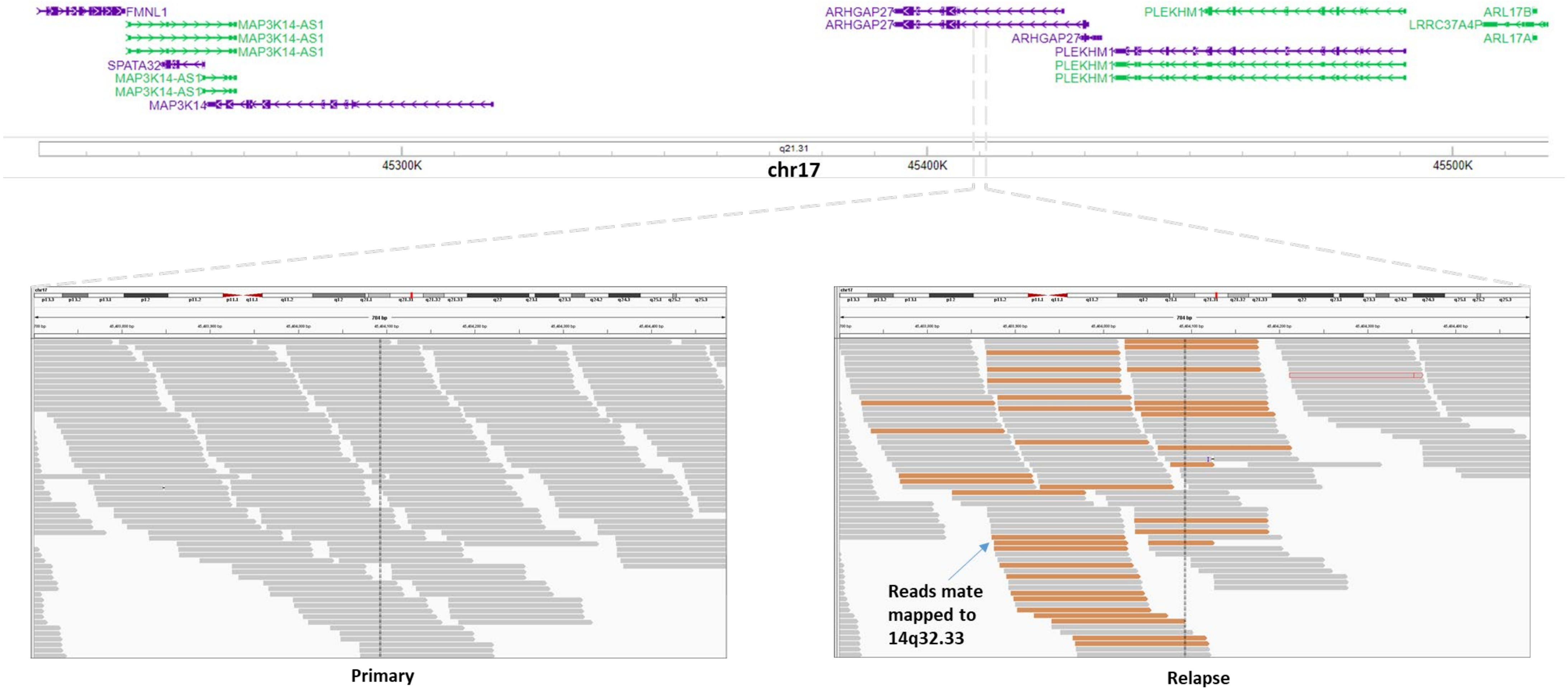

Supplementary Figure 11: Acquisition of chromosomal translocation in proximity to *MAP3K14* at relapse in sample 8237. Upper panel shows relative location of chromosomal translocation to *MAP3K14*. Lower panels show IGV screenshots indicating **de novo** acquisition of chromosomal translocation (14q32;17q21) at relapse (right panel) not present in primary (left panel).

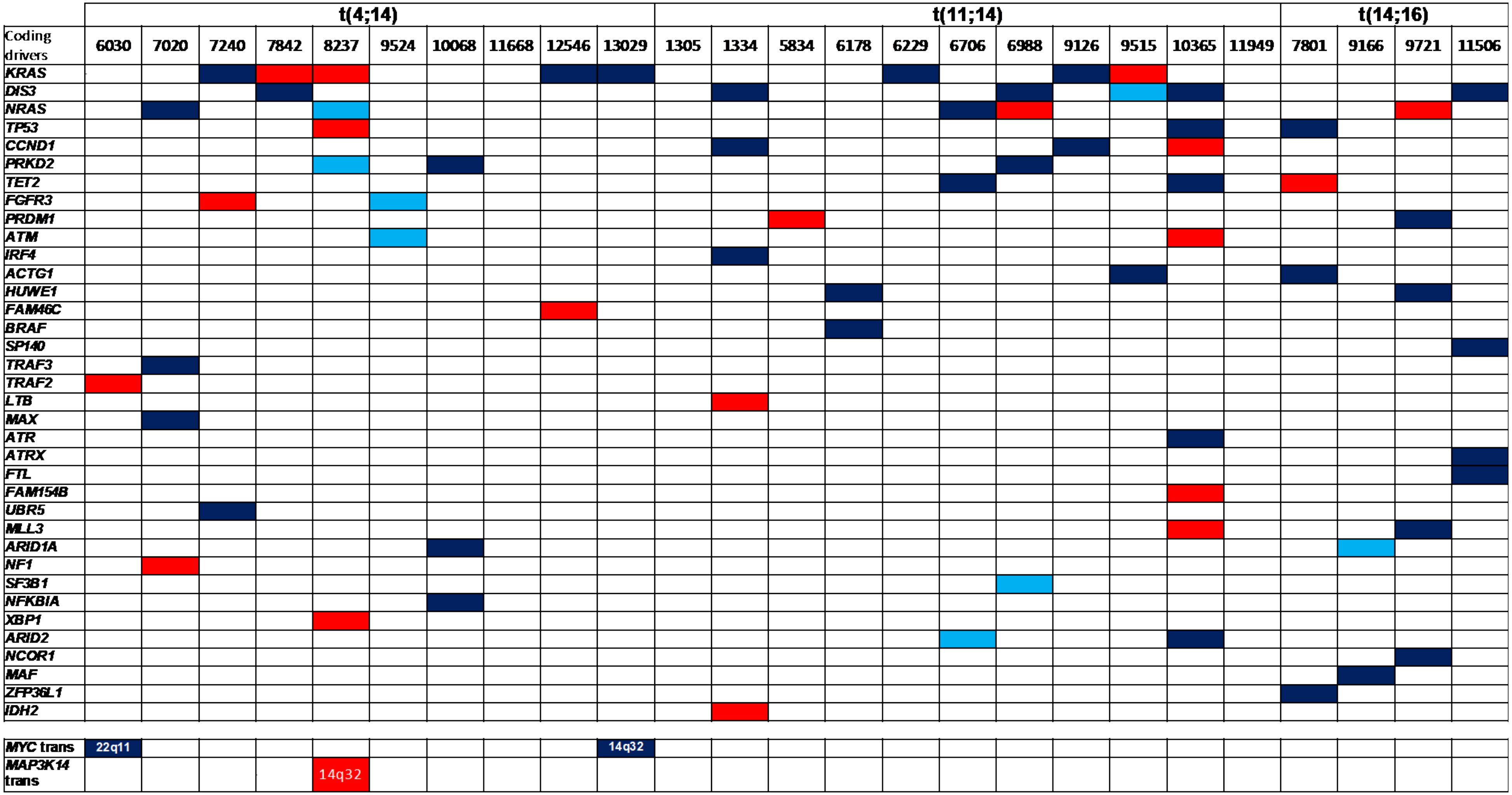

Supplementary Figure 12: Non-silent single nucleotide variants and indels disrupting established driver genes, and established translocations, in primary and matched relapsed tumours.

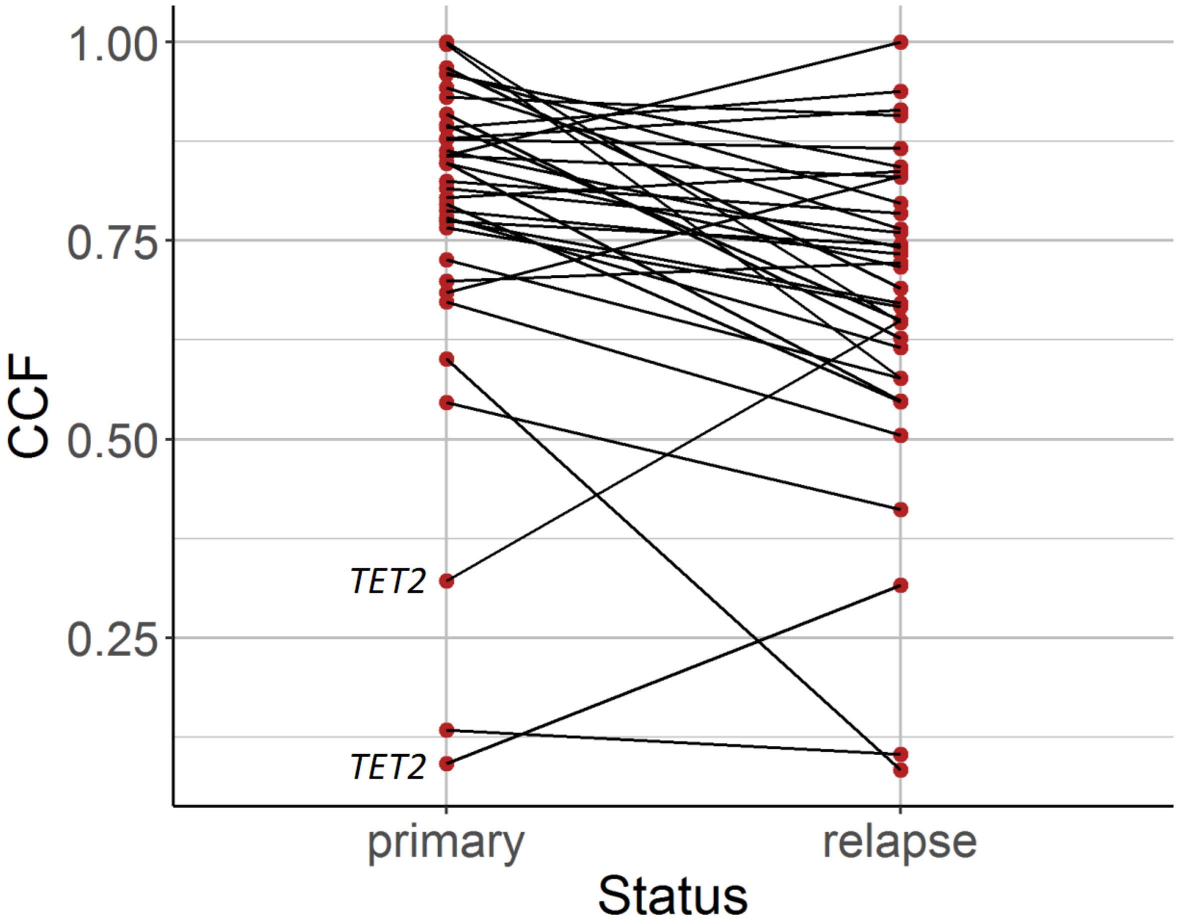

Supplementary Figure 13: Cancer cell fractions (CCFs) of coding driver genes in primary and relapsed tumours. Each dot represents a non-silent mutation in a driver gene. Relationships between CCF of a driver gene mutation in primary and relapse are indicated by the lines linking them. Genes with a large increase in CCF at relapse (*i.e.* clonal expansion of subclones carrying the mutations) are annotated.

t(4;14) **Primary** **Relapse**

6030

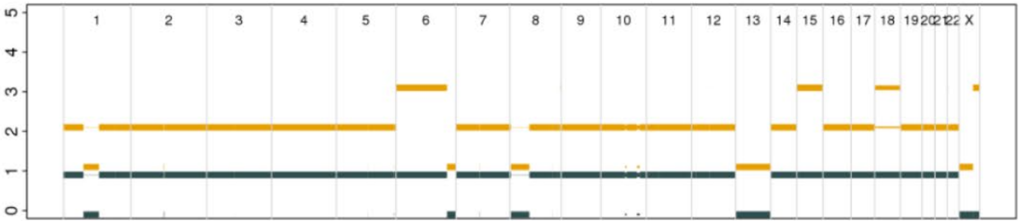

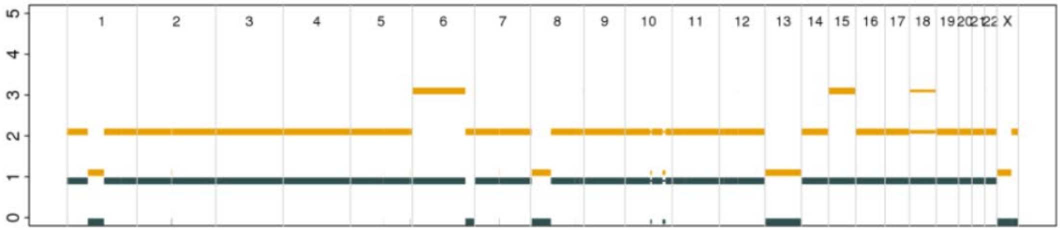

7020

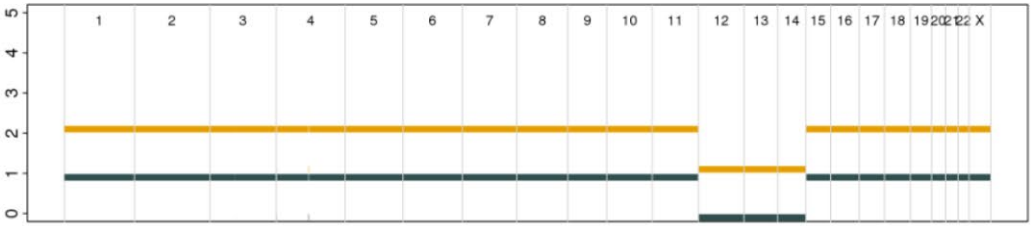

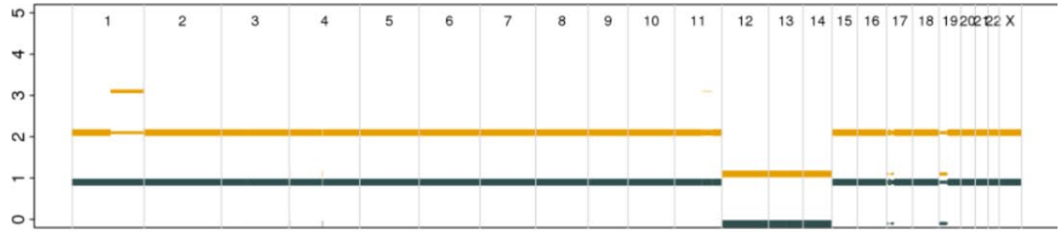

7240

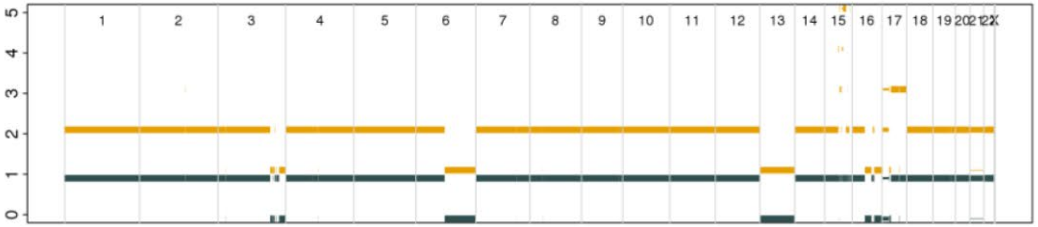

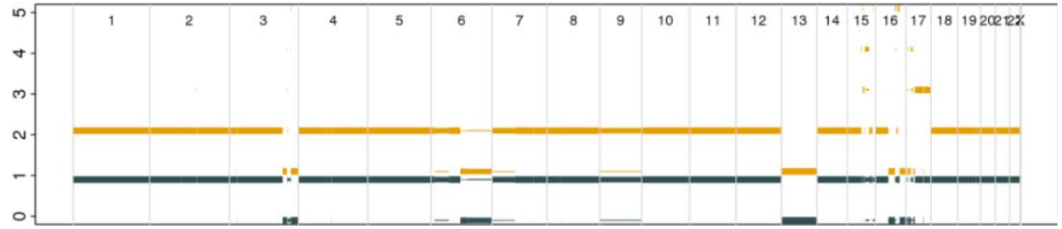

7842

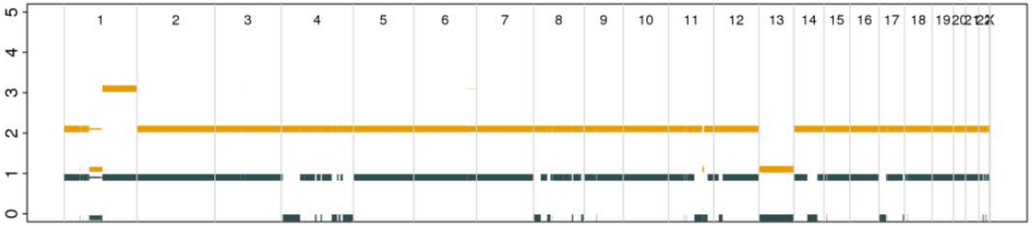

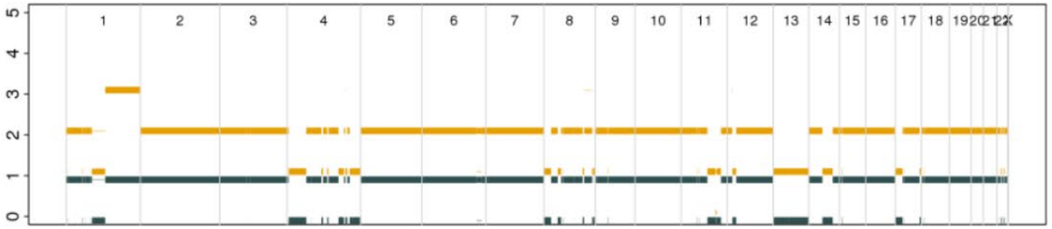

8237

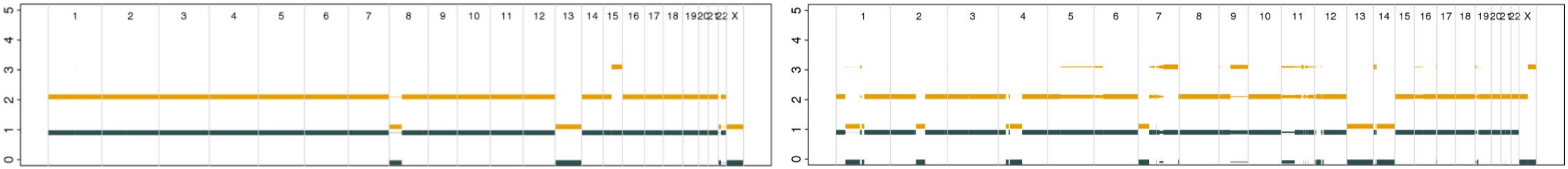

10068

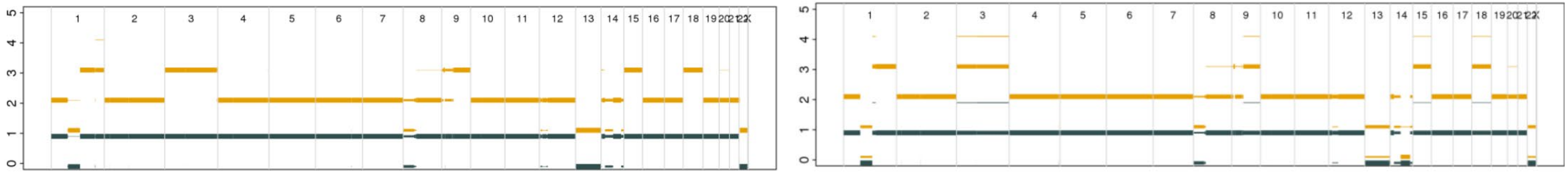

11668

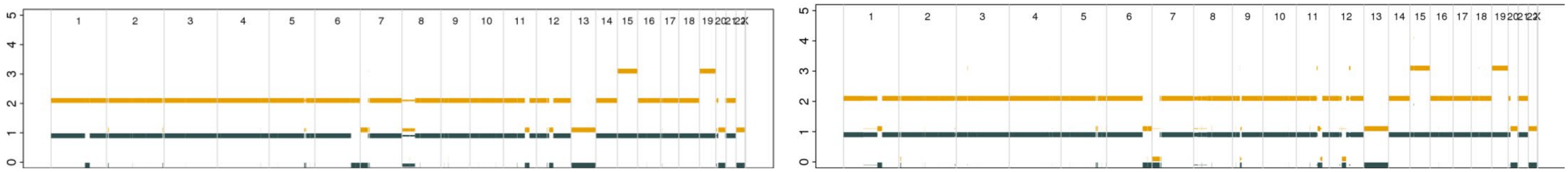

12546

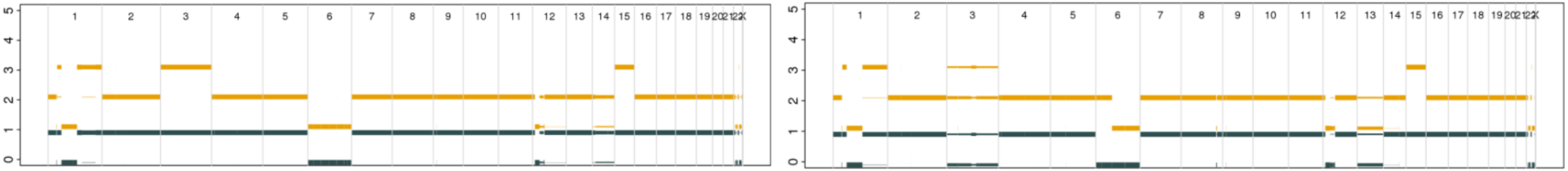

13029

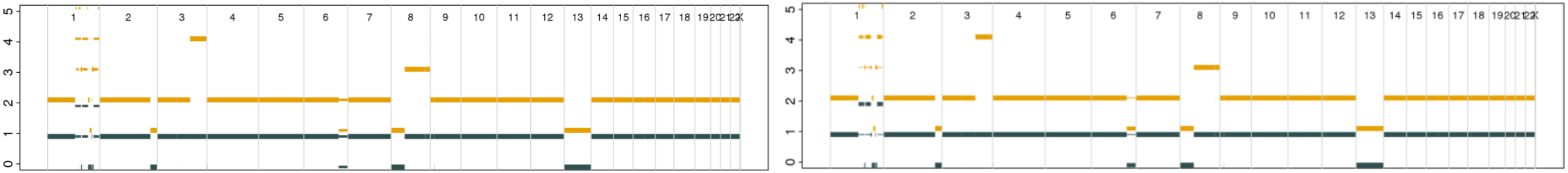

t(11;14)

1305

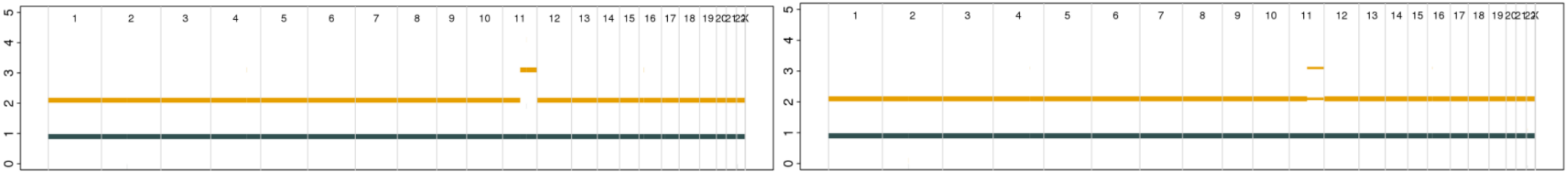

1334

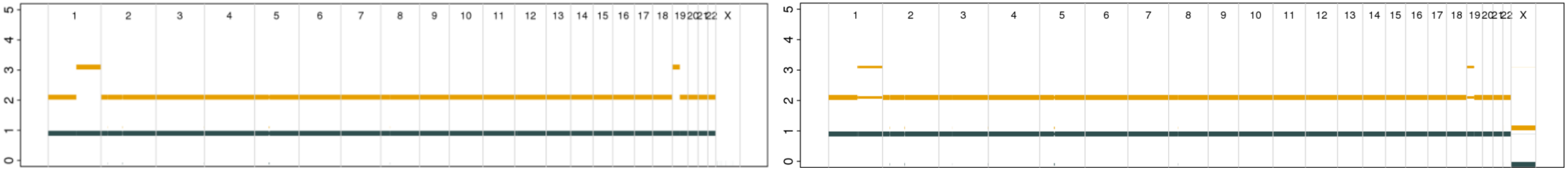

5834

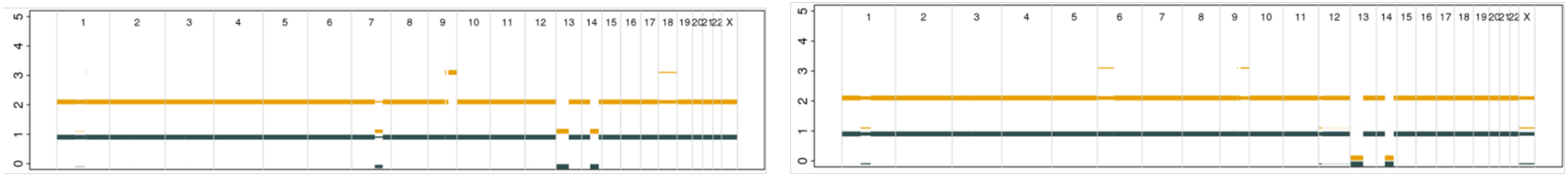

6178

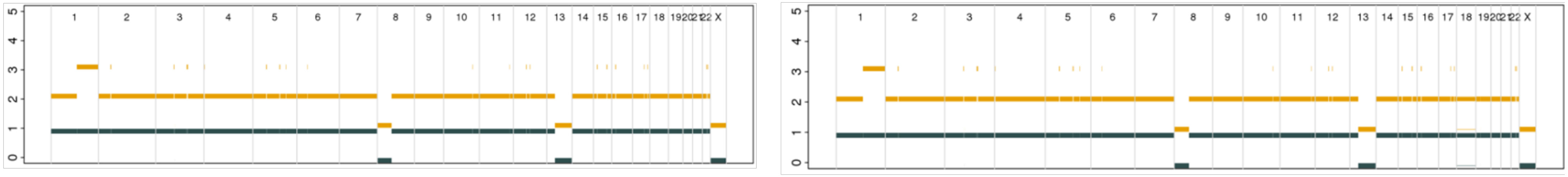

6229

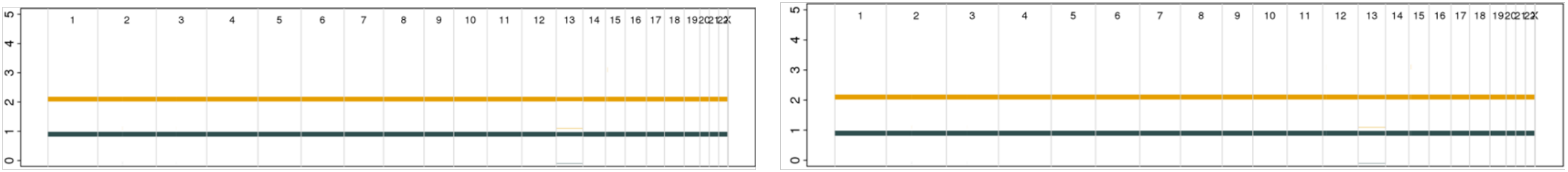

6706

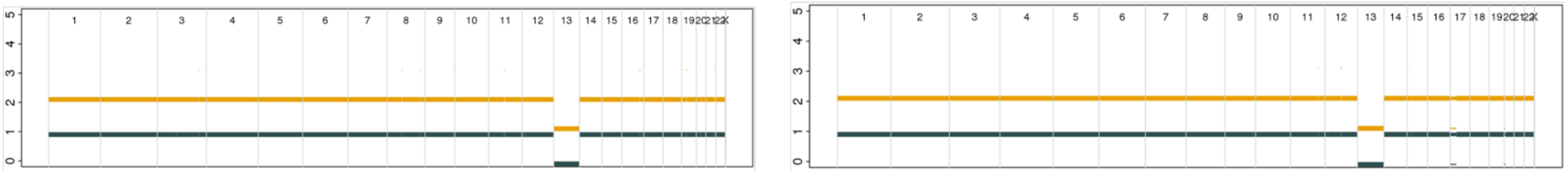

6988

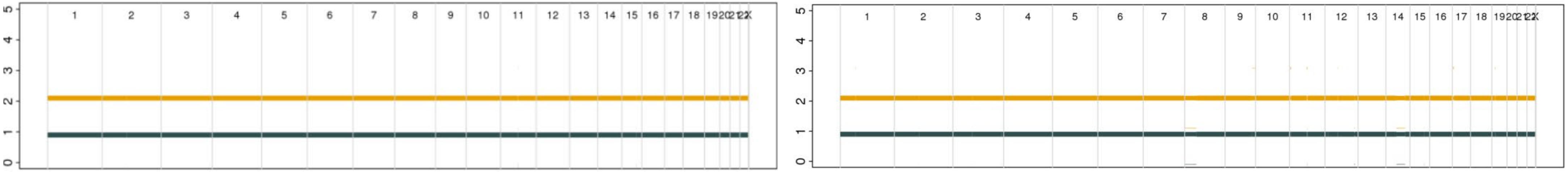

9126

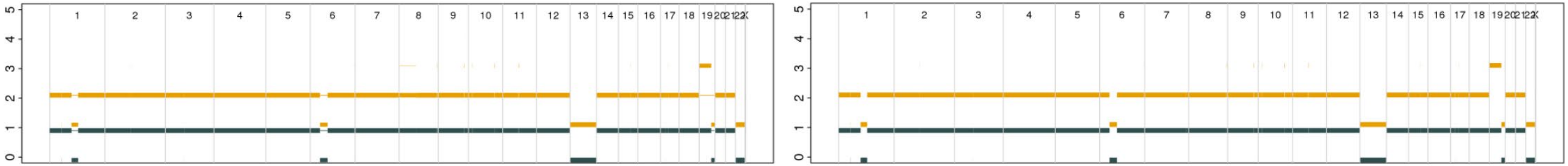

9515

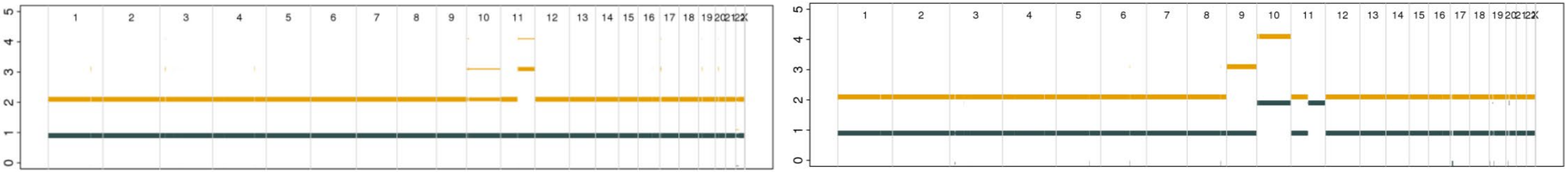

10365

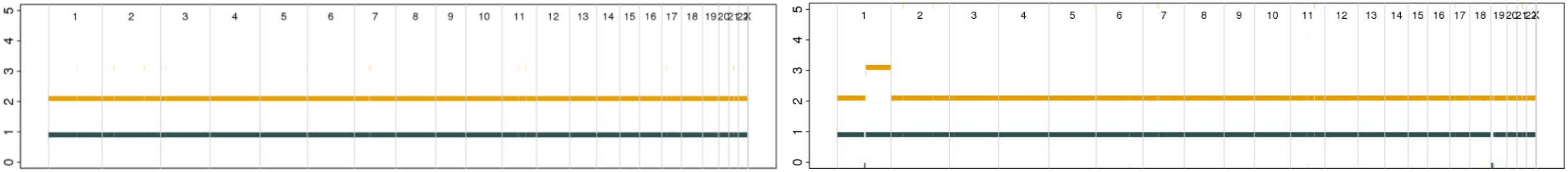

11949

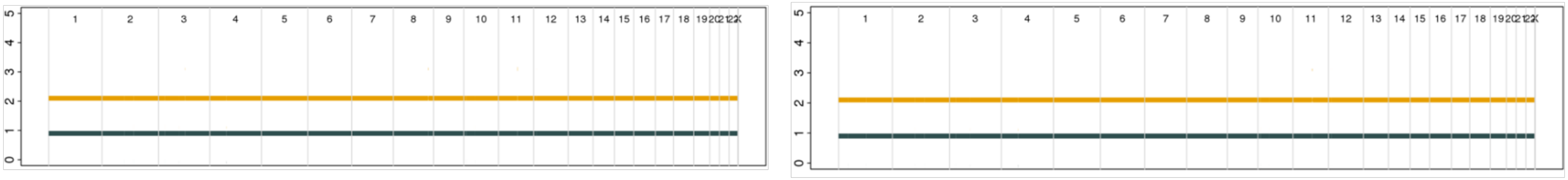

t(14;16)

7801

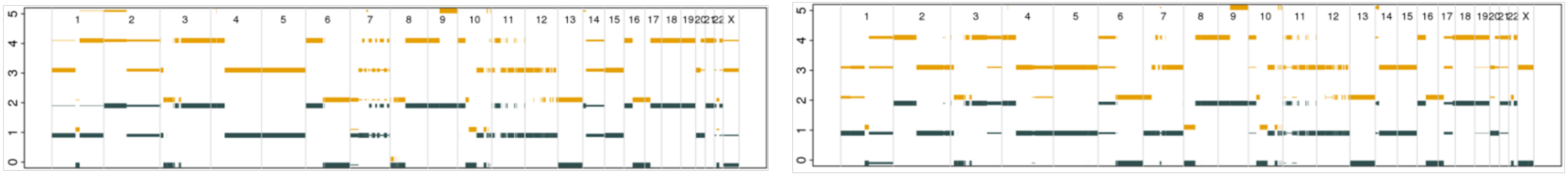

9166

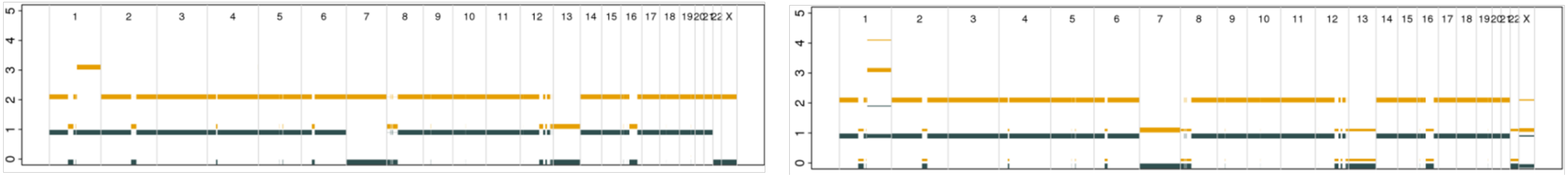

9721

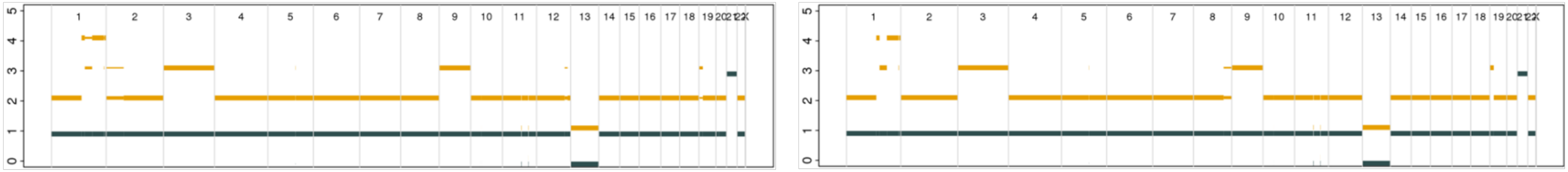

11506

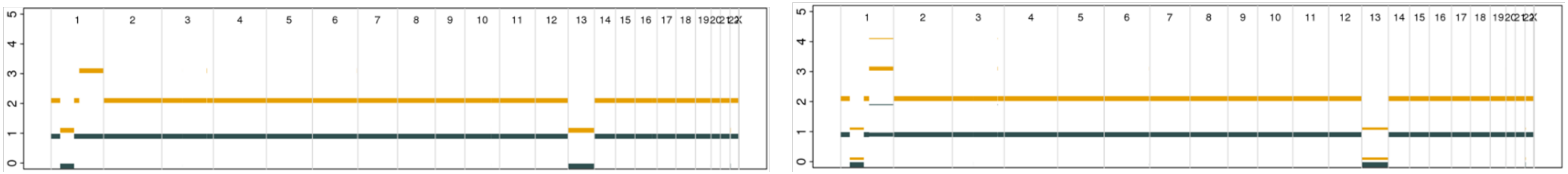

Supplementary Figure 14: Copy number plots for 24 matched primary (left) and relapsed (right) tumours. Clonal copy numbers are represented as solid line with higher intensity than subclonal copy number changes represented as thin line. Y-axis: copy number, x-axis: chromosomes. Yellow: total copy number, dark blue: copy number of the minor allele.

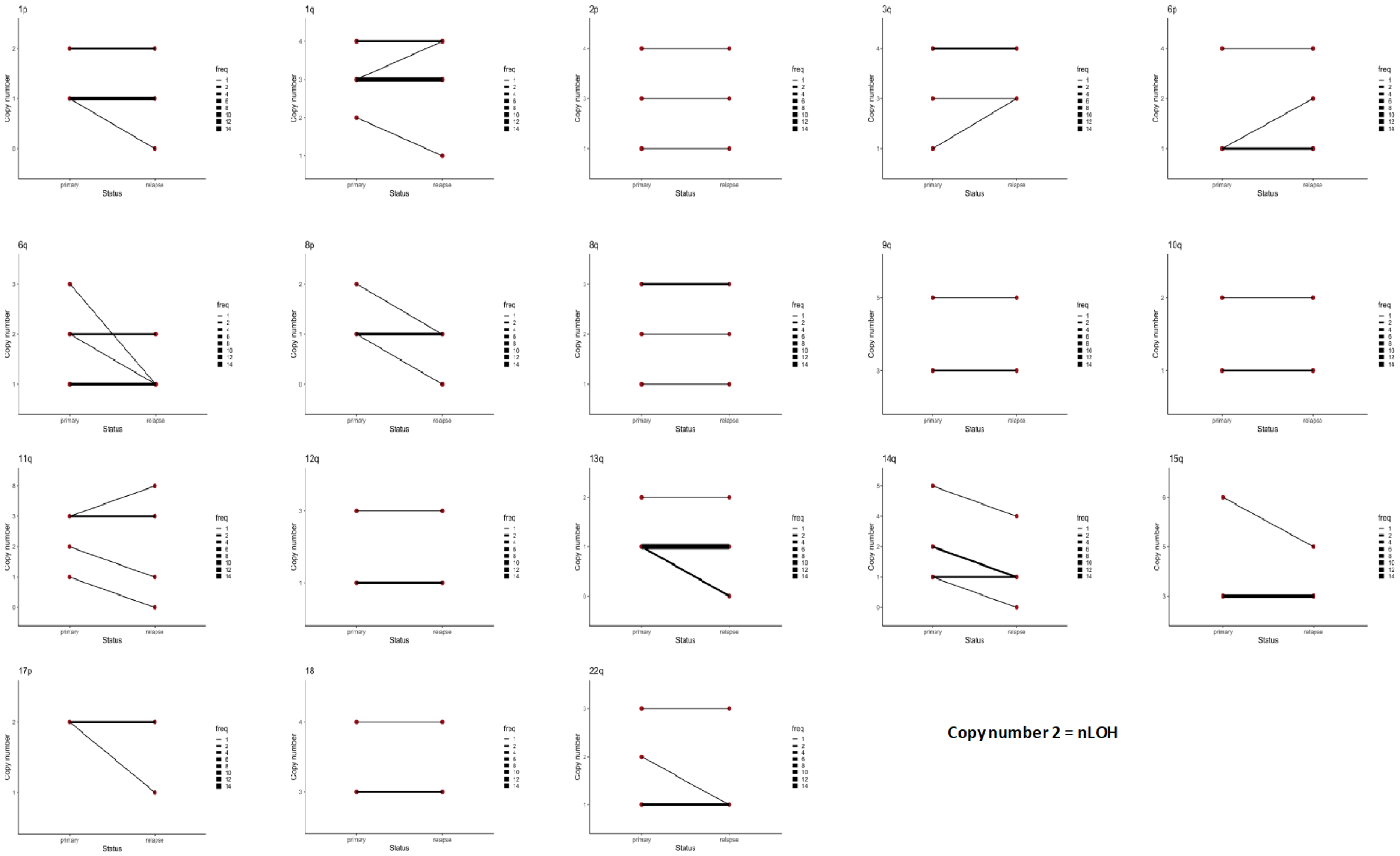
1p
1q
2p
3q
6p
6q
8p
8q
9q
10q
11q
12q
13q
14q
15q
17p
18
22q
Copy number
Status
primary
relapse
freq
Copy number 2 = nLOH

Supplementary Figure 15: Patterns of major copy number changes in primary and relapsed tumours. Lines connecting dots indicate relationship between primary and matched relapsed tumours. The intensity of lines is proportional to frequency (freq) of events. Only chromosomes or chromosome arms with copy number variations are plotted, thus copy number of 2 is copy number neutral loss of heterozygosity (LOH).

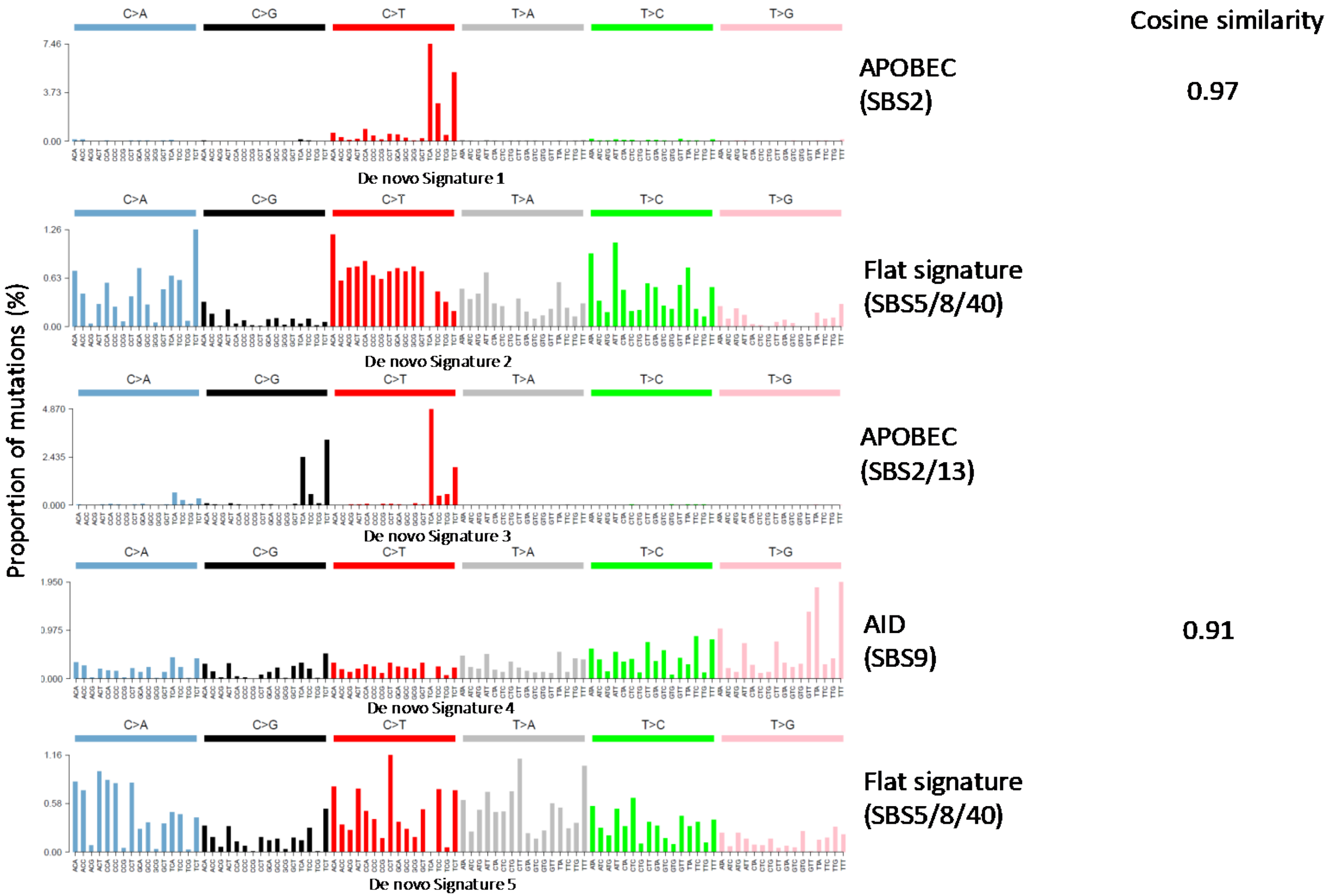

Supplementary Figure 16: *De novo* extraction of WGS single nucleotide variants signatures using non-negative matrix factorization algorithm in 80 primary tumours. Left: Summary of five *de novo* mutational signatures extracted. Right: cosine similarity values of assigned COSMIC signatures. *De novo* extracted mutational signatures are compared against 85 COSMIC v3 single base substitution (SBS) signatures.

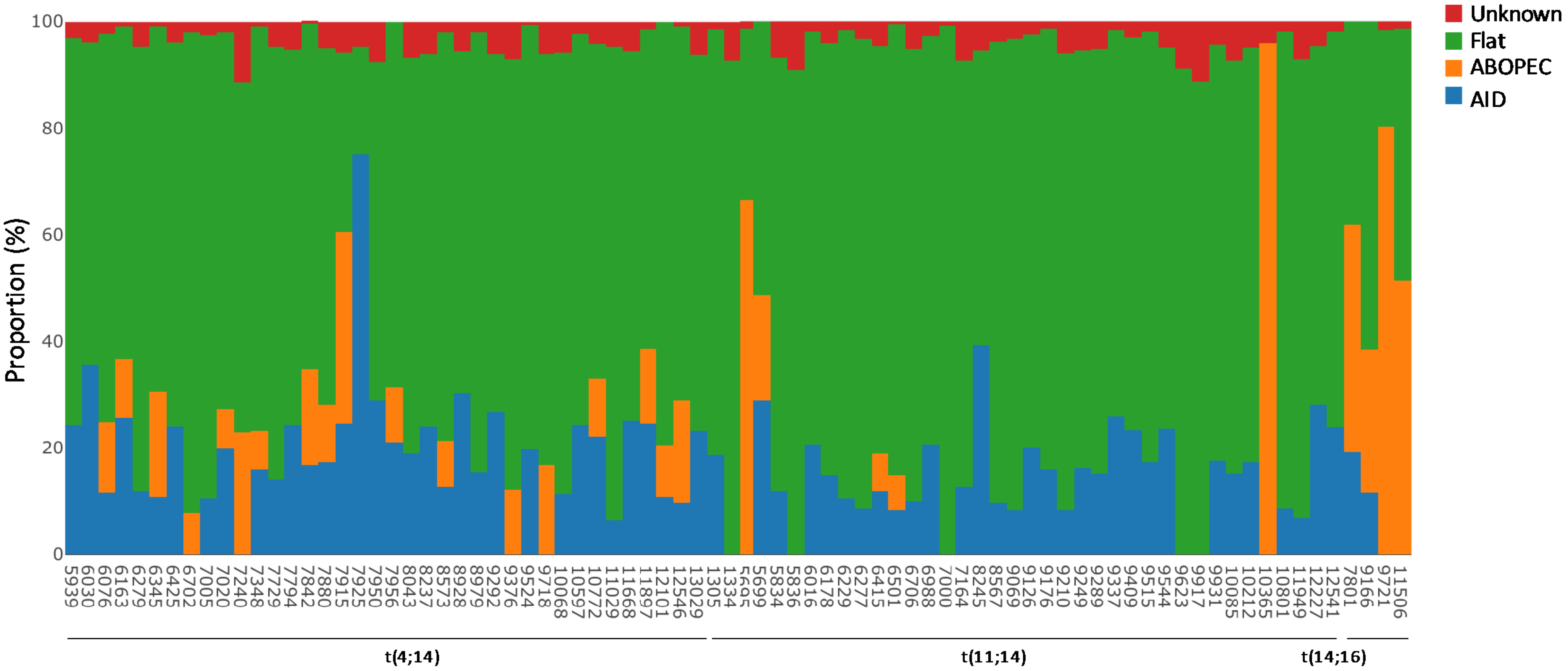

Supplementary Figure 17: Mutational signatures contribution across 80 primary tumours. Mutational signatures contribution fitting from deconstructSig. Only major COSMIC mutational signatures extracted *de novo* were considered. APOBEC signature includes SBS2 and 13. Flat signature includes SBS5, 8 and 40. AID: activation-induced deaminase (SBS9)

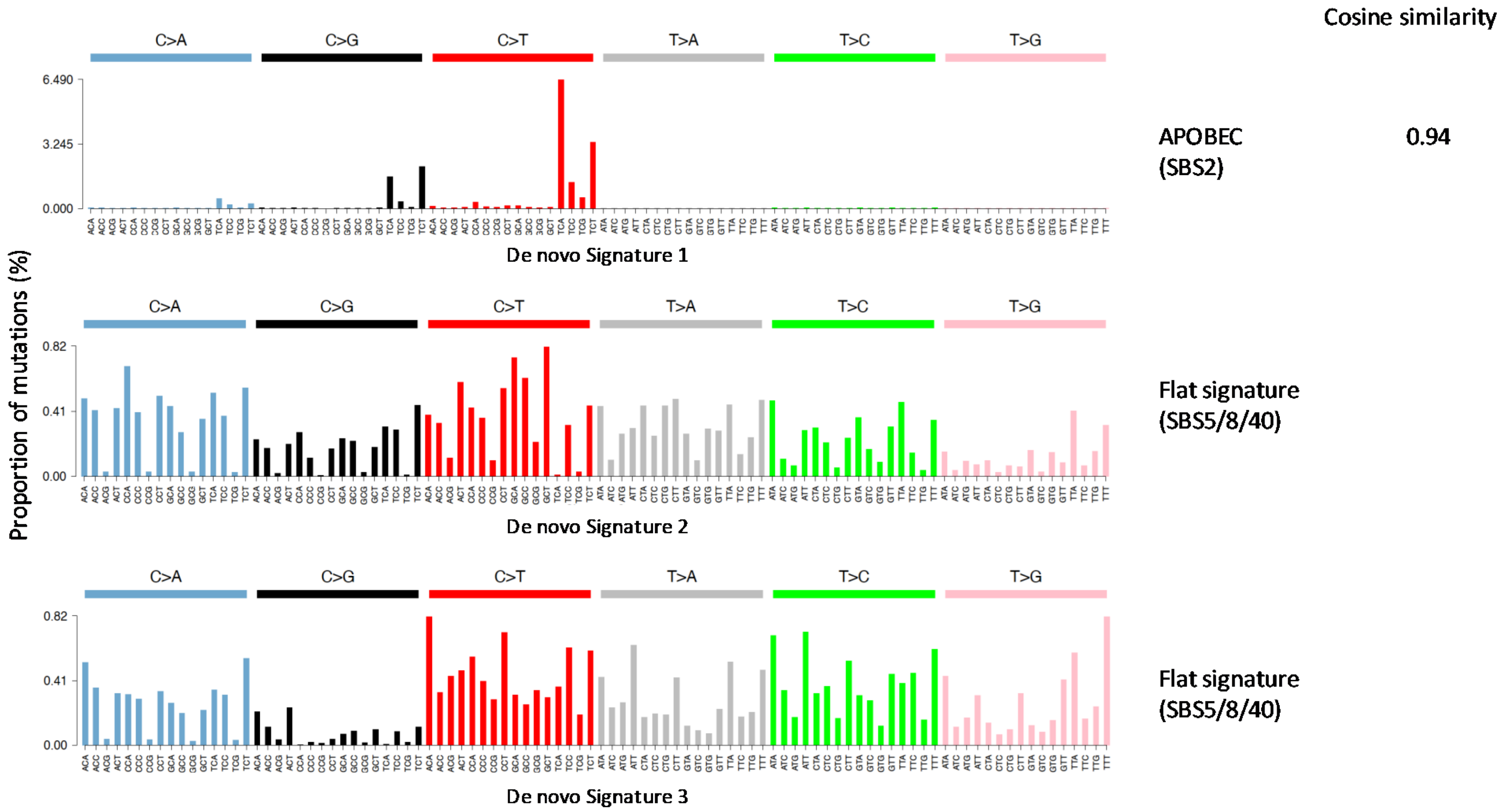

Supplementary Figure 18: *De novo* extraction of WGS single nucleotide variants signatures using non-negative matrix factorization algorithm in 24 relapsed tumours. Left: Summary of five *de novo* mutational signatures extracted. Right: cosine similarity values of assigned COSMIC signatures. *De novo* extracted mutational signatures are compared against 85 COSMIC v3 single base substitution (SBS) signatures.

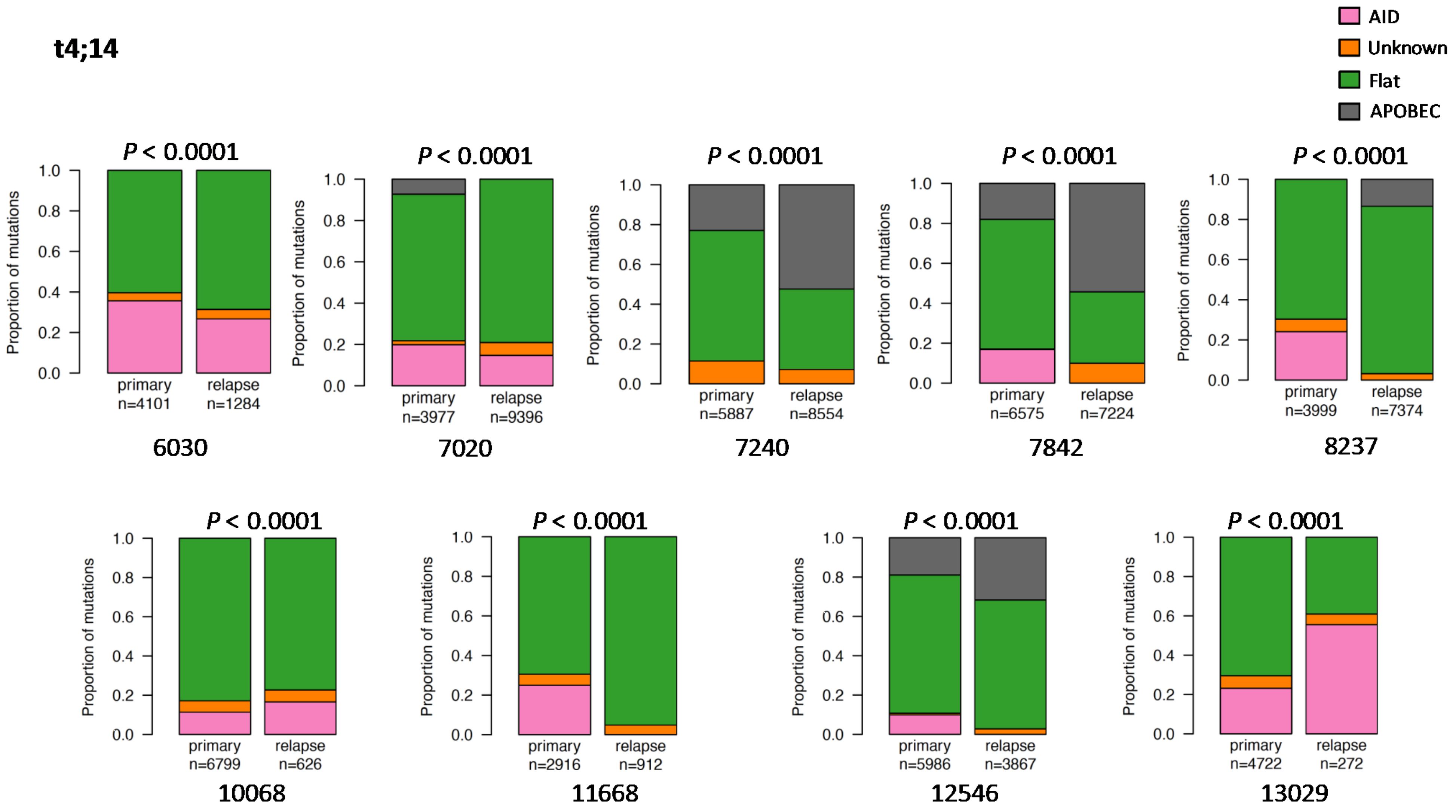
t4;14
AID
Unknown
Flat
APOBEC
P < 0.0001
Proportion of mutations
0.0
0.2
0.4
0.6
0.8
1.0
primary
n=4101
relapse
n=1284
6030
primary
n=3977
relapse
n=9396
7020
primary
n=5887
relapse
n=8554
7240
primary
n=6575
relapse
n=7224
7842
primary
n=3999
relapse
n=7374
8237
primary
n=6799
relapse
n=626
10068
primary
n=2916
relapse
n=912
11668
primary
n=5986
relapse
n=3867
12546
primary
n=4722
relapse
n=272
13029

t11;14

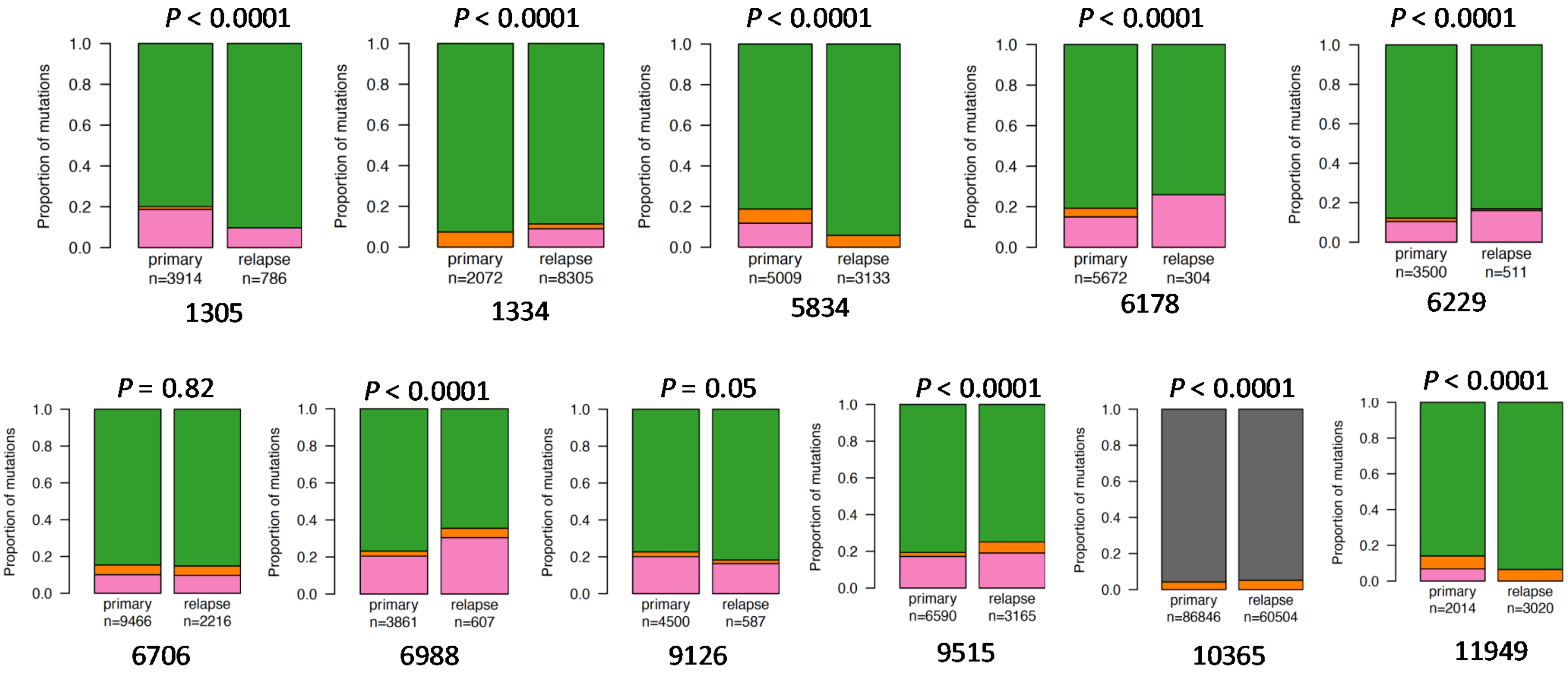
P < 0.0001
Proportion of mutations
primary n=3914
relapse n=786
1305
P < 0.0001
primary n=2072
relapse n=8305
1334
P < 0.0001
primary n=5009
relapse n=3133
5834
P < 0.0001
primary n=5672
relapse n=304
6178
P < 0.0001
primary n=3500
relapse n=511
6229
P = 0.82
primary n=9466
relapse n=2216
6706
P < 0.0001
primary n=3861
relapse n=607
6988
P = 0.05
primary n=4500
relapse n=587
9126
P < 0.0001
primary n=6590
relapse n=3165
9515
P < 0.0001
primary n=86846
relapse n=60504
10365
P < 0.0001
primary n=2014
relapse n=3020
11949

t14;16

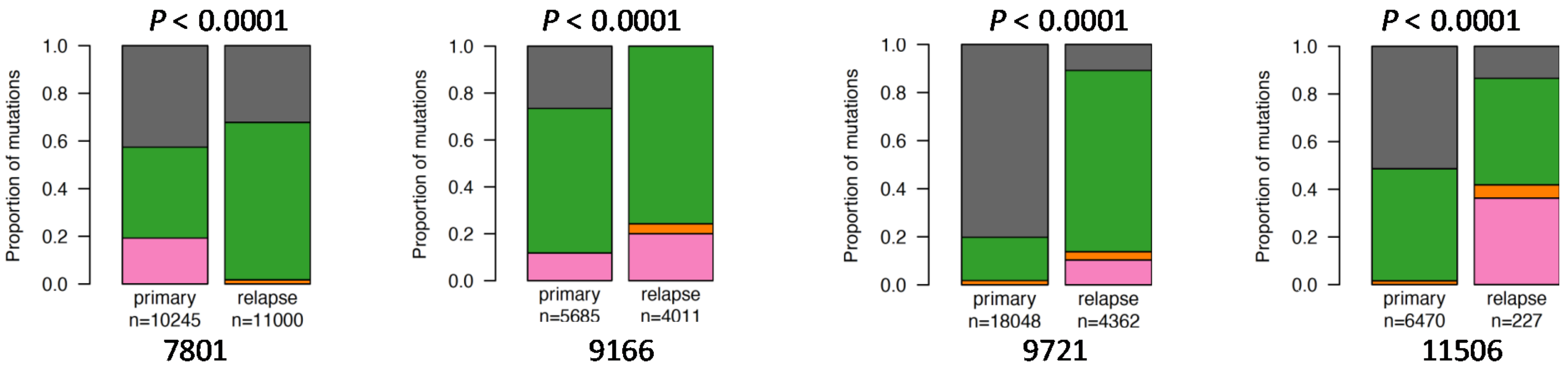

Supplementary Figure 19: Mutation signatures contribution in primary versus relapsed tumours. Stacked bar charts showing comparisons of major mutational signatures between primary versus relapse-specific mutations. The *P*-values refer to the overall difference in distribution between primary and relapse-specific mutations (chi-squared test). n = number of mutations. AID is attributed to SBS9. APOBEC includes SBS2 and 13. Flat signatures include SBS5, 8, and 40.

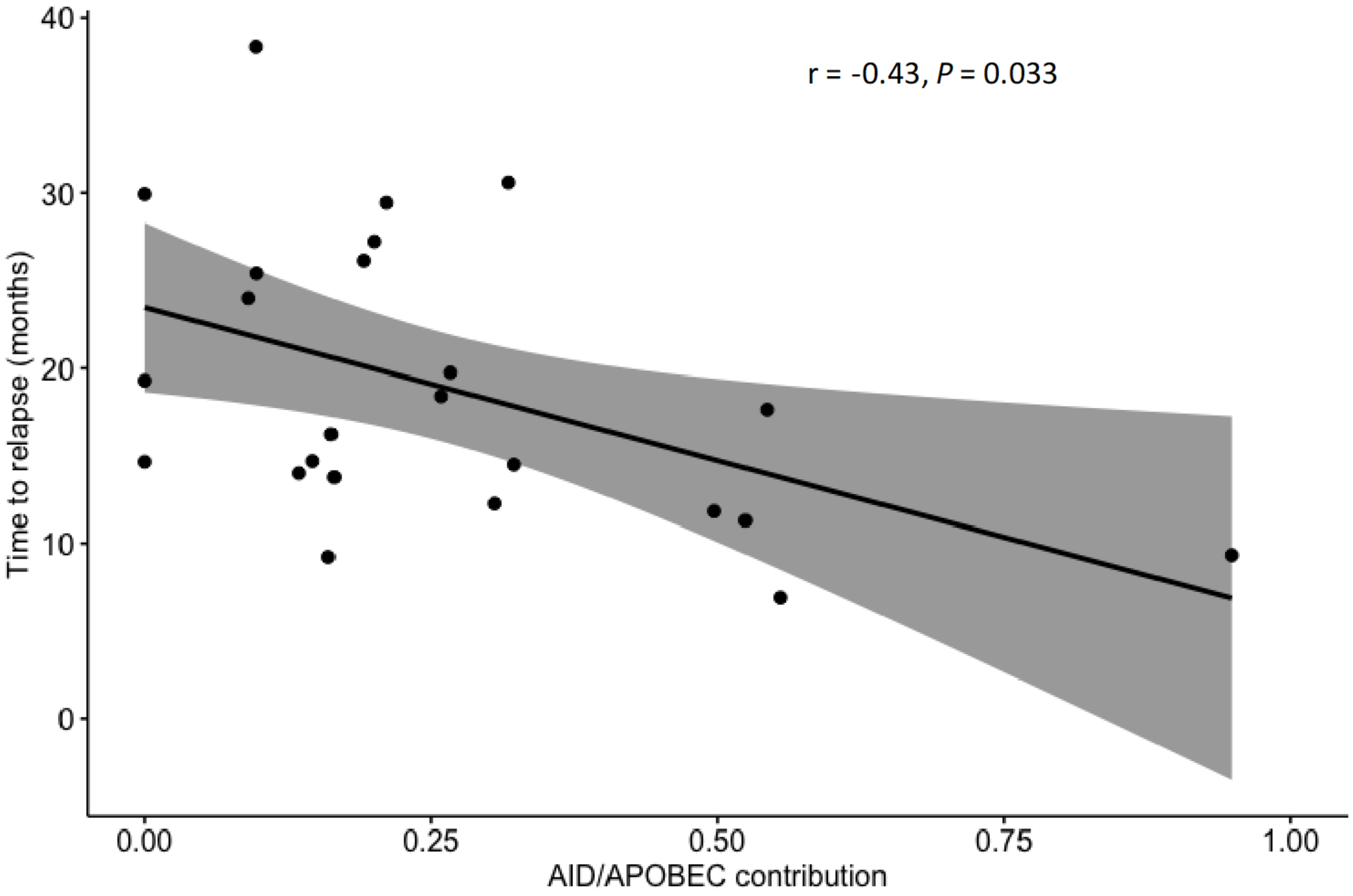

Supplementary Figure 20: Correlation between AID/APOBEC contribution at relapse and time to relapse (months). Scatter plot showing negative correlation (Spearman's correlation) between contribution of relapse-specific AID/APOBEC mutational and times to relapse for patients. The shaded area in grey indicates region of 95% confidence intervals.

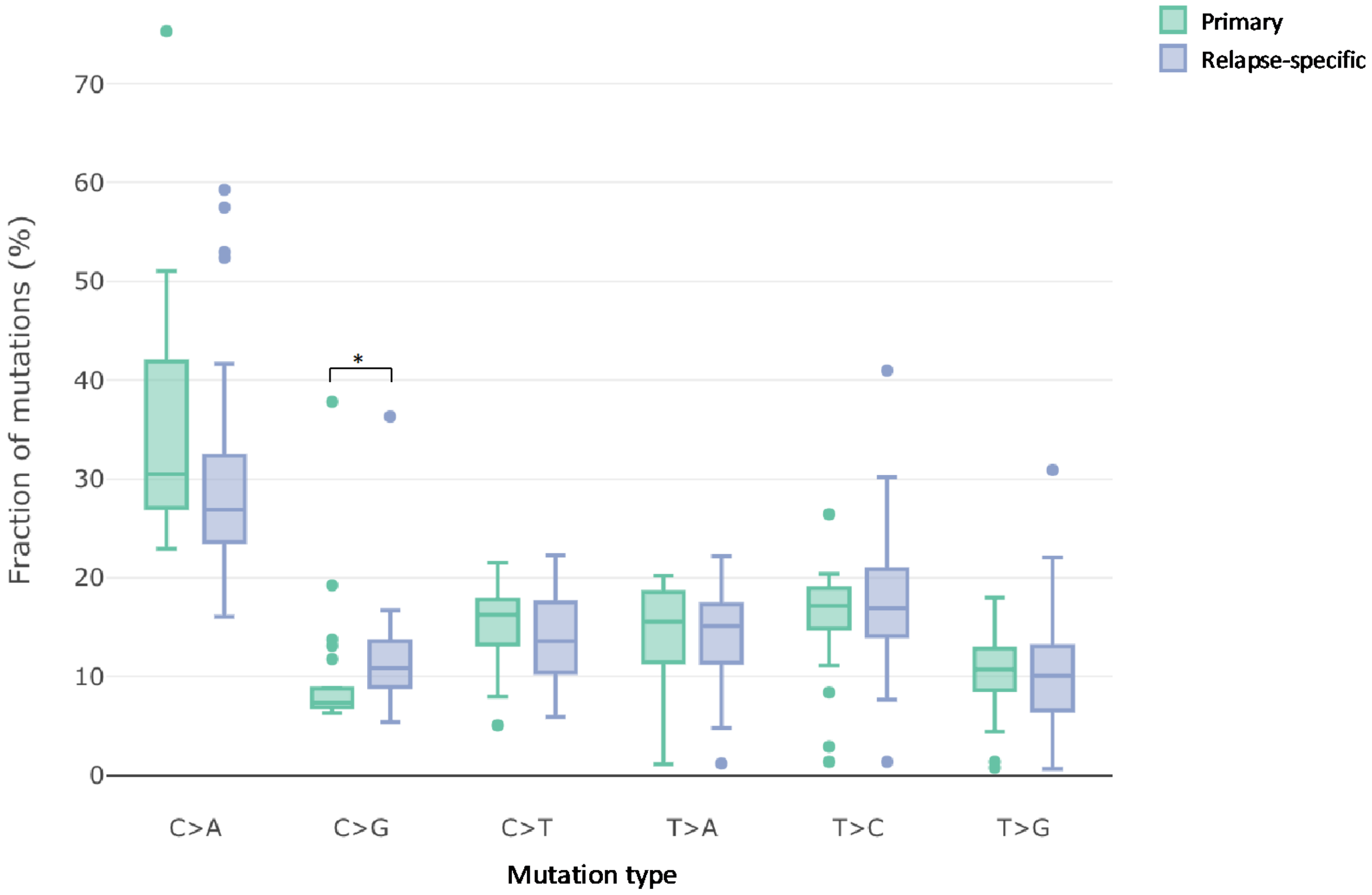

Supplementary Figure 21: Mutation types in primary versus relapse-specific mutations. Boxplots show proportions of different mutation types in primary and relapse-specific mutations. Whisker bars extend to $\pm$ 1.5 × interquartile range. *: $Q < 0.05$

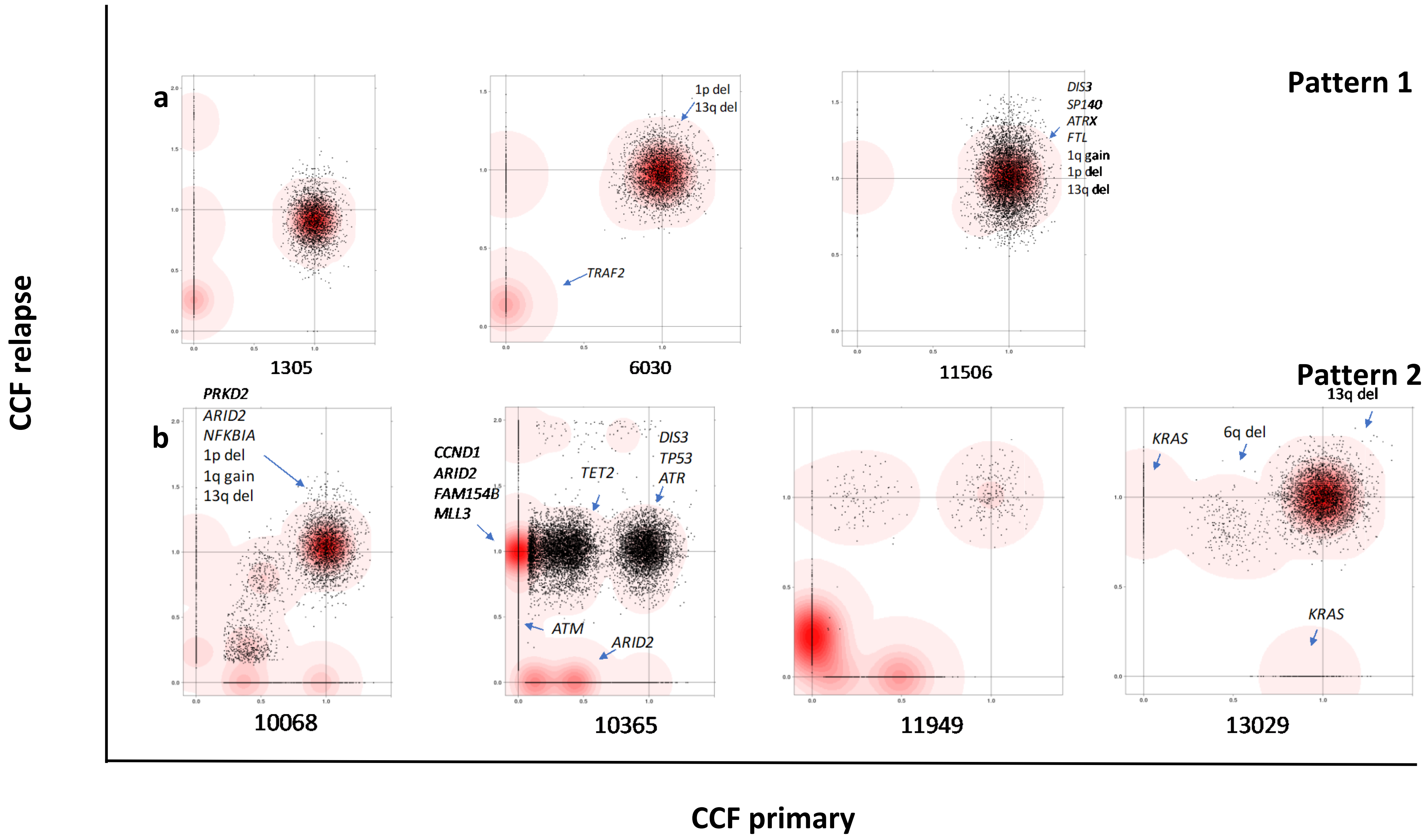
Pattern 1
Pattern 2
a
b
CCF relapse
CCF primary
1305
6030
11506
10068
10365
11949
13029
1p del
13q del
TRAF2
DIS3
SP140
ATRX
FTL
1q gain
1p del
13q del
PRKD2
ARID2
NFKBIA
1p del
1q gain
13q del
CCND1
ARID2
FAM154B
MLL3
TET2
DIS3
TP53
ATR
ATM
ARID2
KRAS
6q del
13q del
KRAS

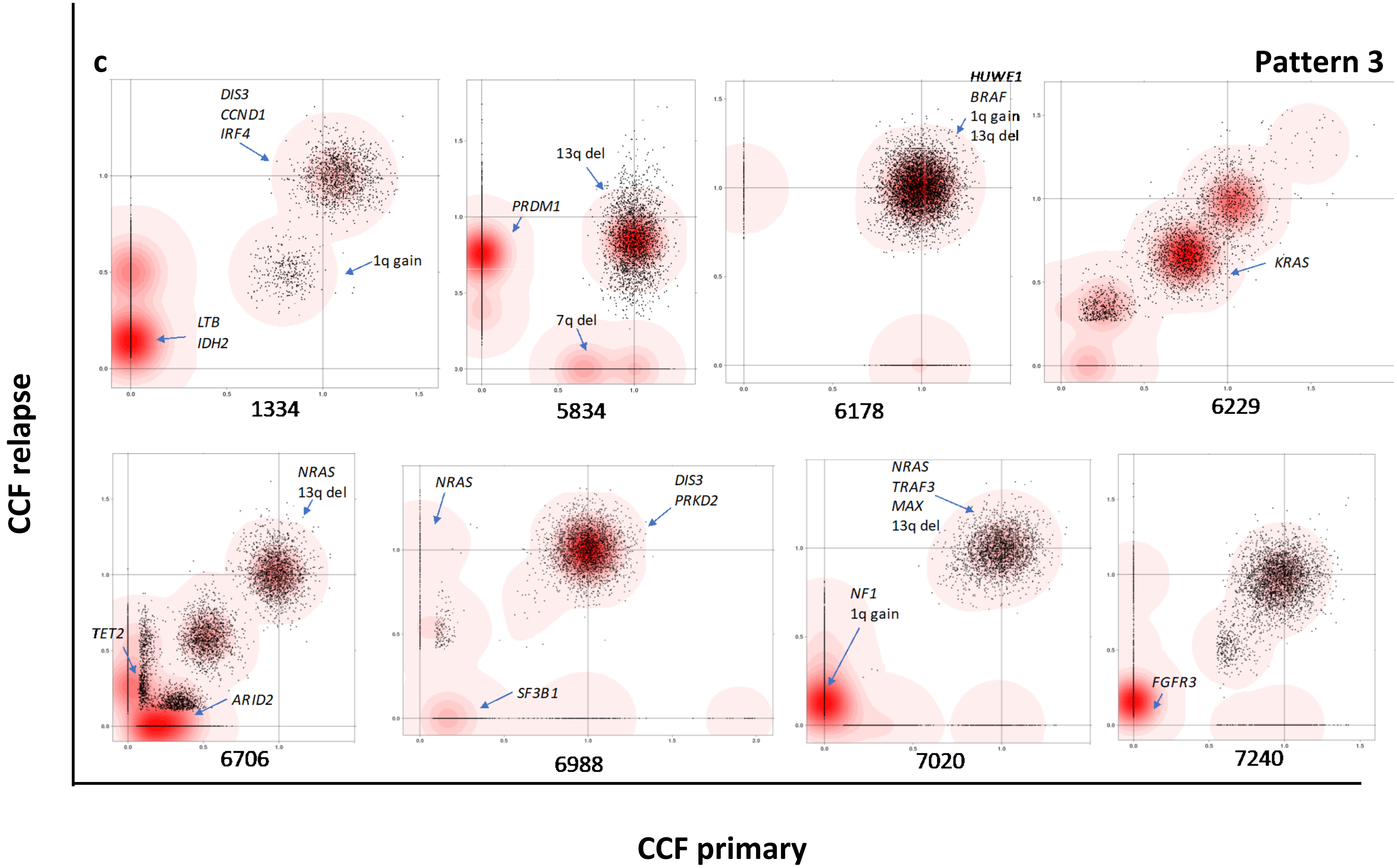
c
Pattern 3
CCF relapse
CCF primary
DIS3
CCND1
IRF4
1q gain
LTB
IDH2
1334
13q del
PRDM1
7q del
5834
HUWE1
BRAF
1q gain
13q del
6178
KRAS
6229
NRAS
13q del
TET2
ARID2
6706
NRAS
DIS3
PRKD2
SF3B1
6988
NRAS
TRAF3
MAX
13q del
NF1
1q gain
7020
FGFR3
7240

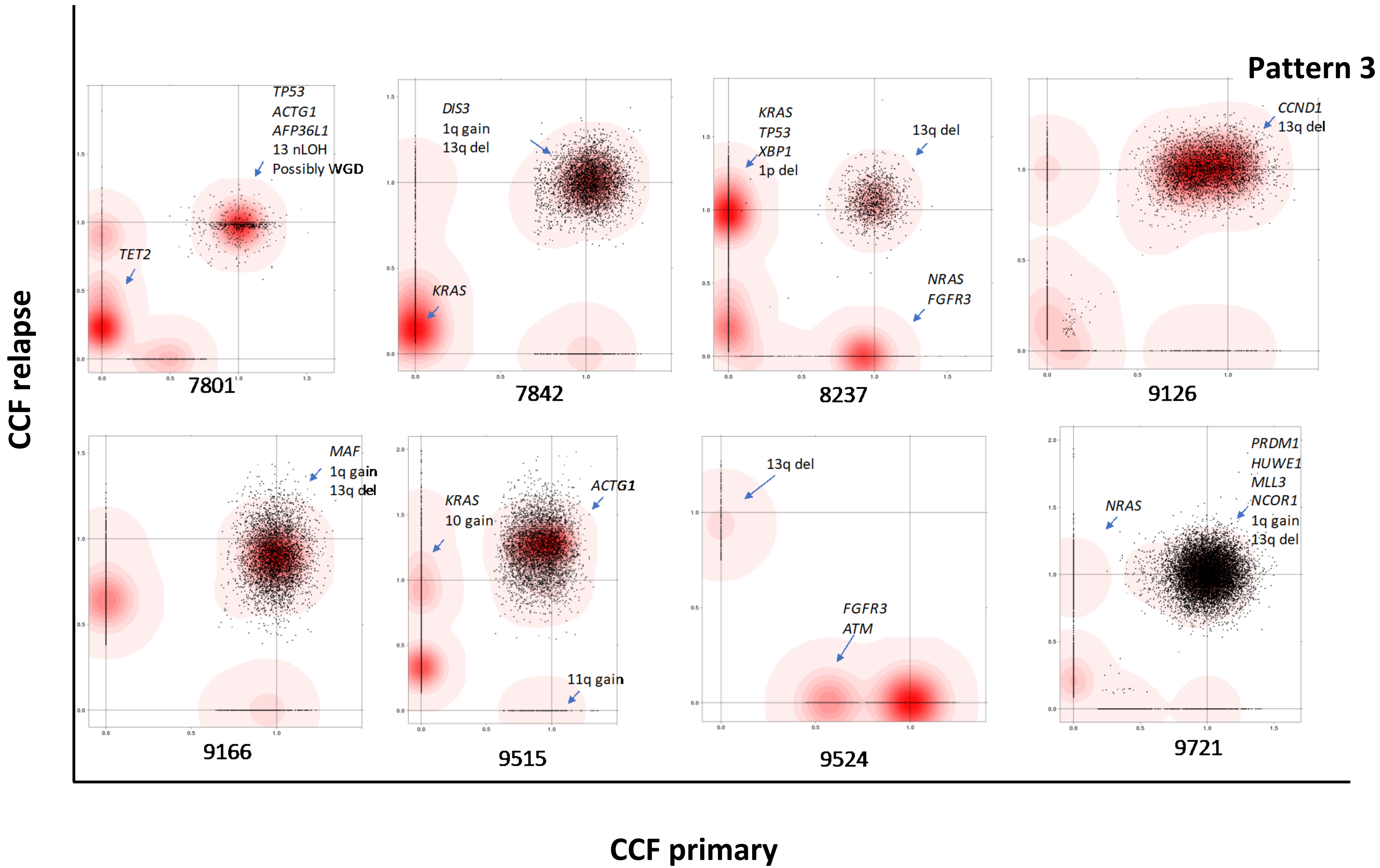
Pattern 3
CCF relapse
CCF primary
TP53
ACTG1
AFP36L1
13 nLOH
Possibly WGD
TET2
7801
DIS3
1q gain
13q del
KRAS
7842
KRAS
TP53
XBP1
1p del
13q del
NRAS
FGFR3
8237
CCND1
13q del
9126
MAF
1q gain
13q del
9166
KRAS
10 gain
ACTG1
11q gain
9515
13q del
FGFR3
ATM
9524
PRDM1
HUWE1
MLL3
NCOR1
1q gain
13q del
NRAS
9721

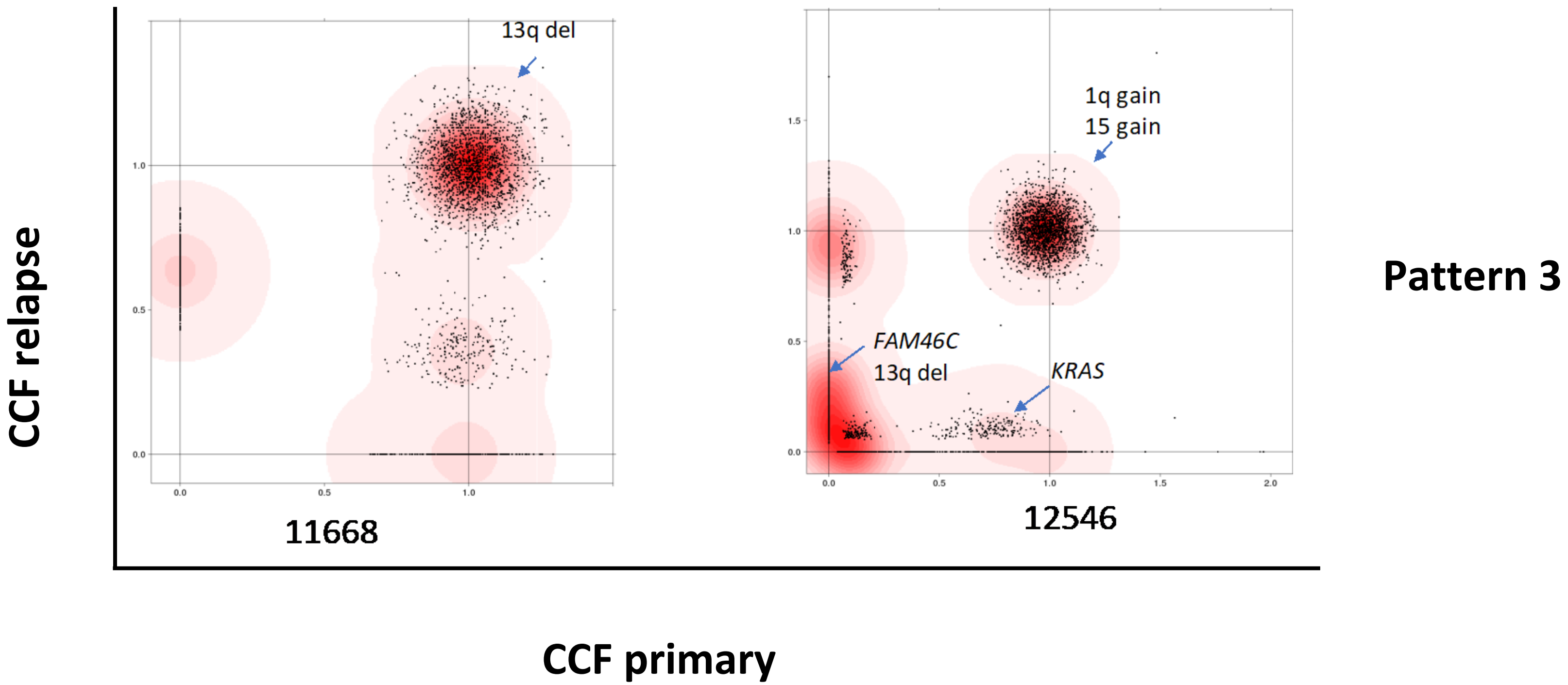

Supplementary Figure 22: Evolutionary trajectories of relapse in 24 relapsed tumours. Two-dimensional density plots showing the clustering of mutations (black dots). Darker red areas denote high posterior probability of a cluster (*i.e.* a clone). Clusters are annotated with coding driver mutations and major copy number alteration events. (a) Pattern 1: Dominant clone in primary gains additional mutations at relapse. (b) Pattern 2: A subclone survives and expands to become the dominant clone at relapse. (c) Pattern 3: Eradication or decline of one or more of primary clones and emergence of new clones not previously detected in primary. CCF, cancer cell fraction.
